# *Segatella* exacerbates chronic heart failure via TLR4/NF-κB pathway and therapeutic potential of low-carbohydrate diet

**DOI:** 10.1038/s41420-025-02762-9

**Published:** 2025-10-21

**Authors:** Aihaidan Abudouwayiti, Yan Xiao Li, Salamaiti Aimaier, Ying-Ying Zheng, Ailiman Mahemuti

**Affiliations:** Cardiovascular department of The First Affiliated Hospital of Xinjiang Medical University, Urumqi, Xinjiang China

**Keywords:** Biomarkers, Diseases

## Abstract

Chronic heart failure (CHF) is the end-stage of cardiovascular disease and is linked to intestinal dysbiosis, yet the precise microbial culprits and therapeutic targets remain unclear. Here, we show that the Gram-negative genus *Segatella* is selectively expanded in the gut of 152 CHF patients versus 105 matched controls, correlating with impaired cardiac function and disrupted lipid and amino-acid metabolism. Mechanistically, *Segatella* dose-dependently intensified doxorubicin-induced apoptosis and oxidative stress in H9c2 cardiomyocytes via TLR4/NF-κB signalling; these effects were reversed by co-culture in low-carbohydrate medium. In a doxorubicin-driven CHF rat model, *Segatella* gavage further reduced left-ventricular ejection fraction, aggravated fibrosis and heightened myocardial TNF-α and IL-6, whereas an 8-week low-carbohydrate diet (LCD) lowered *Segatella* abundance, improved cardiac function, reduced fibrosis, and alleviated pulmonary oedema. Collectively, *Segatella* exacerbates CHF by orchestrating TLR4/NF-κB-mediated inflammation and metabolic toxicity, while LCD confers protection by reshaping the gut microbiota, supporting microbiota-targeted, non-pharmacological therapy for CHF.

## Introduction

Cardiovascular disease (CVD) remains the leading cause of global mortality [1]. Chronic heart failure (CHF), the terminal stage of CVD, is characterized by myocardial injury, ventricular remodeling, and neuro-hormonal dysregulation, and confers high morbidity, mortality, and healthcare burden [2, 3]. Although contemporary pharmacotherapy—including angiotensin receptor–neprilysin inhibitors (ARNI) and SGLT2 inhibitors—and device-based interventions have improved outcomes, ~30% of patients with CHF continue to experience poor prognosis [4]. Recently, the gut–heart axis hypothesis has provided a novel framework for CHF research, implicating gut microbiota dysbiosis—via microbial translocation, metabolic derangements, and immune activation—in disease pathogenesis [5–7]. Nevertheless, the identification of specific pathobionts and the development of targeted microbiome-based therapies remain major unmet needs.

The gut microbiota communicates with the host cardiovascular system through bioactive metabolites. Lipopolysaccharide (LPS) derived from Gram-negative bacteria activates the Toll-like receptor 4 (TLR4)/NF-κB pathway, triggering the release of pro-inflammatory cytokines (e.g., TNF-α, IL-6) and apoptosis in cardiomyocytes [8]. Conversely, deficiency of beneficial metabolites such as short-chain fatty acids (SCFAs) aggravates myocardial energetic impairment [9]. While most investigations have emphasized global dysbiosis, the causal contribution of individual taxa—specifically *Segatella*—remains undefined. Although *Segatella* is markedly enriched in the gut of CHF patients, direct evidence linking its colonization to myocardial injury, either via secretion of toxic metabolites or modulation of host inflammatory cascades, is still lacking [10].

Low-carbohydrate diet (LCD) exerts cardioprotective effects by reshaping the gut microbiota. Short-term LCD interventions suppress the expansion of Gram-negative bacteria and selectively enrich SCFA-producing taxa, thereby reinforcing intestinal barrier integrity and attenuating systemic inflammation [11, 12]. Nevertheless, the specific impact of LCD on pathobionts such as *Segatella* within CHF models, as well as the dose-dependent relationship between microbiota modulation and myocardial protection, remains undefined [13, 14]. Building on this knowledge gap, the present study focuses on *Segatella*—a genus markedly enriched in CHF patients—and tests the hypothesis that *Segatella* aggravates myocardial injury through TLR4/NF-κB signaling, whereas LCD confers a synergistic “anti-bacterial, anti- inflammatory, and metabolic-regulatory” protection by reducing *Segatella* abundance and LPS biosynthesis.

Employing a stepwise translational pipeline—human profiling, mechanistic validation in vitro, and in vivo intervention—we compared the gut microbiome and metabolome of CHF patients and healthy controls to delineate correlations between *Segatella* abundance and cardiac function indices. In H9C2 cardiomyocytes, we delineated the molecular cascades by which *Segatella* induces oxidative stress, apoptosis, and inflammation. Finally, in doxorubicin-induced CHF rats, we assessed the cardioprotective efficacy of LCD and examined the mediating roles of microbiota-derived metabolites LPS and TMAO.

The key innovation of this study lies in providing the first systematic dissection of *Segatella* pathogenesis in CHF, advancing from correlative microbiome associations to causal mechanistic validation. We further define a dual-target paradigm for LCD intervention—suppressing pathobionts while simultaneously enriching beneficial metabolites—thereby delivering pre-clinical evidence for a non-pharmacological therapeutic strategy. By integrating multi-omics datasets across multiple experimental scales, we establish a trans-disciplinary pipeline linking “gut microbiota–metabolism–heart”, offering a new paradigm for precision cardiovascular medicine.

## Results

### Baseline characteristics of the study population

We first analyzed baseline characteristics of 152 CHF patients and 102 healthy controls (Supplementary Results-Table 1). From this overall cohort, a frequency-matched subset was generated by stratified random sampling according to age, sex, and NYHA functional class, yielding 50 CHF patients and 50 controls whose baseline data are presented in Table 1. Age and sex distributions were comparable between groups (mean age 58.5 vs 59.0 years, *P* = 0.778; 60% male in both, *P* = 0.95), confirming successful matching. CHF patients exhibited significantly higher BMI and greater prevalence of comorbidities—including type 2 diabetes mellitus and hypertension—than controls. Laboratory indices such as NT-proBNP and serum creatinine were also markedly elevated in CHF, indicating impaired cardiac and renal function.

**Table 1:** Comprehensive baseline characteristics of the randomly selected subgroup.

Characteristic	Control group (*n* = 50)	CHF group (*n* = 50)	χ²/z/t Value	*P* Value
Age (years)	58.5 ± 10.5	59.0 ± 9.8	0.8	0.778
Male (%)	30 (60)	30 (60)	0.0	0.95
BMI (kg/m²)	24.5 ± 3.6	27.6 ± 5.4	–3.5	0.002
Systolic BP (mmHg)	116.5 ± 11.5	117.0 ± 20.5	–0.2	0.639
Diastolic BP (mmHg)	72.5 ± 9.5	74.2 ± 13.5	–0.5	0.422
Heart rate (bpm)	80.5 ± 12.5	80.0 ± 15.0	0.1	0.939
Smoking history (%)	22 (44)	20 (40)	1.0	0.31
Alcohol history (%)	2 (4)	3 (6)	0.8	0.7
NYHA (%)				
I	–	0	–	–
II	–	6 (12)	–	–
III	–	25 (50)	–	–
IV	–	19 (38)	–	–
Laboratory parameters				
LVEF (%)	62.26 ± 4.62	38.17 ± 3.69	17.97	<0.001
NT-proBNP (ng/L)	113.33 ± 165.179	4585.89 ± 5256.977	–6.07	<0.001
WBC (*10⁹/L)	6.68 ± 1.73	6.36 ± 1.66	–0.896	0.112
Neutrophils (%)	62.84	57.65	3.021	0.0032
Lymphocytes (%)	25.66	32.09	3.948	<0.001
Hemoglobin (g/L)	132.56 ± 22.47	134.54 ± 24.06	–0.419	0.246
Platelets (*10⁹/L)	227.67 ± 74.28	230.70 ± 101.54	–0.913	0.072
CRP (mg/L)	4.50 (2.00, 8.00)	18.00 (10.00, 30.00)	–7.0	<0.001
K⁺ (mmol/L)	3.84 ± 0.6	3.96 ± 0.6	–2.037	0.042
Na⁺ (mmol/L)	140.01 ± 2.2	138.77 ± 3.5	–2.5	0.012
Apolipoprotein AI (g/L)	1.20 ± 0.10	1.10 ± 0.15	–2.0	0.045
AST (U/L)	10.66 ± 2.3	15.78 ± 3.45	3.5	<0.001
ALT (U/L)	15.67 ± 3.2	20.6 ± 4.5	3.0	0.003
Total protein (g/L)	70.98 ± 5.76	65.01 ± 6.22	–2.8	0.006
Creatinine (µmol/L)	66.61 (52.00, 87.00)	75.20 (63.83, 126.41)	–3.722	<0.001
Uric acid (µmol/L)	260.0 (230.0, 290.0)	350.0 (310.00, 390.0)	–4.5	<0.001
Triglycerides (mmol/L)	1.10 (0.87, 1.65)	1.17 (0.87, 1.62)	–0.119	0.905
Total cholesterol (mmol/L)	4.44 ± 0.78	3.81 ± 1.17	–2.193	0.025
HDL (mmol/L)	1.16 ± 0.19	1.06 ± 0.32	2.0	0.908
LDL (mmol/L)	2.80 ± 0.50	3.20 ± 0.60	–4.5	<0.001
Fasting blood glucose (mmol/L)	6.06 ± 2.1	6.22 ± 1.8	–0.096	0.924
Comorbidities				
Ischemic cardiomyopathy	0	25 (50)		<0.001
Hypertensive heart disease	0	14 (28)		<0.001
Valvular heart disease	0	6 (12)		<0.001
Dilated cardiomyopathy	0	5 (10)		<0.001
T2DM	3 (6)	20 (40)		<0.001
Hyperlipidemia	10 (20)	11 (22)		
Pulmonary hypertension	0	19 (0.38)		<0.001
Medication history				
ACEI/ARB (%)	0 (0)	45 (90)		<0.001
Diuretics (%)	0 (0)	45 (90)		<0.001
β-Blockers (%)	7 (14)	45 (90)		<0.001
SGLT-2 Inhibitors (%)	1 (2)	21 (42)		<0.001
Aspirin (%)	1 (2)	31 (62)		<0.001
Statins (%)	7 (14)	30 (60)		<0.001

Continuous variables are expressed as mean ± SD if normally distributed, or as median [interquartile range] otherwise. Categorical variables are presented as *n* (%). *P* values were calculated using the independent-sample *t*-test for normally distributed variables, the Mann–Whitney U test for non-normally distributed variables, and the χ² test for categorical variables.

*BMI* body mass index, *ACEI* angiotensin-converting-enzyme inhibitor, *ARB* angiotensin-receptor blocker, *SGLT-2i* sodium–glucose cotransporter-2 inhibitor. Controls, healthy control group; *CHF* chronic heart failure group.

### Gut microbiota OTU analysis

**Fig. 1:**
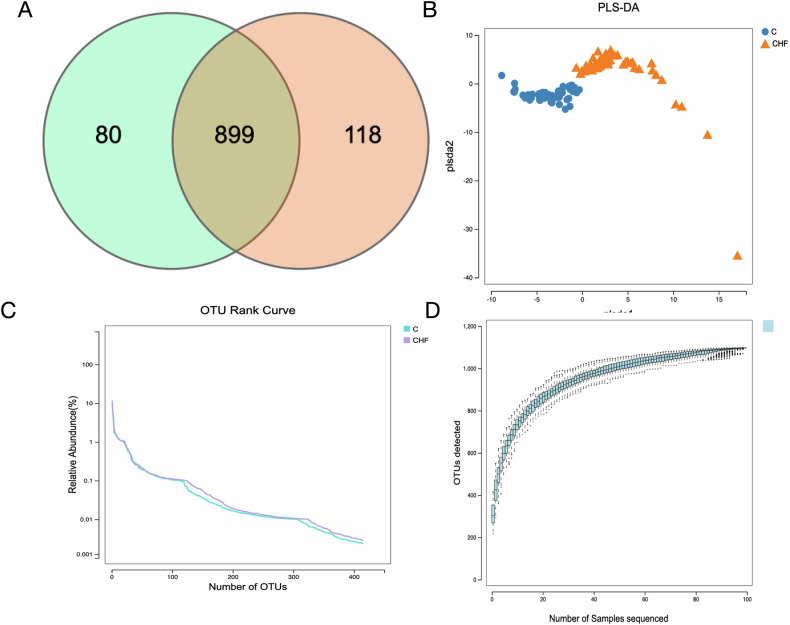
Operational taxonomic unit (OTU) analyses of gut microbiota in patients with chronic heart failure (CHF) and healthy controls. **A** Venn diagram of shared and unique OTUs: 899 OTUs were common to both groups, whereas 118 and 80 OTUs were exclusive to CHF and control subjects, respectively. **B** Species-accumulation curves: the plateau indicates sufficient sampling depth to capture the majority of microbial diversity. **C** Rank-abundance curves: comparable evenness and richness between groups confirm sequencing reliability. **D** PLS-DA score plot: CHF patients (orange triangles) and healthy controls (blue circles) form distinct clusters along the first two latent variables, underscoring markedly different gut microbial structures between the groups.

Operational taxonomic unit (OTU) clustering at a 97% sequence-similarity threshold was used to compare microbial composition between CHF patients and healthy controls. A Venn diagram revealed 899 shared OTUs, 118 unique to CHF, and 80 unique to controls (Fig. 1A). Species-accumulation curves plateaued, indicating adequate sampling depth (Fig. 1B), and rank-abundance curves confirmed comparable evenness and richness between groups, underscoring sequencing reliability (Fig. 1C). Partial least squares discriminant analysis (PLS-DA) showed clear separation of CHF and control samples along the first two latent variables (Fig. 1D), demonstrating markedly distinct gut microbial community structures in CHF patients.

### Alpha-diversity analysis of gut microbiota

**Fig. 2:**
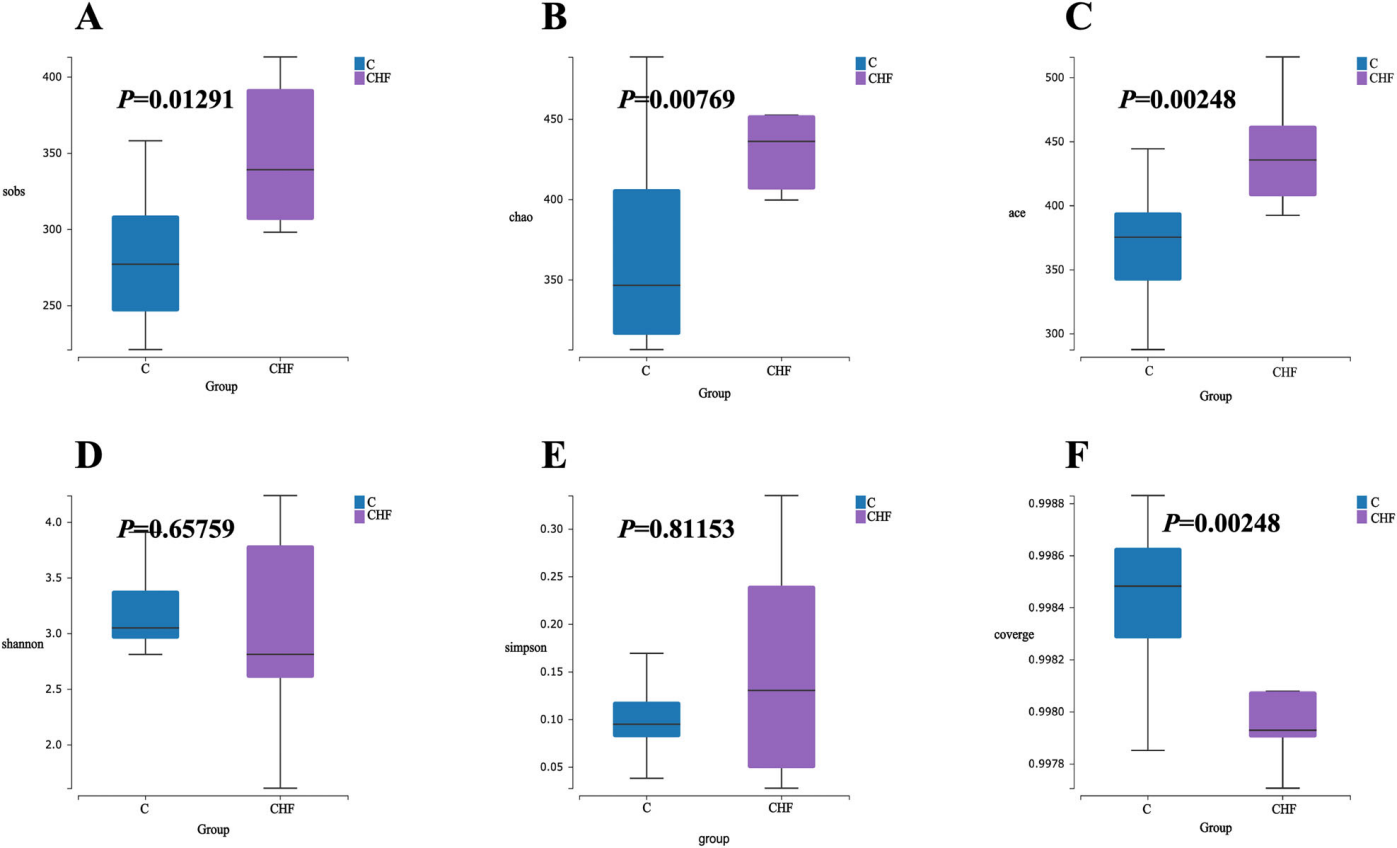
Alpha-diversity comparison of fecal microbiota between patients with chronic heart failure (CHF) and healthy controls. **A** Observed species (Sobs); **B** Chao1; **C** ACE; **D** Shannon; **E** Simpson; **F** Good’s coverage. Non-normally distributed data were analyzed using the Mann–Whitney U test; *P* < 0.05 was considered statistically significant.

Alpha-diversity metrics of fecal microbiota were compared between CHF patients and healthy controls. As shown in Fig. 2, the observed species (Sobs), Chao1, ACE, and Good’s coverage indices differed significantly between the two groups (*P* = 0.0129, 0.0077, 0.0025, and 0.0025, respectively). The Sobs index, reflecting the actual number of species detected, indicates a marked disparity in microbial richness. Similarly, the Chao1 and ACE estimators, which predict total species richness, corroborate that CHF is associated with altered microbial abundance. By contrast, Shannon and Simpson indices did not differ significantly (*P* = 0.6576 and 0.8115), suggesting that, despite shifts in richness, community evenness and diversity remain comparable between CHF patients and healthy individuals.

### Taxonomic discrepancies between groups

**Fig. 3:**
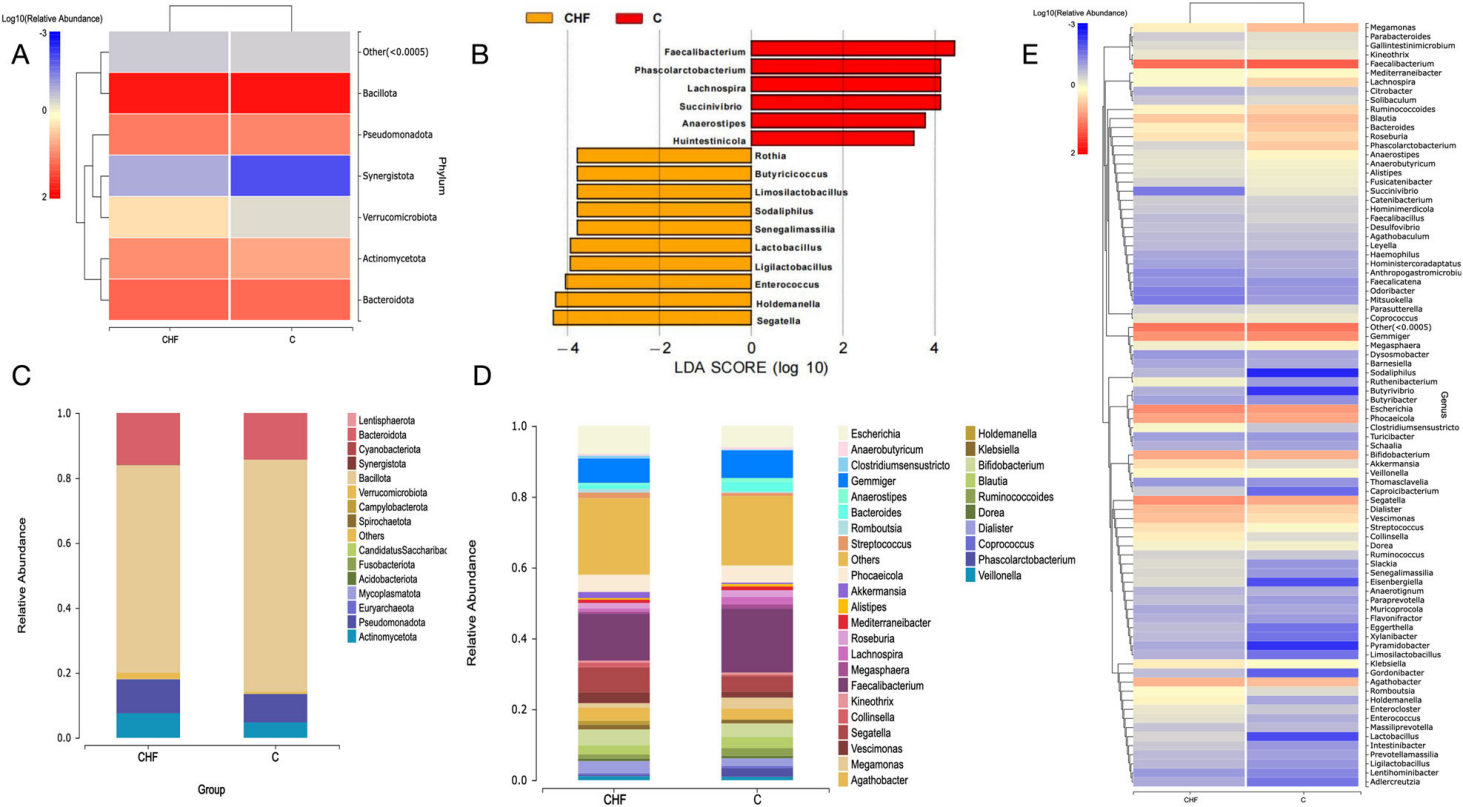
Differential gut microbial signatures between chronic heart failure (CHF) patients and healthy controls. **A** and **E** Heat maps depicting microbial composition at the phylum and genus levels, respectively; color intensity corresponds to log-transformed relative abundance, with darker hues indicating higher abundance. Average relative abundances at the phylum (**C**) and genus (**D**) levels; bar height precisely reflects the proportional representation of each microbial taxon within the respective cohort. **B** Linear discriminant analysis effect size (LEfSe) identifying taxa significantly associated with CHF (*P* < 0.05). Abundance data in bar charts and heat maps were compared using non-parametric tests (e.g., Mann–Whitney U test); *P* < 0.05 was considered statistically significant.

We compared fecal microbial composition between CHF patients and healthy controls at both phylum and genus levels. Figure 3 presents these findings: Heat maps (Fig. 3E) depict genus-level abundance on a Log10 (relative abundance) scale from −3 to 2, with darker colors indicating higher abundance; At the phylum level (Fig. 3C), *Actinobacteria* and *Proteobacteria* were markedly enriched in CHF, whereas *Fusobacteriota* and *Bacteroidetes* were reduced; At the genus level (Fig. 3D), *Segatella* and allied taxa were significantly expanded in CHF, while *Faecalibacterium* and other beneficial genera were depleted.

LEfSe-based taxonomic profiling was employed to identify bacterial taxa specifically associated with CHF. As shown in Fig. 3B, significant compositional differences between CHF patients and controls were detected (*P* < 0.05). At the genus level, *Faecalibacterium*, *Prevotella*, and *Lachnospira* were markedly enriched in healthy controls, whereas CHF patients exhibited significant expansion of *Segatella*, *Holdemanella*, *Enterococcus*, *Ligilactobacillus*, *Lactobacillus*, *Senegalimassilia*, *Sodaliphilus*, *Limosilactobacillus*, *Butyricicoccus*, and *Rothia*.

### Differential microbiota feature importance

**Fig. 4:**
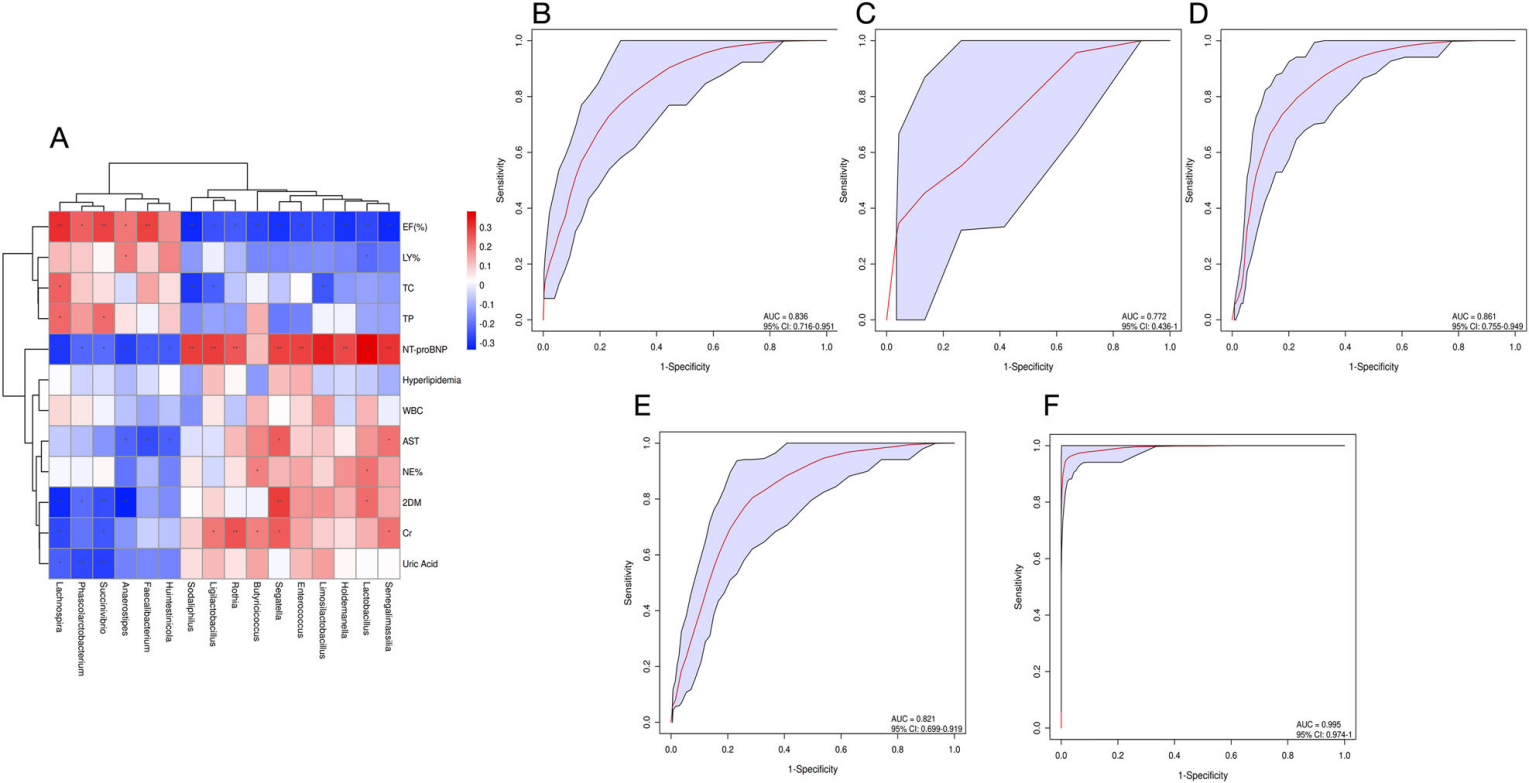
Microbial biomarker importance analysis. **A** Heat map illustrating correlations between differential bacteria and clinical indices; color intensity indicates correlation strength (red, positive; blue, negative). *P* < 0.05 denotes significance. **B** ROC curve and AUC for the training set. **C** ROC curve and AUC for the test set (AUC with 95% CI indicates predictive performance). **D** Mean ROC curve and AUC derived from tenfold cross-validation, reflecting overall diagnostic efficacy for CHF detection. **E** Combined model integrating age, BMI, total protein, and microbial biomarkers. **F** Extended model incorporating age, BMI, total protein, microbial biomarkers, NT-proBNP, and LVEF.

To elucidate the clinical relevance of the 16 discriminatory taxa, we correlated their abundances with key clinical variables. *Segatella* and other differential genera exhibited strong associations with LVEF, NT-proBNP, and concomitant type 2 diabetes mellitus (T2DM) but were only weakly linked to leukocyte or neutrophil percentages (Fig. 4A).

We further quantified the predictive value of these taxa using random forest with tenfold cross-validation. The 100 samples (50 CHF, 50 controls) were randomly partitioned into ten equal subsets; each iteration used nine subsets for training (45 CHF, 45 controls) and the remaining subset as the test set (5 CHF, 5 controls). ROC curves for the training and test sets yielded AUCs of 0.836 (95% CI 0.716–0.951, *P* < 0.001) and 0.772 (95% CI 0.436–1, *P* < 0.001), respectively (Fig. 4B, C). The overall AUC across all samples was 0.821 (95% CI 0.699–0.919, *P* < 0.001) (Fig. 4D), indicating robust diagnostic performance for CHF detection.

For the CHF clinical prediction model, candidate variables were first screened by univariate analysis and then subjected to binary logistic regression with stepwise selection. Age, BMI, and serum total protein emerged as significant predictors (*P* < 0.05; AUC > 0.5; Table 2), consistent with clinical reasoning: advancing age increases CHF risk, elevated BMI reflects obesity-related cardiac strain, and lower total protein mirrors systemic health decline. Subsequently, we constructed two integrated models. The combined model incorporated the three clinical variables—age, BMI, and total protein—together with microbial biomarkers, whereas the extended model further added NT-proBNP and LVEF. The combined model outperformed the microbiota-only model (AUC 0.861 vs 0.821, *P* < 0.001; Fig. 4E). The extended model achieved an AUC of 0.995 (95% CI 0.974–1.000, *P* < 0.001; Fig. 4F), significantly higher than the model lacking NT-proBNP and LVEF. Detailed parameters are provided in Supplementary Results-Tables 2 and 3.

**Table 2:** Candidate variables for clinical model development.

variables	B value	S.E	Wald value	*P* value	OR value	95%CI
Age	0.12	0.03	15.64	<0.001	1.13	1.06-1.20
BMI	0.28	0.08	11.48	0.001	1.33	1.13-1.56
Total protein	-0.13	0.04	11.99	0.001	0.88	0.81-0.94

Clinical variables significantly associated with CHF identified by univariate analysis. B, regression coefficient. S.E., standard error; Wald, Wald chi-square statistic; *P*, statistical significance; OR, odds ratio; 95% CI, 95% confidence interval.

### PICRUSt2-based functional profiling reveals altered gut microbial functions in CHF

**Fig. 5:**
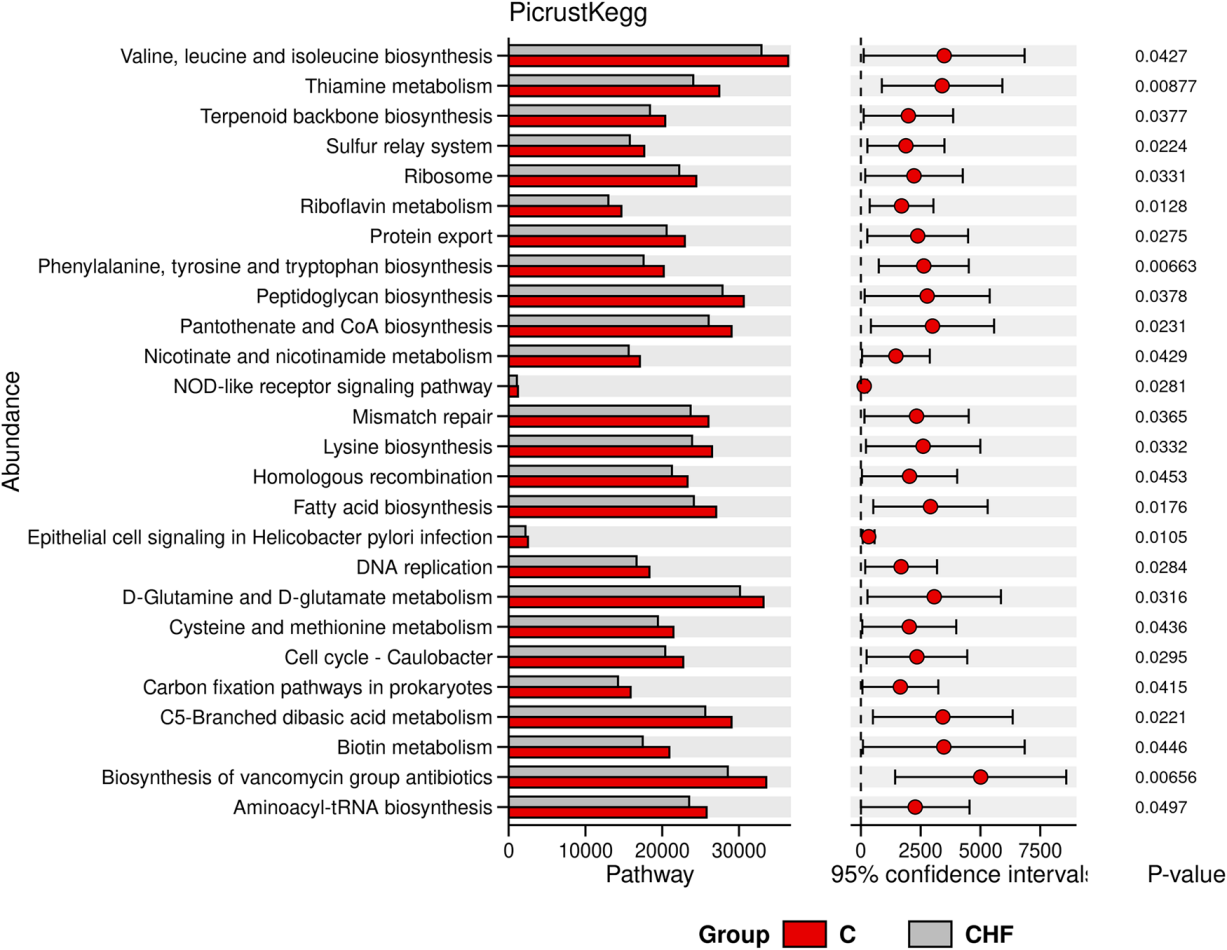
Differential KEGG functional abundances of gut microbiota between chronic heart failure (CHF) patients and healthy controls, as predicted by PICRUSt2. Bar charts display the predicted abundance of each KEGG metabolic pathway in healthy controls (C, red) and CHF patients (grey). The *P*-value for between-group differences is provided to the right of each bar, with horizontal lines indicating 95% confidence intervals. Pathways were selected if *P* < 0.05 and the abundance difference was significant. Mann–Whitney U test was used for non-normally distributed data; *P* < 0.05 was considered statistically significant.

Using PICRUSt2 to predict KEGG functional abundances in the gut microbiota of CHF patients and healthy controls (C), we observed a significant reduction in multiple microbial pathways in CHF (Fig. 5). Key down-regulated pathways included biosynthesis of vancomycin-group antibiotics, phenylalanine–tyrosine–tryptophan biosynthesis, thiamine metabolism, epithelial cell signaling in Helicobacter pylori infection, and riboflavin metabolism. These findings indicate a broad functional impairment of the gut microbiome in CHF, which may be closely linked to the pathophysiological state of the disease. Such alterations likely reflect diminished microbial capacities in nutrient metabolism, immune modulation, and cellular repair, thereby compromising microbial community balance and function.

### Identification of distinct serum metabolic alterations in CHF

**Fig. 6:**
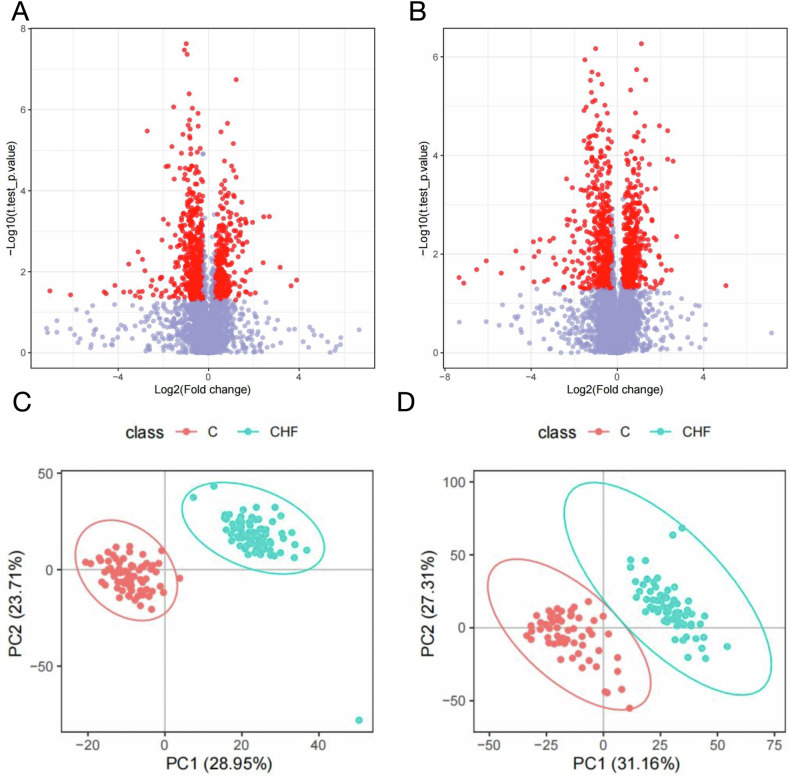
Identification of significant serum metabolic alterations in chronic heart failure (CHF). **A** Volcano plot of differential metabolites in positive-ion mode; **B** volcano plot in negative-ion mode. Fold change (FC) and *P* values are displayed; red dots denote up-regulated metabolites, blue dots down-regulated metabolites. Selection criteria: |FC| ≥ 1.2 or ≤0.8333 and FDR-adjusted *P* < 0.05; **C** PLS-DA score plot in positive-ion mode; **D** PLS-DA score plot in negative-ion mode, illustrating clear separation of CHF patients from healthy controls. Statistical analysis: two-tailed *t*-test with FDR correction to control the false discovery rate.

Untargeted serum metabolomics was performed using ultra-high-performance liquid chromatography coupled with tandem mass spectrometry (UHPLC-MS/MS) to delineate metabolic disparities between CHF patients and healthy controls. Volcano plots (Fig. 6A, B) display fold changes (FC) and *P* values; metabolites were considered significant at |FC | ≥ 1.2 or ≤0.8333 and FDR-adjusted *P* < 0.05 (two-tailed *t*-test). Marked metabolic divergence between groups was evident in both positive- and negative-ionization modes. Subsequent PLS-DA (Fig. 6C, D) revealed clear segregation of metabolic profiles, underscoring the potential of serum metabolites to discriminate CHF patients from healthy individuals. These findings implicate specific metabolite perturbations in CHF pathophysiology and provide a rationale for biomarker discovery.

### Metabolite identification and classification

**Fig. 7:**
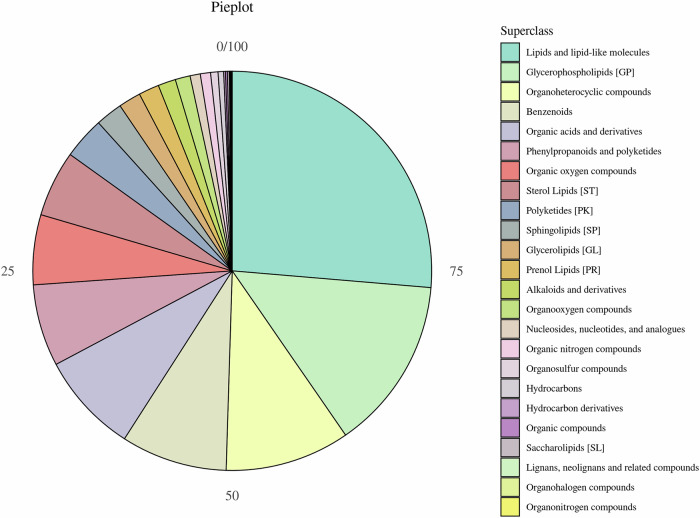
Chemical classification of identified serum metabolites. The stacked bar chart depicts the proportional distribution of the 2097 metabolites across chemical classes in both positive- and negative-ion modes. Lipids and lipid-like molecules account for 26.34% (349 compounds), and glycerophospholipids (GP) represent 14.04% (186 compounds), highlighting the predominance of lipid-related species.

Using LC-MS/MS, we identified 920 metabolites in positive-ion mode and 1177 in negative-ion mode, yielding a total of 2097 distinct features (Fig. 7). Chemical classification revealed that lipids and lipid-like molecules (26.34%, 349 compounds) and glycerophospholipids (GP, 14.04%, 186 compounds) were the dominant categories, underscoring the potential role of lipid dysregulation in CHF pathophysiology.

To pinpoint biologically relevant discriminatory metabolites, we applied a PLS-DA model and implemented the following criteria: (1) |fold change| ≥ 1.2 or ≤0.8333; (2) *P* < 0.05; and (3) variable importance in projection (VIP) > 1. Applying these thresholds, we retained 47 significant differential metabolites—24 in positive-ion mode and 23 in negative-ion mode (Supplementary Results-Tables 4 and 5).

### Evaluation of differential metabolites as potential biomarkers

**Fig. 8:**
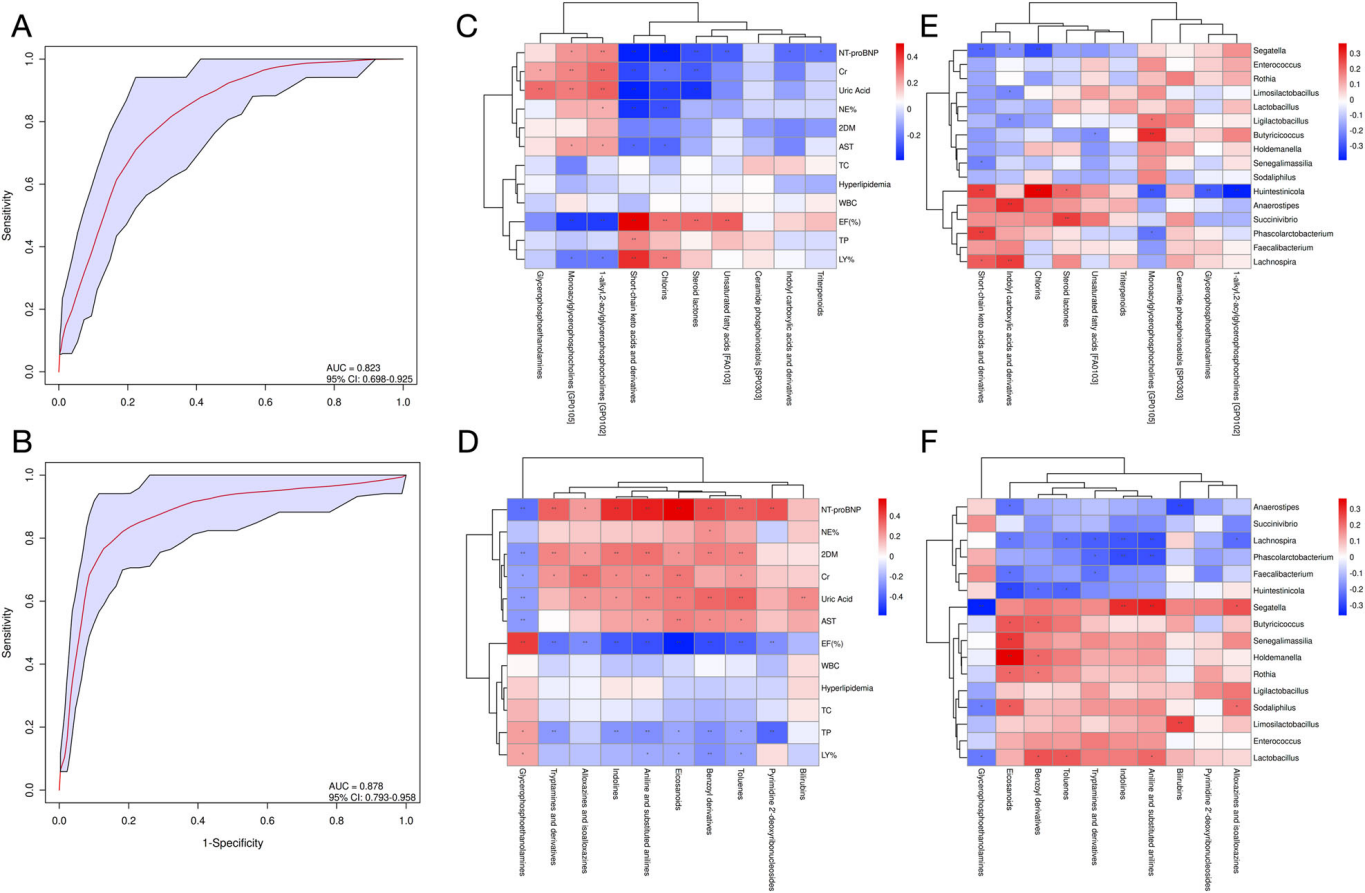
Integrative analysis of differential metabolites. **A** ROC curves for the top 10 discriminating metabolites in positive-ion mode. **B** ROC curves for the top 10 metabolites in negative-ion mode. AUC values and 95% CIs quantify diagnostic accuracy. **C** Heat map of correlations between positive-ion metabolites and clinical indices. **D** Heat map of correlations between negative-ion metabolites and clinical indices. **E** Heat map of correlations between positive-ion metabolites and differential bacterial taxa. **F** Heat map of correlations between negative-ion metabolites and differential bacterial taxa. Color intensity reflects Spearman correlation strength (red, positive; blue, negative). ROC analyses assessed diagnostic performance; Spearman rank correlation was used for all associations, with *P* < 0.05 considered statistically significant.

Given the marked divergence in serum metabolomes between CHF patients and controls, we sought to identify metabolites with the greatest discriminatory power for CHF. Differential features were ranked by their variable importance in projection (VIP) scores, and the top ten metabolites from each ionization mode were subjected to ROC analysis (Fig. 8A, B). Positive-ion mode: Short-chain keto acids and derivatives, monoacylglyceroph-osphocholines (GP0105), glycerophosphoethanolamines, 1-alkyl-2-acylglycerophosphocholi -nes(GP0102), indolylcarboxylic acids and derivatives, chlorins, steroid lactones, unsaturated fatty acids (FA0103), triterpenoids, and ceramide phosphoinositols (SP0303).Negative-ion mode: Glycerophosphoethanolamines, indolines, aniline and substituted anilines, benzoyl derivatives, toluenes, tryptamines and derivatives, pyrimidine 2′-deoxyribonucleosides, eicosanoids, bilirubins, and alloxazines/isoalloxazines. ROC curves yielded AUCs > 0.8 for both ionization modes (positive-ion AUC = 0.823, 95% CI 0.698–0.925; negative-ion AUC = 0.878, 95% CI 0.793–0.958), demonstrating robust diagnostic accuracy. Detailed parameters are provided in Supplementary Results-Table 6.

We next correlated the ten most discriminatory positive-ion metabolites with clinical parameters (Fig. 8C). Short-chain keto acids and derivatives exhibited a significant positive association with LVEF (*r* = 0.45, *P* < 0.05) and an inverse association with NT-proBNP (*r* = −0.38, *P* < 0.05), suggesting that their abundance reflects cardiac functional status. Conversely, 1-alkyl-2-acylglycerophosphocholines(GP0102) were negatively correlated with LVEF (*r* = −0.42, *P* < 0.05) and positively correlated with NT-proBNP (*r* = 0.39, *P* < 0.05), implicating these phospholipids in impaired pump function.

Negative-ion metabolites were similarly analyzed (Fig. 8D). Glycerophosphoethan -olamines displayed a negative correlation with NT-proBNP (*r* = −0.40, *P* < 0.05) and a positive correlation with LVEF (*r* = 0.43, *P* < 0.05), again linking their levels to cardiac performance. Toluenes were inversely associated with LVEF (*r* = −0.41, *P* < 0.05), further supporting a relationship between these metabolites and declining cardiac output.

Integration of metabolomic and metagenomic data (Fig. 8E, F) revealed that short-chain keto acids and derivatives were positively correlated with *Segatella* abundance (*r* = 0.38, *P* < 0.05), whereas unsaturated fatty acids (FA0103) were negatively correlated with Butyricicoccus (*r* = −0.40, *P* < 0.05), indicating that metabolite fluctuations are linked to specific microbial taxa.

### Metabolic pathway analysis

**Fig. 9:**
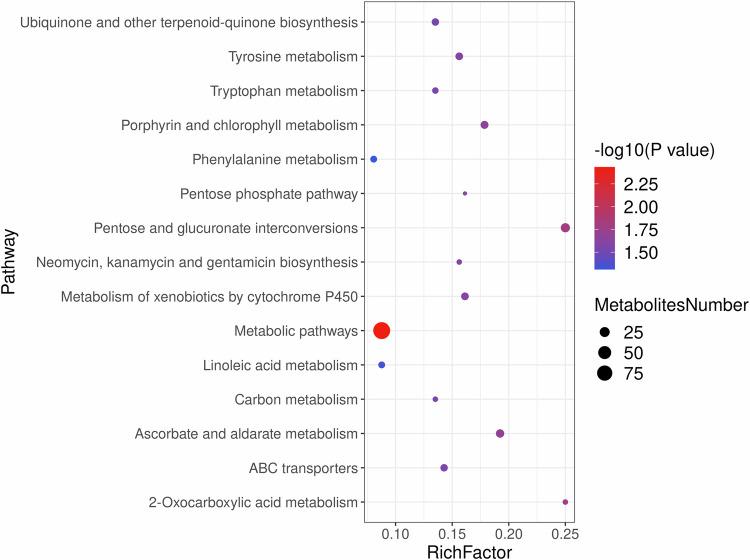
KEGG-based metabolic pathway enrichment bubble plot. The *x*-axis represents the enrichment factor (ratio of observed to total metabolites in each pathway); the *y*-axis lists analyzed pathways. Bubble color indicates statistical significance (-log10 *P* value), with deeper red denoting lower *P* values; bubble size reflects the number of enriched metabolites.

Using the KEGG database, we annotated serum metabolites from CHF patients and performed pathway enrichment analysis. Bubble plots (Fig. 9) reveal significant enrichment of ubiquinone and other terpenoid-quinone biosynthesis, tyrosine metabolism, tryptophan metabolism, porphyrin and chlorophyll metabolism, phenylalanine metabolism, pentose phosphate pathway, pentose and glucuronate interconversions, neomycin/ kanamycin/gentamicin biosynthesis, metabolism of xenobiotics by cytochrome P450, metabolic pathways, linoleic acid metabolism, carbon metabolism, ascorbate and aldarate metabolism, ABC transporters, and 2-oxocarboxylic acid metabolism.

### Ex vivo results

To verify the direct effect of *Segatella* on cardiomyocytes and the intervention effect of LCD, this study used the H9c2 rat cardiomyocyte injury model induced by Dox, combined with different concentrations of *Segatella* treatment and LCD intervention, to analyze apoptosis, oxidative stress, and molecular mechanisms. The results are as follows:

1. *Segatella* dose-dependently exacerbates cardiomyocyte apoptosis, which is partially reversed by LCD.

**Fig. 10:**
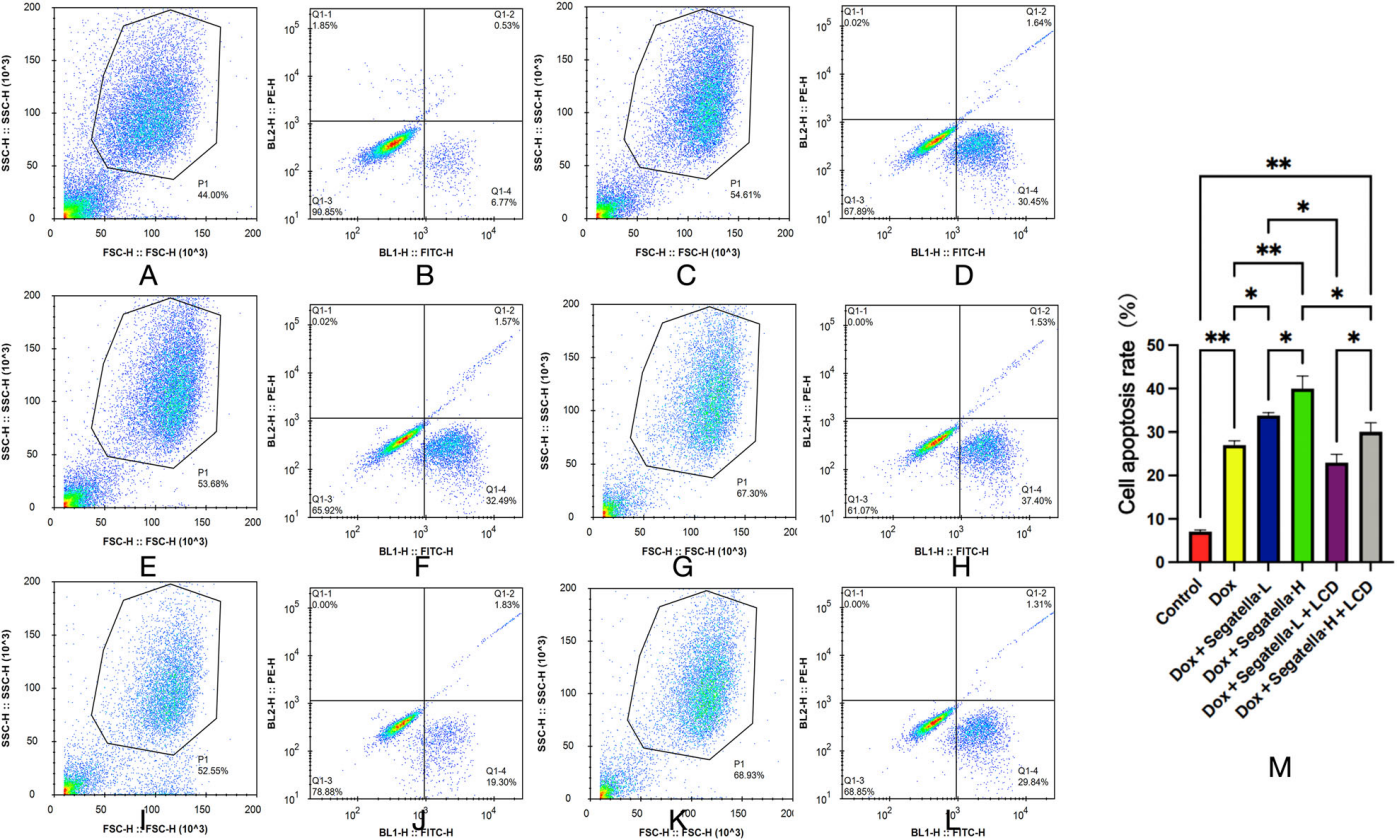
Flow cytometry analysis of cardiomyocytes under various intervention conditions. FSC-H indicates forward scatter height, which measures cell size; BL1-H (FITC-H) represents Annexin V/FITC fluorescence intensity, used to detect apoptosis-related indicators. **A** and **B** show the normal control group, **E** and **F** show the Dox + *Segatella*-L group, and **I** and **J** show the Dox + *Segatella*-L+LCD group. **M** shows the apoptosis rate of cardiomyocytes under different intervention conditions, with the *x*-axis representing different experimental groups and the *y*-axis representing the apoptosis rate (%). Error bars above each bar indicate standard deviation. Statistical analysis: s indicates no difference, * indicates significance, * indicates *P* < 0.05, ** indicates *P* < 0.01, *** indicates *P* < 0.001.

Flow cytometry results of Annexin V-FITC/PI double staining (Fig. 10) showed that Dox treatment (1 μM, 24 h) significantly induced cardiomyocyte apoptosis (*P* < 0.01). After combined treatment with *Segatella*, the apoptosis rate further increased in a dose-dependent manner. LCD intervention (20% low-glucose medium) significantly inhibited apoptosis (*P* < 0.05), but the rate remained higher than that of the normal control group (*P* < 0.01).

2. *Segatella* induces oxidative stress injury, and LCD improves cellular redox balance.

**Fig. 11:**
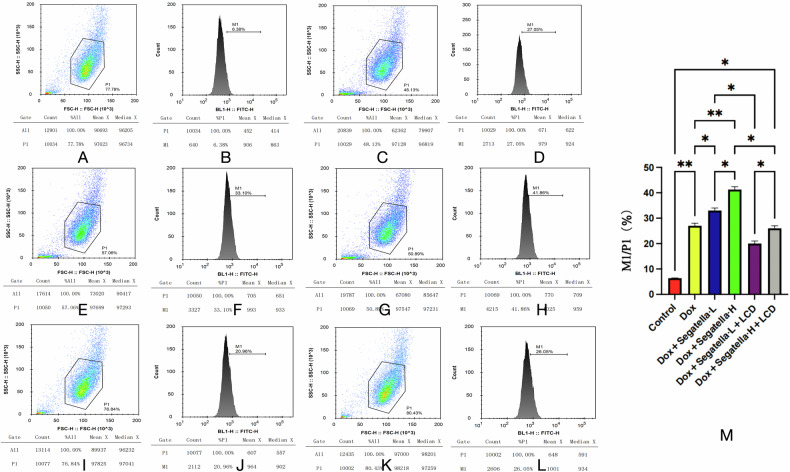
Flow cytometry analysis of reactive oxygen species (ROS) levels in cardiomyocytes from different treatment groups. **A** and **B** show the normal control group, **E** and **F** show the Dox + *Segatella*-L group, and **I** and **J** show the Dox + *Segatella*-L+LCD group. **M** shows the percentage of M1 population in P1 gate of cardiomyocytes under different intervention conditions, with the *x*-axis representing different experimental groups and the *y*-axis representing the percentage of M1 population in P1 (%). Error bars above each bar indicate standard deviation. Statistical analysis: s indicates no difference, * indicates significance, * indicates *P* < 0.05, ** indicates *P* < 0.01, *** indicates *P* < 0.001.

DCFH-DA fluorescence probe detection showed (Fig. 11) that the intracellular ROS level in the Dox group was significantly increased, with the high ROS population (M1 subpopulation) accounting for 27.05% (3.2 times higher than the control group, *P* < 0.01). *Segatella* treatment exacerbated oxidative stress: the proportion of M1 subpopulation in the high-concentration bacterial group increased to 41.86% (an increase of 54.6% compared with the Dox group, *P* < 0.01), and in the low-concentration bacterial group to 33.1% (*P* < 0.05). LCD intervention reduced ROS accumulation: the proportion of M1 subpopulation in the low-concentration bacterial + LCD group decreased to 20.96% (*P* < 0.01 vs. bacterial group alone), and in the high-concentration bacterial + LCD group to 26.05% (*P* < 0.05).

3. *Segatella* activates the TLR4/NF-κB pathway, and LCD inhibits the over- activation of the pathway.

**Fig. 12:**
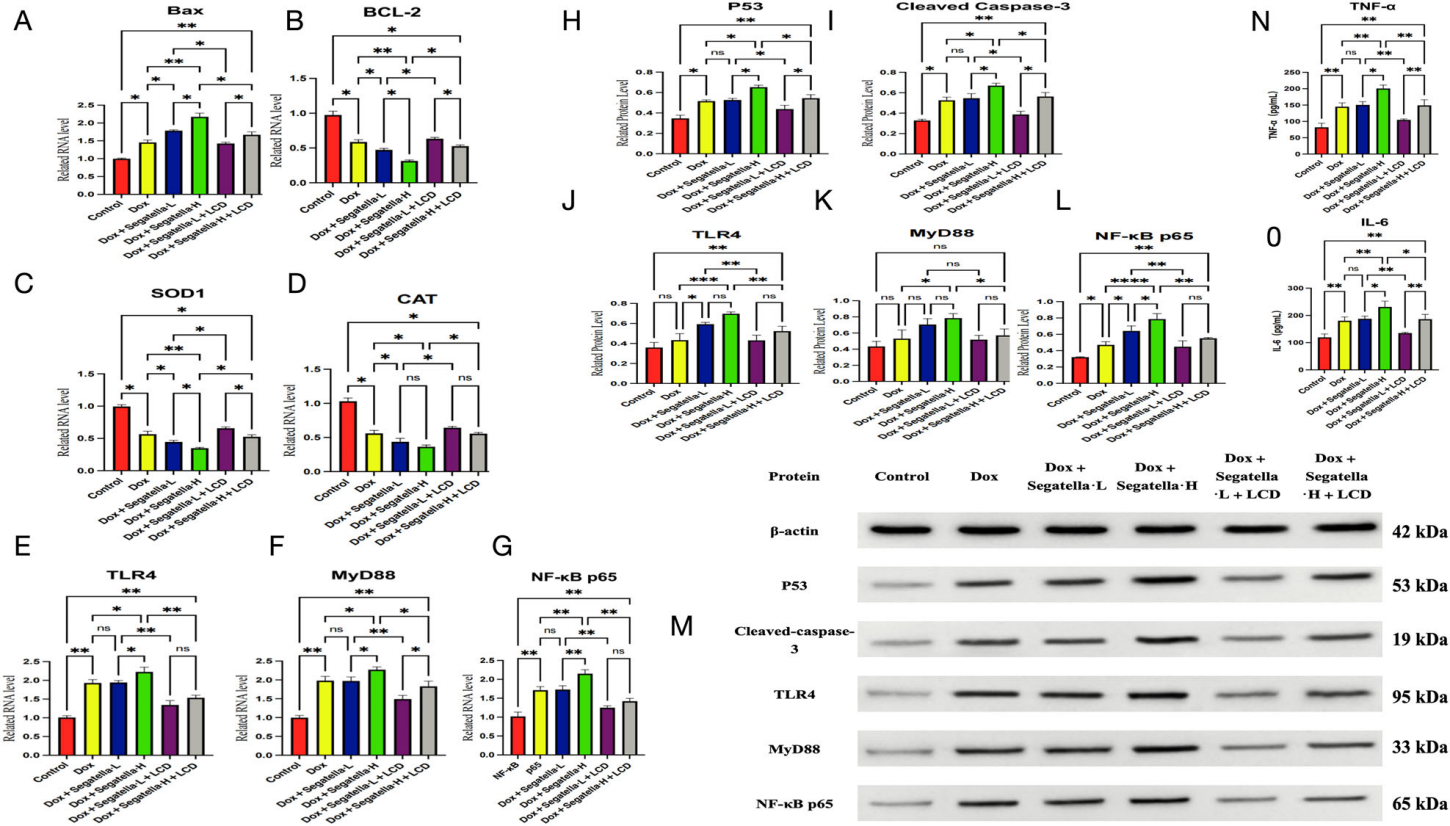
qPCR, WB, and ELISA results of different treatment groups. **A**–**G** show bar charts of relative RNA levels of Bax, Bcl-2, SOD1, CAT, TLR4, MyD88, and NF-κB p65 in different treatment groups. **H**–**L** present bar charts of relative protein levels of p53, cleaved caspase-3, TLR4, MyD88, and NF-κB p65 (WB), and **M** shows the corresponding Western blot bands. **N** and **O** depict bar charts of TNF-α and IL-6 concentrations (ELISA) in different treatment groups. Statistical analysis: s indicates no difference, * indicates significance, * indicates *P* < 0.05, ** indicates *P* < 0.01, *** indicates *P* < 0.001.

The results of molecular mechanism detection (Fig. 12) showed that:

Apoptosis and antioxidant genes: High-concentration *Segatella* significantly upregulated the pro-apoptotic gene Bax (*P* < 0.05) and downregulated the anti-apoptotic gene Bcl-2 (*P* < 0.05), increasing the Bax/Bcl-2 ratio and inhibiting the antioxidant genes SOD1 and CAT (*P* < 0.05). LCD intervention reversed these changes (*P* < 0.05).

TLR4/NF-κB pathway: High-concentration *Segatella* significantly upregulated the mRNA and protein expression of TLR4 and NF-κB p65 (*P* < 0.05), while the change in MyD88 protein was not significant (*P* > 0.05). LCD inhibited the activation of TLR4 and NF-κB p65 (*P* < 0.05).

Inflammatory cytokines: In the high-concentration *Segatella* group, the levels of TNF-α and IL-6 in the cell supernatant were significantly increased compared with the Dox group (*P* < 0.01), and LCD intervention reduced both (*P* < 0.05).

In summary, *Segatella* dose-dependently exacerbates Dox-induced cardiomyocyte apoptosis and oxidative stress via the TLR4/NF-κB pathway and upregulates pro-inflammatory cytokine release. LCD can partially reverse *Segatella*-mediated cardiomyocyte injury by inhibiting this pathway, reducing ROS accumulation, and attenuating the inflammatory response.

### In vivo validation in rats

In our preliminary studies, multi-omics approaches revealed a strong correlation between *Segatella* in the gut of CHF patients and cardiomyocyte injury, with the bacterium exacerbating apoptosis and oxidative stress. LCD intervention effectively attenuated these detrimental effects. However, in vitro experiments cannot fully replicate the in vivo environment, and the interactions between *Segatella* and other microbiota, as well as the long-term effects of LCD, remain to be elucidated. Therefore, this study utilized Wistar rats to establish a Dox-induced CHF model to investigate the in vivo mechanisms of *Segatella* and the long-term effects of LCD intervention. This animal model provides a basis for optimizing CHF treatment strategies and facilitates the translation of research findings into clinical applications.

### General condition and weight changes in rats (Fig. 13)

**Fig. 13:**
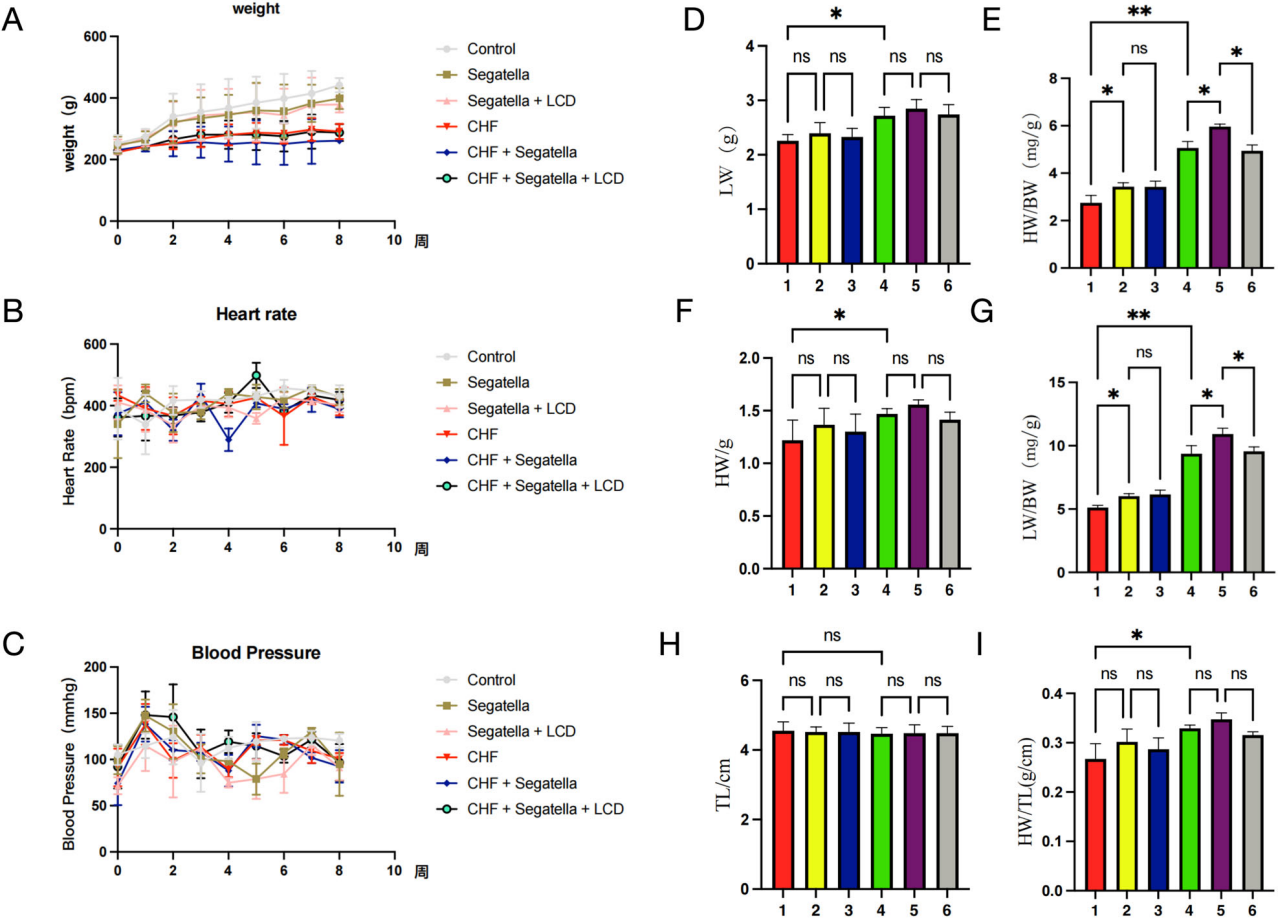
General condition and weight changes in rats across different groups. **A** Weight change curves of rats in each experimental group; **B** Heart rate change curves over time for each experimental group; **C** Mean arterial pressure change curves over time for each experimental group; **D** Heart weight (g) of rats in each experimental group; **E** Ratio of heart weight (mg) to body weight (g), i.e., heart index, in each experimental group; **F** Lung weight (g) of rats in each experimental group; **G** Ratio of lung weight (mg) to body weight (g), i.e., lung index, in each experimental group; **H** Tibia length (cm) of rats in each experimental group; **I** Ratio of heart weight (g) to tibia length (cm) in each experimental group. The numbers 1–6 on the *x*-axis represent: 1: Control; 2: *Segatella*; 3: *Segatella*+LCD; 4: CHF; 5: CHF + *Segatella*; 6: CHF + *Segatella*+LCD. Sample size was 6 rats per group. Statistical analysis: s indicates no difference, * indicates significance, * indicates *P* < 0.05, ** indicates *P* < 0.01, *** indicates *P* < 0.001.

Body Weight (Fig. 13A):

The body weight of the normal control group increased steadily over the experimental period (final weight 441.5 ± 23.2 g). In contrast, the CHF model group exhibited a significant decrease in body weight (291.3 ± 24.5 g, *P* < 0.01 vs. control group), with an even more pronounced reduction in the CHF+*Segatella* group (261.0 ± 10.6 g, *P* < 0.01 vs. CHF group). LCD intervention partially alleviated this weight loss, with the final weight of the CHF+*Segatella*+LCD group being 287.5 ± 27.9 g (*P* < 0.05 vs. CHF+*Segatella* group).

Heart rate and blood pressure (Fig. 13B, C):

In the CHF group, heart rate increased over the course of the disease, while blood pressure initially rose and then declined. The CHF+*Segatella* group showed a more significant increase in heart rate (388.9 ± 26.8 beats/min, *P* < 0.05 vs. CHF group) and a more pronounced decrease in blood pressure (92.6 ± 17.5 mmHg, *P* < 0.05 vs. CHF group). LCD intervention stabilized both heart rate and blood pressure (*P* < 0.05 vs. CHF+*Segatella* group).

Organ Indices (Figs. D–I):

Heart weight-to-body weight ratio (HW/BW): The CHF group had a significantly higher HW/BW ratio (5.06 ± 0.28 mg/g, *P* < 0.01 vs. control group), which further increased in the CHF+*Segatella* group (5.97 ± 0.11 mg/g, *P* < 0.01 vs. CHF group). LCD intervention reduced this ratio to 4.94 ± 0.25 mg/g (*P* < 0.05).

Lung weight-to-body weight ratio (LW/BW): The LW/BW ratio was significantly higher in the CHF+*Segatella* group (10.92 ± 0.46 mg/g, *P* < 0.01 vs. CHF group) compared to the CHF group (9.36 ± 0.65 mg/g). LCD intervention lowered this ratio to 9.56 ± 0.35 mg/g (*P* < 0.05).

Heart Weight-to-Tibia length ratio (HW/TL): The HW/TL ratio was higher in the CHF+*Segatella* group (0.35 ± 0.01 g/cm, *P* < 0.05 vs. CHF group) than in the CHF group (0.33 ± 0.01 g/cm), with a slight decrease observed after LCD intervention (0.32 ± 0.01 g/cm).

Cardiac function assessment

**Fig. 14:**
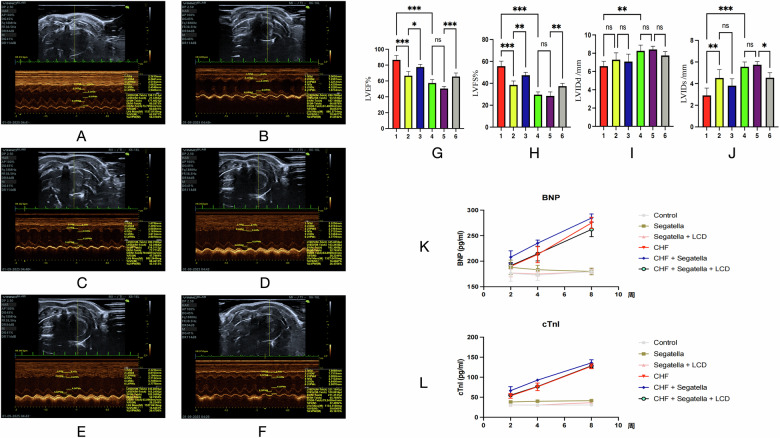
Cardiac function evaluation in rats across different groups. **A**–**F** show echocardiographic images of the hearts of rats in six experimental groups, with IVSd representing interventricular septum thickness during diastole, LVIDd representing left ventricular end-diastolic dimension, LVPWd representing left ventricular posterior wall thickness during diastole, IVSs representing interventricular septum thickness during systole, LVIDs representing left ventricular end-systolic dimension, and LVPWs representing left ventricular posterior wall thickness during systole. These measurements were used to calculate left ventricular ejection fraction (LVEF) and left ventricular fractional shortening (LVFS), among other cardiac function parameters, to assess the impact of different treatments on cardiac function in rats. **G**–**J** show comparisons of key cardiac function indicators across different experimental groups, with the numbers 1–6 on the *x*-axis representing: 1: Control; 2: *Segatella*; 3: *Segatella* + LCD; 4: CHF; 5: CHF + *Segatella*; 6: CHF + *Segatella* + LCD. Data are presented as mean ± standard deviation, with a sample size of six rats per group. Statistical analysis: s indicates no difference, * indicates significance, * indicates *P* < 0.05, ** indicates *P* < 0.01, *** indicates *P* < 0.001. **K** shows the time course of serum brain natriuretic peptide (BNP) levels in rats across different experimental groups; **L** shows the time course of serum cardiac troponin I (cTnI) levels in rats across different experimental groups.

Echocardiography (Fig. 14A–J):

LVEF: The control group had an LVEF of 86.6 ± 5.6%, which decreased to 57.4 ± 4.5% in the CHF group (*P* < 0.01). The CHF+*Segatella* group exhibited a further reduction to 50.4 ± 2.7% (*P* < 0.01 vs. CHF group). LCD intervention increased LVEF to 66.6 ± 5.2% (*P* < 0.01 vs. CHF+*Segatella* group).

LVFS and ventricular dilatation: The CHF+ *Segatella* group had a significantly lower LVFS (28.5 ± 3.7%) compared to the CHF group (29.6 ± 2.5%, *P* < 0.05), with an increased LVIDs of 5.74 ± 0.29 mm (*P* < 0.01 vs. CHF group). LCD intervention significantly improved ventricular dilatation, reducing LVIDs to 4.54 ± 0.47 mm (*P* < 0.01).

Serum biomarkers (Fig. 14K-L):

BNP: The final BNP level in the CHF+*Segatella* group was 284.5 ± 7.97 pg/ml, significantly higher than in the CHF group (274.9 ± 10.95 pg/ml, *P* < 0.05). LCD intervention reduced BNP to 262.2 ± 14.2 pg/ml (*P* < 0.05).

cTnI: The CHF+*Segatella* group had higher cTnI levels (135.8 ± 7.74 pg/ml) compared to the CHF group (127.5 ± 5.88 pg/ml, *P* < 0.05), which slightly decreased to 128.4 ± 5.23 pg/ml after LCD intervention.

Rats with CHF exhibited weight loss, abnormal heart rate, cardiac hypertrophy, and reduced cardiac function (decreased LVEF and ventricular dilatation). *Segatella* exacerbated these pathological changes. LCD intervention improved weight loss, stabilized heart rate and blood pressure, reduced cardiac load, increased LVEF, and decreased injury biomarkers such as BNP, indicating its protective effect on CHF.

### Histopathological changes in cardiac tissue

**Fig. 15:**
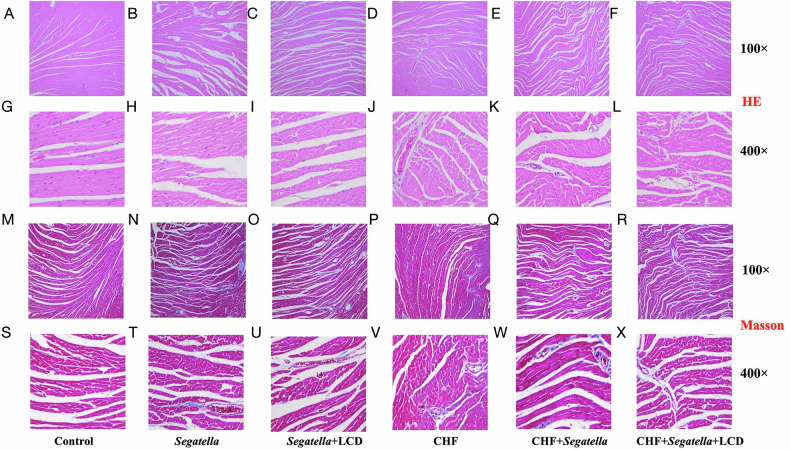
Histopathological examination of cardiac tissue in rats across different groups (HE and Masson staining). **A**–**F** show 100× HE-stained microscopic images of cardiac tissue from each experimental group, while **G**–**L** display 400× images. From left to right, these images depict the HE staining results of cardiac tissue from rats in the following groups: healthy control (Control), *Segatella*, *Segatella*+LCD, chronic heart failure (CHF), CHF + *Segatella*, and CHF + *Segatella*+LCD. These images are used to observe the morphology of cardiomyocytes, the structure of nuclei, and the intercellular tissue relationships, to assess the impact of different treatments on cardiac tissue. **M**–**R** show 100× Masson-stained images of cardiac tissue from each experimental group, and **S**–**X** display 400× images. From left to right, these images depict the Masson staining results of cardiac tissue from rats in the following groups: healthy control (Control), *Segatella*, *Segatella*+LCD, chronic heart failure (CHF), CHF + *Segatella*, and CHF + *Segatella*+LCD. These images are used to observe the distribution and content of collagen fibers in cardiac tissue, to assess the impact of different treatments on the degree of cardiac fibrosis. Collagen fibers appear blue in Masson staining, while cardiomyocytes appear red or light red.

HE Staining (Fig. 15A–L): In the normal control group, cardiomyocytes were arranged orderly without significant inflammatory infiltration (Fig. 15A, G). In the CHF group, cardiomyocytes were hypertrophic and disorganized, with interstitial inflammatory cell aggregation (Fig. 15D, J). These changes were exacerbated in the CHF+*Segatella* group, with evidence of cellular fragmentation and necrosis (Fig. 15E, K). LCD intervention significantly improved these alterations: cardiomyocytes in the CHF+*Segatella* + LCD group were more orderly, with reduced inflammation and necrosis (Fig. 15F, L). In healthy rats, *Segatella* induced only mild cellular loosening, which was alleviated by LCD (Fig. 15B, C, H, I).

Masson’s trichrome staining (Fig. 15M–X): In the normal control group, collagen fibers (blue) were sparsely distributed in a fine network (Fig. 15M, S). In the CHF group, collagen fibers proliferated in bundles, separating cardiomyocytes (Fig. 15P, V). Fibrosis was significantly worsened in the CHF+*Segatella* group, with widespread interstitial filling by collagen fibers (Fig. 15Q, W). LCD intervention thinned collagen fiber bundles and significantly reduced the fibrosis area in the CHF+*Segatella* + LCD group compared to the CHF+*Segatella* group (Fig. 15R, X). In healthy rats, mild fibrosis induced by *Segatella* was inhibited by LCD (Fig. 15N, O, T, U).

### Immunohistochemical expression of apoptosis- and inflammation-related proteins in cardiac tissue

**Fig. 16:**
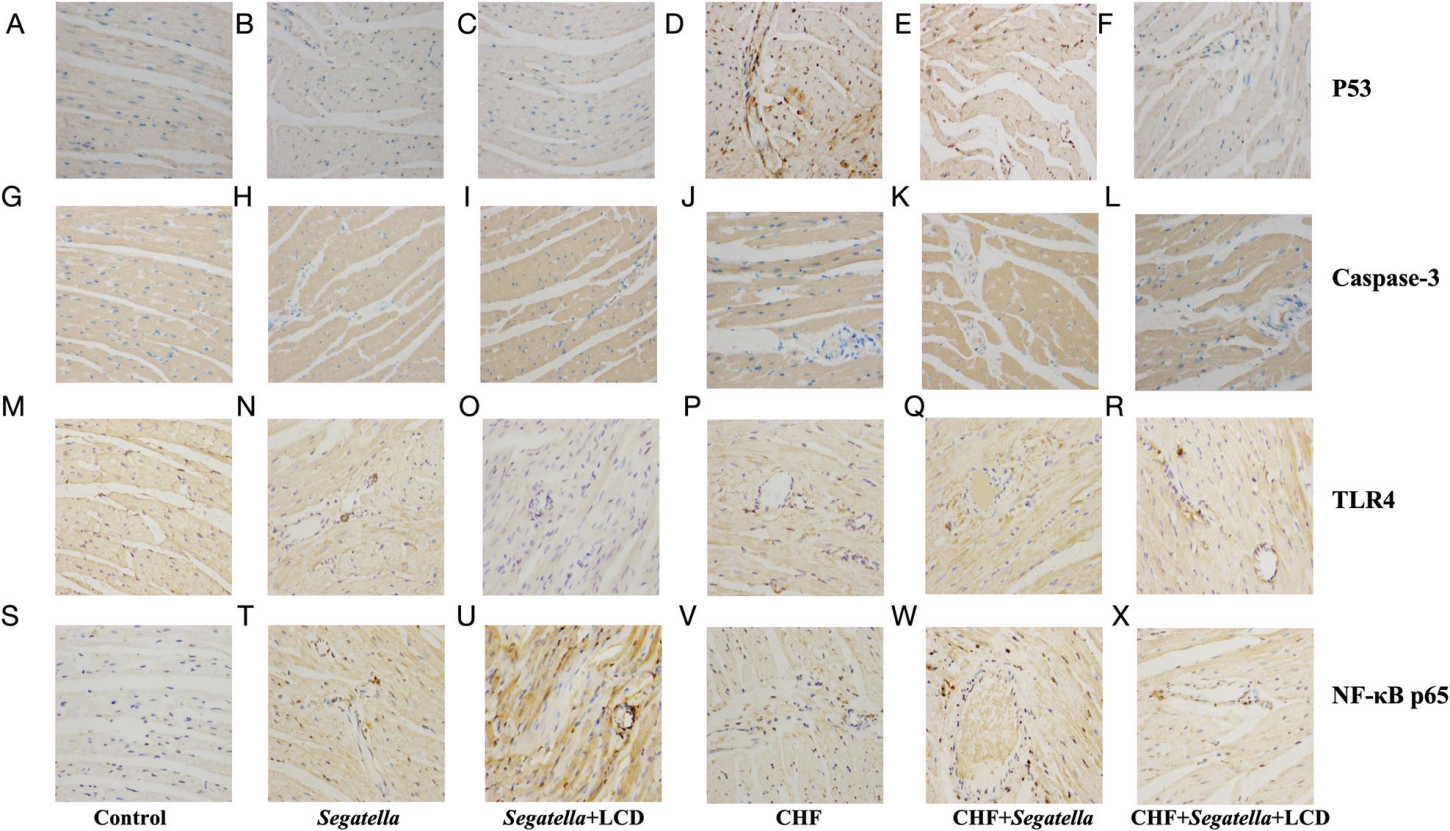
Immunohistochemical staining for p53, caspase-3, TLR4, and NF-κB p65 in cardiac tissue of rats across different experimental groups. The images display the immunohistochemical staining of p53, caspase-3, TLR4, and NF-κB p65 proteins in cardiac tissue from rats in the following groups: healthy control (Control), *Segatella*, *Segatella*+LCD, chronic heart failure (CHF), CHF + *Segatella*, and CHF + *Segatella*+LCD. By examining the staining results in different groups, the impact of various treatments on the expression of these proteins in cardiac tissue can be analyzed. p53: **A**–**F**; caspase-3: **G**–**L**; TLR4: **M**–**R**; NF-κB p65: **S**–**X**. The magnification of the images is 400×.

Immunohistochemical staining results (Fig. 16) revealed distinct expression patterns of p53, cleaved caspase-3, TLR4, and NF-κB p65 in cardiac tissue across different groups of rats. In the normal control group, these proteins were expressed at low levels (Fig. 16A, G, M, S). In healthy rats treated with *Segatella* (*Segatella* group), there was a slight upregulation of protein expression, but the positive signals remained weak (Fig. 16B, H, N, T), a trend that was partially reversed by LCD intervention (*Segatella*+LCD group, Fig. 16C, I, O, U).

In the CHF model group (CHF group), staining for p53 and cleaved caspase-3 was significantly enhanced, indicating increased apoptotic activity; concurrently, expression of TLR4 and NF-κB p65 was markedly elevated, with NF-κB p65 showing nuclear accumulation (Fig. 16D, J, P, V). When CHF rats were co-treated with *Segatella* (CHF + *Segatella* group), positive signals for all proteins were further intensified, with a significant increase in the number of p53- and cleaved caspase-3-positive cells, diffuse high expression of TLR4, and more pronounced nuclear translocation of NF-κB p65 (Fig. 16E, K, Q, W). Notably, LCD intervention significantly attenuated these changes: in the CHF + *Segatella*+LCD group, the intensity and distribution of positive staining for each protein were markedly reduced compared with the CHF + *Segatella* group, and nuclear accumulation of NF-κB p65 was decreased (Fig. 16F, L, R, X).

### Gene and protein expression analysis of the TLR4/MyD88/NF-κB signaling pathway

**Fig. 17:**
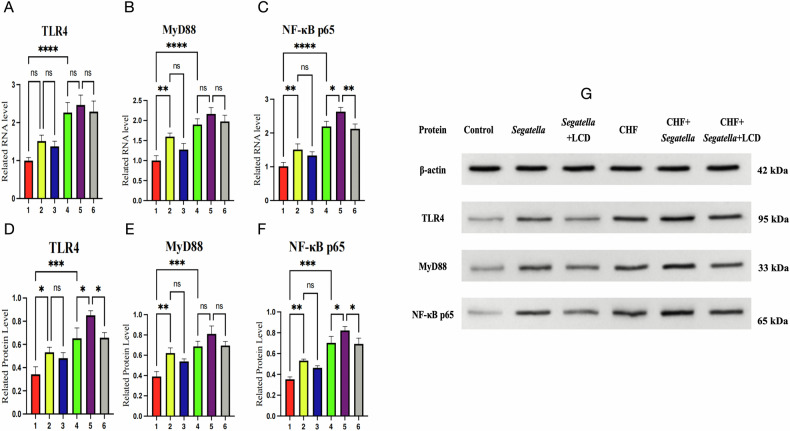
Expression of genes and proteins in the TLR4/MyD88/NF-κB pathway in cardiac tissue of rats across different groups (qPCR and Western blot). **A**–**C** show bar charts of relative RNA levels of TLR4, MyD88, and NF-κB p65 in cardiac tissue from different groups. **D**–**F** present bar charts of relative protein levels of TLR4, MyD88, and NF-κB p65 in cardiac tissue from different treatment groups (WB), and **G** shows the corresponding Western blot bands (β-actin as the internal reference). The numbers 1–6 on the *x*-axis represent: 1: Control; 2: *Segatella*; 3: *Segatella*+LCD; 4: CHF; 5: CHF + *Segatella*; 6: CHF + *Segatella*+LCD. Sample size was six rats per group. Statistical analysis: s indicates no difference, * indicates significance, * indicates *P* < 0.05, ** indicates *P* < 0.01, *** indicates *P* < 0.001.

qPCR and WB results (Fig. 17) showed consistent expression trends of TLR4, MyD88, and NF-κB p65 at both the gene and protein levels, with significant differences among groups.

Gene level (Fig. 17A–C): In the normal control group, mRNA levels of TLR4, MyD88, and NF-κB p65 were maintained at a low baseline. The *Segatella* group showed a slight increase compared to the control group, but without statistical significance. The CHF group exhibited significant upregulation of these three genes (*P* < 0.01). The CHF+*Segatella* group had a further surge in mRNA levels (approximately 1.5-fold increase in TLR4 and NF-κB p65 compared to the CHF group, *P* < 0.001). LCD intervention significantly suppressed this overexpression, reducing mRNA levels by 30–40% in the CHF+*Segatella* + LCD group compared to the CHF+*Segatella* group (*P* < 0.01).

Protein level (Fig. 17D–G): WB results were consistent with qPCR. In the normal control group, protein expression levels of TLR4, MyD88, and nuclear NF-κB p65 were extremely low. The CHF group had a significant increase compared to the control group (*P* < 0.01). The CHF+*Segatella* group reached a peak in protein expression (approximately twofold increase in NF-κB p65 compared to the CHF group, *P* < 0.001). LCD intervention significantly reduced protein levels in the CHF+*Segatella*+LCD group compared to the CHF+*Segatella* group (*P* < 0.01), with particularly notable downregulation of TLR4 and NF-κB p65.

### Analysis of gut microbiota structure and diversity in rats

**Fig. 18:**
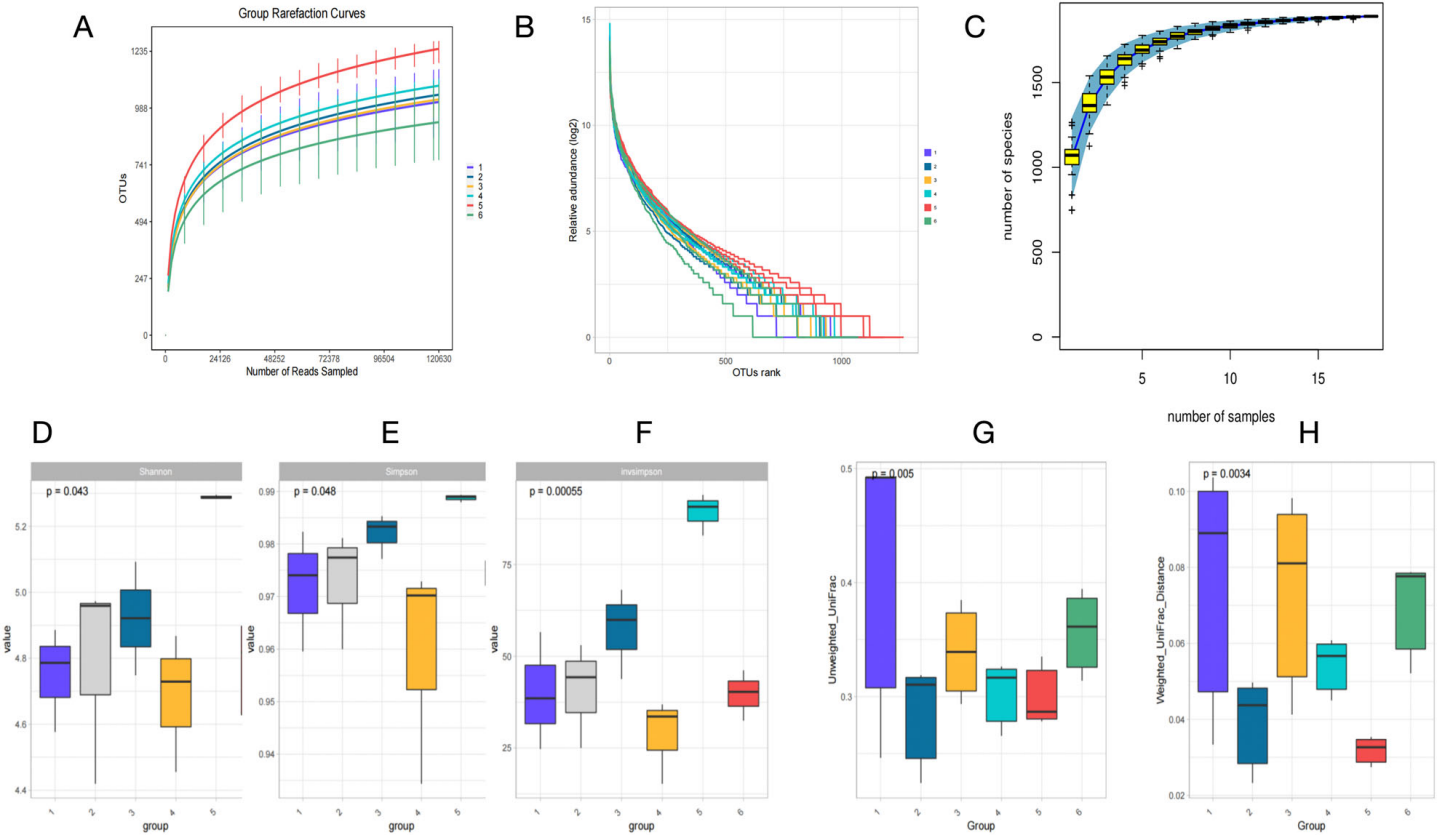
Analysis of gut microbiota diversity in rats across different groups (16S rRNA sequencing). **A** Alpha-diversity rarefaction curves; **B** Rank abundance curves; **C** Species accumulation curves; Intergroup differences in alpha-diversity, **D** Shannon index, **E** Simpson index, **F** Inverse Simpson index; Analysis of gut microbiota differences based on Unifrac distance, **G** Results based on unweighted Unifrac distance, **H** Results based on weighted Unifrac distance. The numbers 1–6 on the *x*-axis represent: 1: Control; 2: *Segatella*; 3: *Segatella*+LCD; 4: CHF; 5: CHF + *Segatella*; 6: CHF + *Segatella*+LCD. Sample size was six rats per group.

Sequencing quality assessment indicated that the rarefaction curves (Fig. 18A) plateaued and the species accumulation curves (Fig. 18C) approached saturation, suggesting that the sequencing depth and sample size were sufficient to cover the main diversity of the gut microbiota in each group. The rank abundance curve (Fig. 18B) showed that the CHF+*Segatella* group had the lowest evenness of species distribution, indicating the most significant imbalance in microbial community structure.

Alpha diversity analysis (Fig. 18D–F): Compared with the normal control group, the CHF group exhibited a significant decrease in the Shannon index (reflecting species diversity, *P* < 0.05) and an increase in the Simpson index (reflecting the concentration of dominant species, *P* < 0.05). The Shannon index in the CHF+*Segatella* group further decreased by 21.3% compared to the CHF group (*P* < 0.01), while the Simpson index reached the highest level (*P* < 0.01). LCD intervention partially reversed this trend, with the Shannon index in the CHF+*Segatella*+LCD group increasing by 15.7% compared to the CHF+*Segatella* group (*P* < 0.05) and the Simpson index decreasing (*P* < 0.05).

Beta diversity analysis (based on Unifrac distance, Fig. 18G, H): Both unweighted (reflecting the presence/absence of species) and weighted (reflecting differences in species abundance) analyses indicated that the microbial community structure of the CHF+*Segatella* group was significantly separated from that of the normal control group (*P* = 0.0034 and *P* = 0.005). After LCD intervention, the microbial community structure of the CHF+*Segatella*+LCD group shifted towards that of the normal control group, with a reduced separation from the CHF+*Segatella* group (*P* < 0.05).

**Fig. 19:**
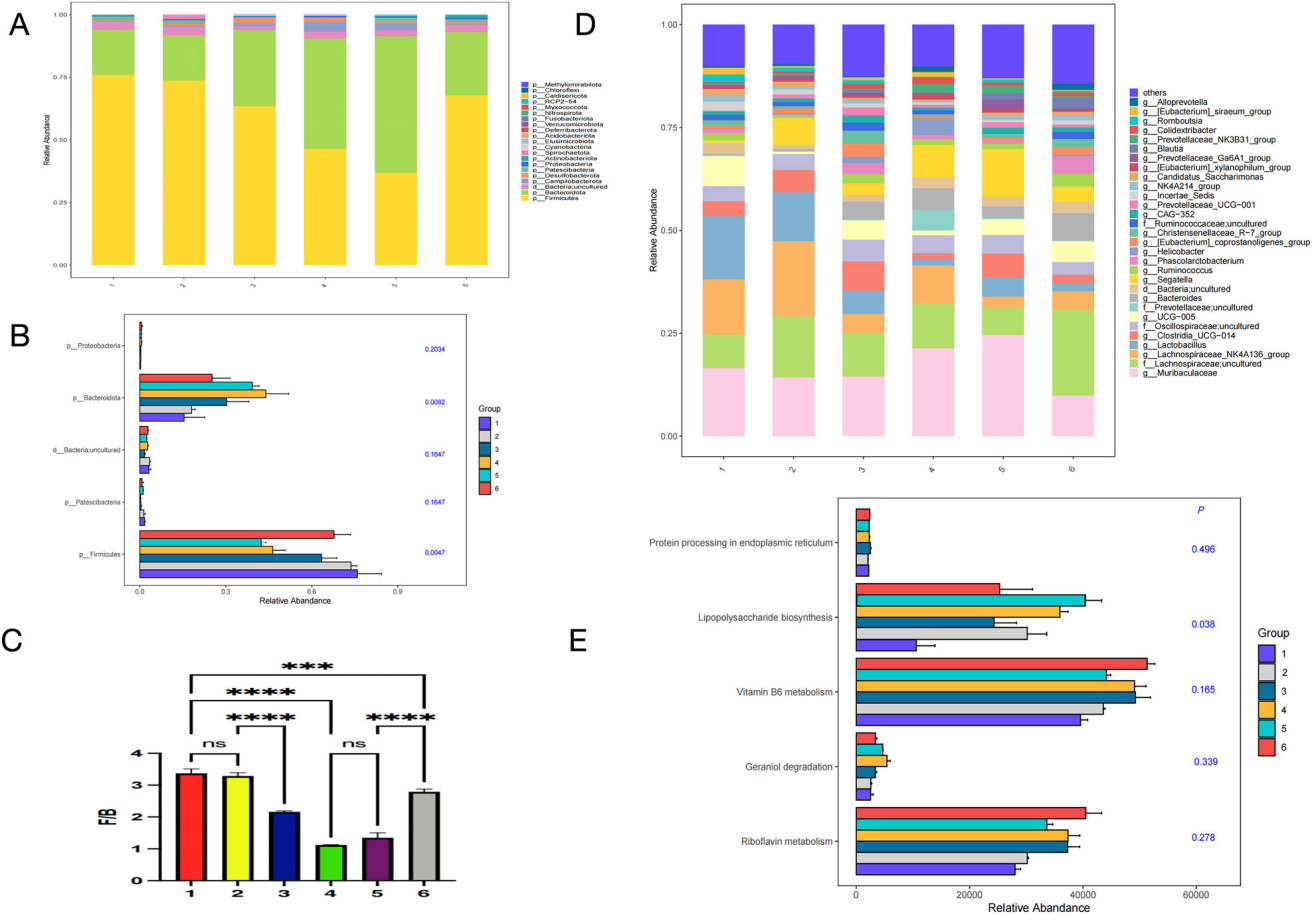
Composition and functional prediction of gut microbiota in rats across different groups (16S rRNA sequencing). **A** Relative abundance of gut microbiota at the phylum level in different treatment groups, with Firmicutes and Bacteroidota as dominant phyla, used to compare the effects of different treatments on their relative abundance. **B** Results of Kruskal–Wallis test, which was used to determine whether there were significant differences in the relative abundance of gut microbiota among different treatment groups, supporting the analysis of changes in phylum abundance across groups. **C** Changes in the F/B ratio across different treatment groups. The F/B ratio decreased in the CHF model group and increased in the CHF + *Segatella*+LCD group but remained different from the healthy group, reflecting the impact of different treatments on gut microbiota structure and the ameliorative effect of LCD. **D** Relative abundance of gut microbiota at the genus level in different treatment groups. **E** Functional gene prediction and metabolic pathway difference analysis of gut microbiota in different treatment groups. The numbers 1–6 on the *x*-axis represent: 1: Control; 2: *Segatella*; 3: *Segatella*+LCD; 4: CHF; 5: CHF + *Segatella*; 6: CHF + *Segatella*+LCD. Sample size was six rats per group. Statistical analysis: s indicates no difference, * indicates significance, * indicates *P* < 0.05, ** indicates *P* < 0.01, *** indicates *P* < 0.001.

#### Phylum-level analysis (Fig. 19A-B)

Compared with the healthy control group, the relative abundance of Firmicutes was significantly reduced, while that of Bacteroidota was significantly increased in the CHF model group. The ratio of Firmicutes to Bacteroidota (F/B value) is often regarded as a marker of gut microbiota imbalance, especially in chronic disease states. In the *Segatella* and *Segatella*+LCD groups, the relative abundance of Firmicutes was similar to that of the healthy control group. However, in the CHF + *Segatella* group, the relative abundance of Firmicutes further decreased. The species composition in the CHF + *Segatella*+LCD group was closer to that of the healthy control group. Moreover, Kruskal-Wallis test results showed significant differences in microbial relative abundance among different groups, which further supported the above findings. Notably, the F/B value also varied among different groups. In the CHF model group, the F/B value significantly decreased, reflecting a reduction in Firmicutes and an increase in Bacteroidota. In the CHF + *Segatella*+LCD group, the F/B value increased but remained significantly different from that of the healthy control group.

#### Genus-level analysis (Fig. 19D)

In the healthy control group, genera such as Muribaculaceae had relatively high abundance, maintaining a stable gut microbiota. In the *Segatella* group, the abundance of *Segatella* increased, indicating that gavage altered the microbial structure. In the *Segatella*+LCD group, the microbial structure further changed due to the addition of the LCD diet, suggesting a combined effect of LCD and gavage on the gut microbiota. Compared with the healthy control group, the abundance of many genera was significantly different in the CHF group, revealing that CHF causes gut microbiota imbalance. In the CHF + *Segatella* group, in addition to the increase in *Segatella*, other genera also changed, and the effects were different from those of gavage in the healthy state, implying that the CHF state affected the gut’s response to gavage. In the CHF + *Segatella* + LCD group, the abundance of some genera approached that of the healthy control group.

#### Functional prediction (Fig. 19E)

The main biological functions associated with the sequenced microbiota in this study involve protein processing in the endoplasmic reticulum, lipopolysaccharide biosynthesis, vitamin B6 metabolism, geraniol degradation, and riboflavin metabolism, among others. Analysis revealed that only the lipopolysaccharide biosynthesis pathway had a *P* value of 0.038, indicating significant differences among different treatment groups. This suggests that different treatments significantly altered the lipopolysaccharide synthesis of Gram-negative bacteria in the gut microbiota. Therefore, the impact of different treatments on gut microbial metabolic functions varies, with the lipopolysaccharide biosynthesis pathway being particularly affected, while differences in other pathways were not significant.

Serum microbiota metabolites and inflammatory cytokine levels in cardiac tissue

**Fig. 20:**
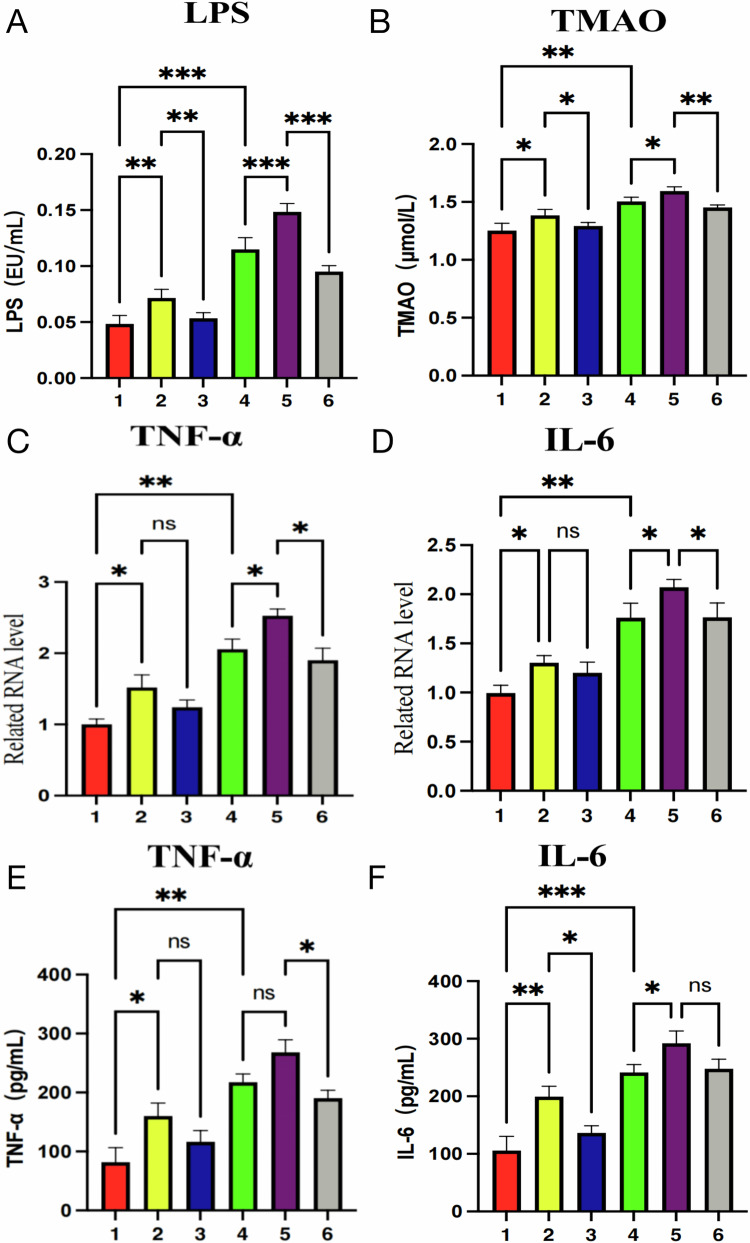
Levels of serum microbiota metabolites (LPS, TMAO) and cardiac tissue inflammatory cytokines (TNF-α, IL-6) in rats across different groups. **A**, **B** Comparison of serum LPS and TMAO concentrations in different treatment groups. Expression of cardiac tissue inflammatory cytokines TNF-α and IL-6, with **C**, **D** showing gene expression (qPCR) and **E**, **F** showing protein expression (ELISA). The numbers 1–6 on the *x*-axis represent: 1: Control; 2: *Segatella*; 3: *Segatella* + LCD; 4: CHF; 5: CHF + *Segatella*; 6: CHF + *Segatella* + LCD. Sample size was six rats per group. Statistical analysis: s indicates no difference, * indicates significance, * indicates *P* < 0.05, ** indicates *P* < 0.01, *** indicates *P* < 0.001.

1. Changes in microbiota metabolites LPS and TMAO (Fig. 20A, B)

Serum analysis revealed significant differences in lipopolysaccharide (LPS) and trimethylamine N-oxide (TMAO) levels across groups. The normal control group maintained low levels of LPS and TMAO. The *Segatella* group exhibited elevated levels of both metabolites, and the CHF group showed a significant increase compared to the control group (*P* < 0.01). The CHF+*Segatella* group had even higher levels. LCD intervention significantly reduced LPS and TMAO levels.

2. Expression of TNF-α and IL-6 in cardiac tissue (Fig. 20C–F)

Consistent trends in the expression of inflammatory cytokines TNF-α and IL-6 were observed at both the gene and protein levels.

qPCR Results (Fig. 20C, D): CHF pathology was associated with significantly elevated gene expression levels of TNF-α and IL-6 (both *P* < 0.01 vs. healthy controls), indicating that uncontrolled inflammation is a key mechanism in CHF progression. *Segatella* administration in healthy conditions did not significantly alter cytokine expression (ns), but in the CHF model, it exacerbated inflammation: TNF-α and IL-6 expression further increased in the CHF+*Segatella* group (both *P* < 0.05), revealing that the bacterium amplifies pathological damage by activating inflammatory signaling pathways. LCD intervention significantly reduced TNF-α and IL-6 expression in the CHF+*Segatella*+LCD group compared to the CHF+*Segatella* group (both *P* < 0.05), demonstrating that LCD suppresses cytokine synthesis by modulating the microbiota–metabolite axis. However, expression levels in these groups remained higher than in healthy controls.

ELISA results (Fig. 20E, F): Healthy controls exhibited low expression of TNF-α and IL-6, reflecting normal cardiac inflammatory homeostasis. Regarding intervention effects, *Segatella*’s pro-inflammatory impact was confirmed by significantly higher protein expression of TNF-α and IL-6 in the *Segatella* group compared to healthy controls (both *P* < 0.05), indicating that the bacterium activates myocardial inflammatory pathways directly or indirectly. The synergistic worsening of CHF and dysbiosis was evident as TNF-α expression surged in the CHF group, with further elevation of IL-6 in the CHF+*Segatella* group, demonstrating an inflammatory cascade amplification effect due to microbiota imbalance and heart failure. Lastly, the protective mechanism of LCD was highlighted by a significant reduction in TNF-α in the CHF+*Segatella*+LCD group compared to the *Segatella*+LCD group (*P* < 0.05), with a downward trend in IL-6 levels.

## Discussion

CHF, as the terminal stage of cardiovascular disease, is far more complex in its pathogenesis than traditionally recognized. In recent years, the emergence of the “gut-heart axis” theory has shattered the anatomical boundaries between the heart and gut, revealing a new paradigm in which the gut microbiota regulates cardiac function through multiple pathways, including metabolic, immune, and neural mechanisms [15]. This study, through a three-tiered validation involving clinical cohorts, cellular models, and animal experiments, has for the first time systematically elucidated that *Segatella*, as a key driver of CHF progression, exacerbates myocardial injury via the TLR4/NF-κB pathway. Furthermore, we have demonstrated that a LCD can exert protective effects by targeting this pathway. This finding not only adds a new critical node to the “microbiota-immune-metabolic” interaction network in CHF but also provides translatable experimental evidence in the field of non-pharmacological interventions.

### *Segatella* as a key mediator of myocardial injury in CHF

Our study reveals that *Segatella*, a previously underappreciated contributor, significantly impacts the pathogenesis of CHF. In human cohorts, CHF patients exhibit markedly elevated *Segatella* abundance, correlating with impaired cardiac function (reduced LVEF and increased NT-proBNP). This aligns with emerging evidence that gut-derived pathogens exacerbate heart failure by amplifying systemic inflammation and cardiac remodeling [16]. For instance, *Enterococcus* and *Escherichia* species, known to produce pro-inflammatory LPS, worsen ventricular dysfunction by activating TLR4 [17, 18]. However, *Segatella* (a member of the *Prevotellaceae* family) had not been previously linked to CHF [19]. Our in vitro experiments demonstrate that *Segatella* dose-dependently exacerbates doxorubicin-induced apoptosis and oxidative stress in cardiomyocytes. In vivo data further indicate accelerated cardiac fibrosis and inflammation in CHF rats colonized with *Segatella*. These findings suggest that *Segatella* may act synergistically with traditional cardiac stressors, such as ischemia and neurohormonal activation, to amplify myocardial injury.

#### The detrimental effects of *Segatella* appear to be mediated through several interrelated pathways

Activation of the TLR4/NF-κB Axis: Colonization with *Segatella* may increase intestinal permeability (“leaky gut”), facilitating the translocation of microbial products such as LPS into the circulation [20, 21]. LPS binding to TLR4 on cardiomyocytes and macrophages triggers NF-κB activation. This cascade upregulates pro-inflammatory cytokines (e.g., TNF-α, IL-6) and promotes cardiomyocyte apoptosis via caspase-3 cleavage [22]. Our Western blot data confirm elevated levels of cleaved caspase-3 and p53 in *Segatella*-treated groups, consistent with TLR4-mediated apoptotic signaling [23, 24].

Metabolite-mediated toxicity: Metabolomic analysis reveals that *Segatella* colonization is associated with increased levels of indole derivatives and short-chain ketones. Indole, a tryptophan metabolite produced by gut bacteria, has been shown to induce endothelial dysfunction and oxidative stress via aryl hydrocarbon receptor (AhR) activation [25]. Similarly, dysregulated ketone metabolism may impair mitochondrial energy metabolism, exacerbating the characteristic energy deficit in the failing heart [26, 27].

Fibrosis signaling: Animal experiments demonstrate that *Segatella* intervention significantly exacerbates myocardial fibrosis in CHF rats. Mechanistically, *Segatella* may induce the transformation of fibroblasts into myofibroblasts via TLR4/NF-κB activation, while its metabolites may directly activate the TGF-β1 receptor. TGF-β1, a key regulator of fibrosis, is known to synergize with TLR4 signaling to promote fibroblast activation and extracellular matrix remodeling [28, 29]. This is consistent with prior studies showing that gut microbiota dysbiosis can exacerbate cardiac fibrosis through serotonin and bacterial metabolites of gut origin [30].

#### Comparison with existing studies

Our study provides novel insights into the role of *Segatella* in CHF progression, highlighting its unique mechanisms compared to other well-known pro-inflammatory genera such as Escherichia. While *Escherichia* species are known to produce LPS that activates the TLR4/NF-κB pathway, leading to systemic inflammation and cardiac dysfunction [17, 18], *Segatella* appears to have distinct effects. *Segatella* colonization is associated with increased levels of indole derivatives and short-chain ketone acids, which may contribute to myocardial injury through different pathways [25–27]. This suggests that *Segatella* may exacerbate CHF through a combination of direct toxic effects and modulation of host inflammatory responses, distinct from the LPS-mediated inflammation seen with *Escherichia*.

### Low-carbohydrate diet (LCD) intervention: linking gut microbiota modulation and cardiac protection

As a potential intervention for CHF, LCD has garnered increasing attention in recent years [31]. Our study demonstrates that LCD exerts protective effects against CHF in both cellular and animal models. In vitro, LCD inhibits *Segatella*-induced cardiomyocyte apoptosis and oxidative stress, modulates the expression of related genes and proteins, and attenuates inflammatory responses. In vivo, LCD intervention improves cardiac function in CHF rats, reduces myocardial fibrosis and inflammation, and alleviates cardiac hypertrophy and pulmonary edema. The mechanisms underlying LCD’s protective effects on CHF are likely multifaceted.

Firstly, LCD can modulate the structure and function of the gut microbiota [32]. Studies have shown that the carbohydrate content in the diet significantly influences gut microbiota composition [33, 34]. By reducing carbohydrate intake, LCD may alter the gut’s nutritional environment, promoting the growth of beneficial bacteria and inhibiting the proliferation of harmful bacteria, thereby improving gut microbial balance [32]. For instance, LCD may increase the abundance of SCFA-producing beneficial bacteria in the gut, raising SCFA levels [35]. SCFAs can exert cardioprotective effects through various pathways, such as activating G protein-coupled receptors (GPCRs), modulating immune cell function, and suppressing inflammatory responses; they can also promote the expression of tight junction proteins in intestinal epithelial cells, enhancing gut barrier function and reducing the translocation of bacterial endotoxins and inflammatory factors into the bloodstream, thereby alleviating cardiac burden [36, 37].

Moreover, LCD may also influence CHF by modulating metabolic pathways. For example, LCD may regulate pathways such as carbohydrate metabolism, lipid metabolism, and amino acid metabolism, improving energy supply and metabolic efficiency in cardiomyocytes [38]. Specifically, LCD may promote fatty acid β-oxidation, increase cardiomyocyte utilization of fatty acids, reduce anaerobic glycolysis of glucose, and enhance energy production efficiency in cardiomyocytes. Concurrently, LCD may modulate amino acid metabolism, providing essential nutrients and metabolic precursors for cardiomyocytes, promoting their repair and regeneration [38, 39]. However, our study also found that LCD did not fully normalize CHF-related indicators, suggesting certain limitations in its intervention effects. This may be related to factors such as the dose, duration, and formulation of LCD intervention. Future research needs to further optimize LCD intervention protocols and explore its mechanisms of action to enhance its therapeutic efficacy for CHF.

#### Long-term safety of LCD interventions

The long-term safety and efficacy of LCD interventions in CHF management are critical considerations for clinical translation. Our study demonstrates significant improvements in cardiac function and reductions in myocardial fibrosis and inflammation within an 8-week period. However, the long-term effects of LCD beyond this period require further investigation. Longitudinal studies and clinical trials are needed to assess the sustained benefits and potential risks of prolonged LCD use. Future research should focus on evaluating the impact of LCD on metabolic profiles, gut microbiota stability, and overall cardiovascular health over extended periods. Additionally, addressing potential side effects and ensuring patient adherence to dietary interventions will be essential for developing sustainable treatment strategies.

### Strengths and limitations

Compared with previous studies on the relationship between CHF and the gut microbiota, our study has the following innovations. First, this study employed a multidisciplinary approach, integrating population research, cellular experiments, and animal experiments to comprehensively and systematically explore the relationship between the gut microbiota and CHF. This multi-dimensional research method helps to enhance the reliability and persuasiveness of the study results, providing a more comprehensive perspective for understanding the pathogenesis of CHF. Second, this study focused on the role of the specific bacterial genus *Segatella* in CHF, revealing its mechanisms of myocardial cell injury and the intervention effects of LCD through a series of experiments. Previous studies have mostly focused on overall changes in the gut microbiota, with relatively few studies on the mechanisms of specific bacterial genera in CHF. The findings of this study provide new targets and directions for the etiological research of CHF. In addition, while investigating the relationship between the gut microbiota and CHF, this study also explored the changes in serum metabolites and their associations with the gut microbiota and myocardial injury, as well as the mechanisms of action of LCD intervention on gut microbial metabolites and cardiac function. This multi-omics integrated analysis approach helps to more comprehensively reveal the pathophysiological mechanisms of CHF and provides richer information for the development of new diagnostic and therapeutic strategies.

Despite the achievements of this study, there are still some limitations. First, in terms of study samples, the sample size included in this study is relatively limited, especially in cellular and animal experiments. Although stratified sampling was carried out in the population study, the limitation of sample size may affect the statistical power and generalizability of the study results. Future studies need to increase the sample size and include more CHF patients from different regions, ethnicities, and disease severities to enhance the universality and reliability of the study results. Second, in the animal experiments, only short-term intervention effects were observed, and there is still a lack of sufficient understanding of the long-term intervention effects and safety of LCD. Future studies need to conduct long-term animal experiments and clinical trials to comprehensively evaluate the long-term efficacy and safety of LCD in the treatment of CHF.

## Conclusion

In summary, this study, through clinical cohorts, cellular, and animal experiments, has demonstrated that the abundance of *Segatella* in the gut of CHF patients is significantly increased and closely related to the deterioration of cardiac function and metabolic disorders. This bacterium exacerbates cardiomyocyte apoptosis, oxidative stress, and inflammatory responses in a dose-dependent manner by activating the TLR4/NF-κB pathway, further worsening cardiac function and promoting myocardial fibrosis in animal models. Conversely, a low-carbohydrate diet (LCD) can reduce the abundance of *Segatella*, improve gut microbiota structure, and decrease the production of LPS and TMAO, thereby attenuating inflammation and myocardial injury by inhibiting TLR4/NF-κB pathway activity, ultimately improving cardiac function in CHF rats. Collectively, *Segatella* drives the progression of CHF via the TLR4/NF-κB pathway, and LCD can target this bacterium and pathway to exert protective effects, providing new targets and non-pharmacological intervention strategies for the precise diagnosis and treatment of CHF.

## Methods

### Study design and participant recruitment

Consecutive patients hospitalized for CHF were enrolled between February and August 2024 at the Heart Failure Unit of the First Affiliated Hospital of Xinjiang Medical University. CHF was diagnosed according to the 2023 Chinese Guidelines for the Diagnosis and Treatment of Heart Failure [40], the 2021 ESC Guidelines for the Diagnosis and Treatment of Acute and Chronic Heart Failure [41], and the 2022 ACC/AHA/Heart Failure Society of America Guideline for the Management of Heart Failure [42]. Eligible patients were required to have at least two documented HF episodes separated by ≥6 months (detailed inclusion and exclusion criteria are provided in Supplementary Methods). Both stable CHF outpatients and those admitted with acute decompensation were recruited. To enhance generalizability, we excluded individuals who (1) presented in critical condition (cardiogenic shock or need for mechanical ventilation), (2) had severe infection or any disorder likely to substantially alter the gut microbiota, or (3) received antibiotics or probiotics within the preceding 3 months.

Controls were recruited from the same institution (First Affiliated Hospital of Xinjiang Medical University) and had no documented cardiovascular disease. Each potential control underwent comprehensive evaluation comprising detailed medical history, physical examination, transthoracic echocardiography, 12-lead electrocardiography, and 24-h Holter monitoring. The absence of CHF was confirmed by consensus of at least two attending physicians who integrated clinical symptoms, signs, and laboratory parameters (complete blood count, comprehensive metabolic panel, inflammatory biomarkers, coagulation profile, thyroid function tests, etc.) with electrocardiographic findings. To ensure baseline comparability, controls were frequency-matched to cases on age, sex, and functional class.

A total of 257 participants—152 patients with CHF and 105 healthy controls—were ultimately enrolled. From this primary cohort, a stratified random subsample was generated according to age, sex, and NYHA functional class; 50 CHF patients and 50 controls were then selected proportionally from each stratum for metagenomic and taxonomic analyses. The study protocol was approved by the Ethics Committee of the First Affiliated Hospital of Xinjiang Medical University (approval no. K202402-05), registered at the Chinese Clinical Trial Registry (ChiCTR1900027476), and written informed consent was obtained from all participants prior to enrollment.

### Clinical data and sample collection

Comprehensive baseline characteristics—including sex, age, height, weight (for BMI calculation), admission blood pressure, medical history, comorbidities, lifestyle factors, prior illnesses, and current medications—were systematically documented.

After a 12-h fast, peripheral venous blood was collected for complete blood count, comprehensive metabolic panel, inflammatory biomarkers, coagulation profile, thyroid function tests, and NT-proBNP quantification. Concurrently, 12-lead electrocardiography, transthoracic echocardiography, coronary angiography, and carotid ultrasonography were performed and recorded.

For stool collection, participants received dedicated sampling kits along with on-site demonstrations and written instructions to ensure standardized collection (avoid urine contamination, sample multiple stool regions, immediate sealing, etc.). Each stool specimen was divided into five aliquots of 200 mg, snap-frozen in liquid nitrogen, and stored at −80 °C until further processing (see Supplementary Methods for details).

### Metagenomic sequencing

Fecal microbiota were profiled by whole-genome shotgun metagenomics encompassing DNA extraction, library construction, cluster generation, sequencing, and downstream bioinformatics. Briefly, microbial DNA was extracted from 200 mg stool aliquots using the QIAamp DNA Stool Mini Kit (Qiagen) and mechanically sheared to the desired fragment size. Libraries were prepared according to Illumina protocols and sequenced on the HiSeq platform in paired-end mode (2 × 150 bp); raw reads were deposited as FASTQ files. Detailed laboratory procedures are provided in Supplementary Methods.

### Taxonomic and functional annotation

Clean reads were aligned against the NCBI reference database of human gut microbial genomes using SOAPaligner for taxonomic assignment. To generate functional profiles, non-redundant gene catalogs were queried against the KEGG genes database via BLAST; KO (KEGG Orthology) annotations were then assigned, and gene abundances were summed to construct KO abundance tables. Detailed analytical workflows are provided in Supplementary Methods.

### Untargeted metabolomics analysis

The untargeted metabolomics workflow comprised serum sample preparation, quality control (QC) sample generation, LC–MS/MS acquisition, and subsequent data processing. For sample preparation, proteins were precipitated with a methanol–acetonitrile mixture to maximize metabolite recovery. Metabolite separation and detection were performed on an Agilent 1290 Infinity LC system coupled to an AB SCIEX TripleTOF 5600/6600 mass spectrometer. Samples were randomized prior to analysis, and QC aliquots were interspersed throughout the run to monitor system stability. Detailed protocols are provided in the Supplementary Methods.

### Data processing and analysis

After quality control, metagenomic reads were taxonomically classified using SOAPaligner and functionally annotated via BLAST against the KEGG database. Metabolomic features were identified with our in-house Met DDA and Lip DDA workflows. After univariate statistical analysis, we applied the Benjamini-Hochberg procedure to control the false discovery rate (FDR) at a significance level of 0.05. This correction was specifically applied to the results of the untargeted metabolomics analysis to adjust for multiple comparisons and ensure the robustness of the identified significant metabolites. Microbial α-diversity (Shannon, Simpson, Pielou indices) and β-diversity (Jensen–Shannon distance) were calculated to characterize community structure, and differential taxa were determined using LEfSe.

For untargeted metabolomics, an OPLS-DA model was constructed; variables with a variable importance in projection (VIP) score >1 were retained as candidate discriminatory metabolites. Their significance was subsequently confirmed by univariate statistics (VIP > 1 and *P* < 0.05). Spearman’s rank correlation was used to assess associations between metabolites and clinical variables.

To evaluate the robustness of candidate metabolites and visualize inter-sample relationships as well as metabolite expression patterns, hierarchical clustering of significantly altered metabolites was performed. Additionally, the mean decrease in accuracy from random forest analysis was employed to rank metabolite importance. KEGG pathway mapping was utilized to interpret gene functions at the systems level.

### Diagnostic model development

Following the above analyses, we constructed multiple diagnostic models: (i) gut-microbiota-only, (ii) microbiota combined with clinical parameters, (iii) differential -metabolite-only, and (iv) metabolite-plus-clinical composite models. The discriminative performance of CHF-related variables was assessed by receiver operating characteristic (ROC) curve analysis, with the area under the curve (AUC) serving as the primary metric of predictive accuracy. Multivariable logistic regression was employed to identify independent risk factors for CHF. Within the modeling framework, a stringent stability selection procedure—re-evaluating AUCs across up to 100 bootstrap iterations—was applied to confirm the robustness of the biomarker signatures.

**Fig. 21:**
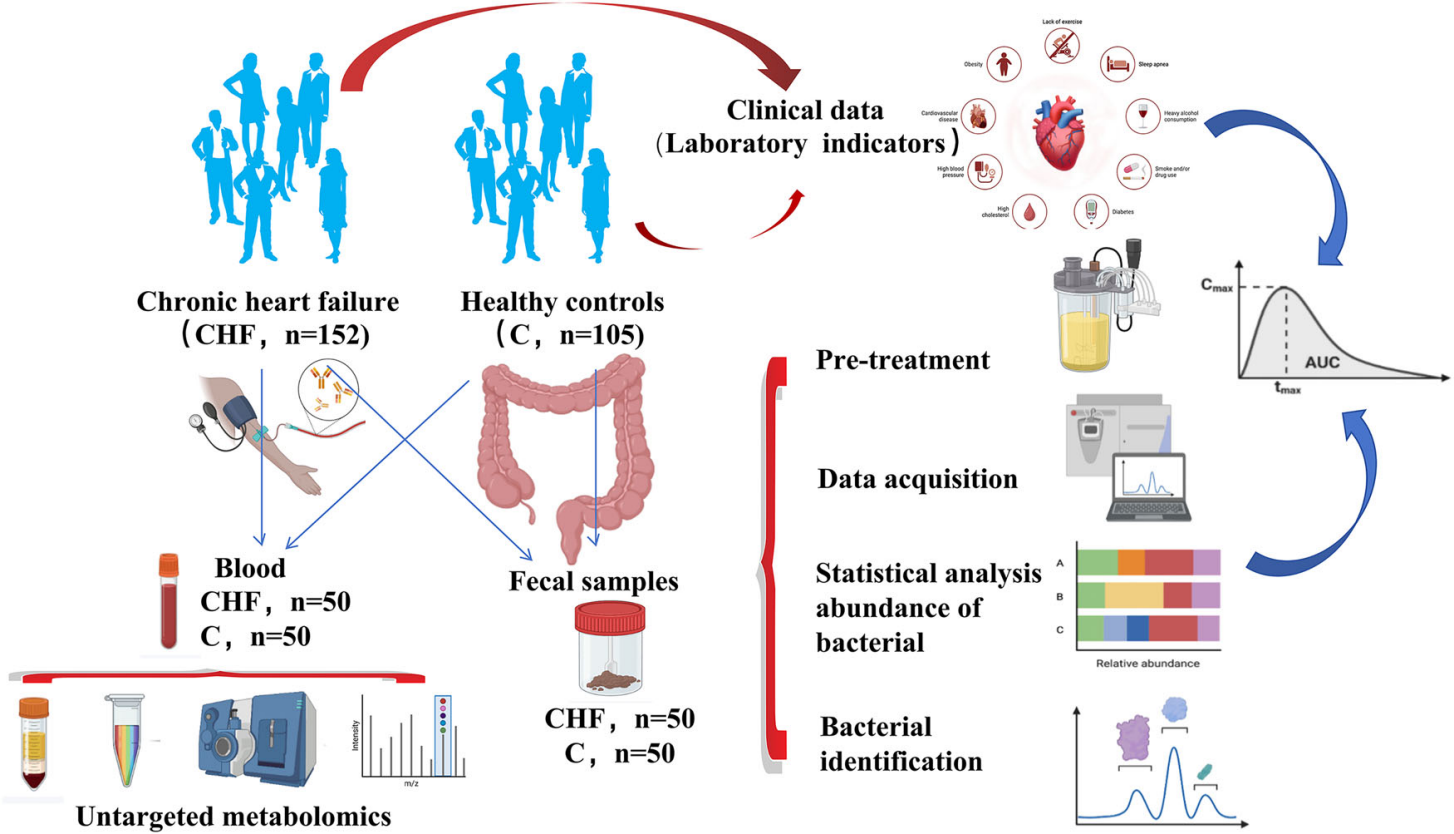
Study workflow. C control group, CHF chronic heart failure group.

The overall workflow of the human study is depicted in Fig. 21.

### Ex vivo studies

To elucidate the mechanisms by which *Segatella* induces cardiomyocyte injury and to evaluate the protective effects of a LCD, we employed rat H9c2 cardiomyocytes. After establishing a doxorubicin-based myocardial injury model, cells were subjected to various treatments, and subsequent analyses assessed apoptosis, reactive oxygen species (ROS) levels, gene expression, protein expression, and inflammatory cytokine release.

### Materials and cell culture

Cells: H9c2 rat cardiomyoblasts (Procell, Wuhan, China).

Bacteria: *Segatella* strain (BeNa Culture Collection, Beijing, China).

Media: standard DMEM and low-glucose DMEM (for LCD intervention).

Myocardial injury inducer: doxorubicin (Dox, 1 µM, 24 h).

Incubation: 37 °C, 5% CO₂ humidified incubator.

**Experimental groups**

- Control: H9c2 cells maintained under standard conditions without any treatment, serving as the baseline.
- Dox: Cells exposed to 1 µM doxorubicin for 24 h to establish the injury model.
- Dox + *Segatella*-L: After doxorubicin exposure, cells were co-incubated with *Segatella* at a multiplicity of infection (MOI) of 0.1 for 24 h.
- Dox + *Segatella*-H: After doxorubicin exposure, cells were co-incubated with *Segatella* at MOI = 1 for 24 h.
- Dox + *Segatella*-L+LCD: Following doxorubicin exposure, cells received *Segatella* at MOI = 0.1 and were simultaneously switched to 20% low-glucose DMEM (LCD) for 24 h.
- Dox + *Segatella*-H+LCD: Following doxorubicin exposure, cells received *Segatella* at MOI = 1 and were simultaneously switched to 20% low-glucose DMEM (LCD) for 24 h.

### Experimental procedures

*Segatella* preparation and treatment: *Segatella* was revived from Columbia blood agar plates and cultured in low-glucose medium. Bacterial suspensions were prepared at multiplicities of infection (MOI) 0.1 and 1 and applied to cardiomyocytes after doxorubicin exposure to establish the injury models.

Apoptosis assay: Cell death was quantified by flow cytometry after Annexin V-FITC/PI dual staining to determine the apoptotic index, allowing dose-response evaluation of *Segatella* and the protective effect of LCD.

Oxidative stress assessment: Intracellular ROS levels were measured with the DCFH- DA fluorescent probe; fluorescence intensity was quantified by flow cytometry and fluorescence microscopy to assess *Segatella*-induced oxidative damage and LCD-mediated mitigation.

Gene expression analysis: Quantitative real-time PCR (qPCR) was employed to quantify mRNA levels of apoptosis-related (Bax, Bcl-2), oxidative-stress-related (SOD1, CAT), and TLR4/MyD88/NF-κB pathway genes (TLR4, MyD88, NF-κB p65), thereby dissecting the transcriptional networks modulated by *Segatella* and LCD.

Protein expression analysis: Western blotting (WB) was used to determine the abundance and phosphorylation status of key proteins (cleaved caspase-3, p53, TLR4, MyD88, nuclear NF-κB p65), validating *Segatella*-induced pathway activation and the suppressive effect of LCD at the protein level.

Inflammatory cytokine measurement: Concentrations of TNF-α and IL-6 in cell-culture supernatants were quantified using commercially available enzyme-linked immunosorbent assay (ELISA) kits to comprehensively evaluate *Segatella*-triggered inflammation and the dynamic modulation of the inflammatory microenvironment by LCD. Detailed protocols are provided in Supplementary Methods.

**Fig. 22:**
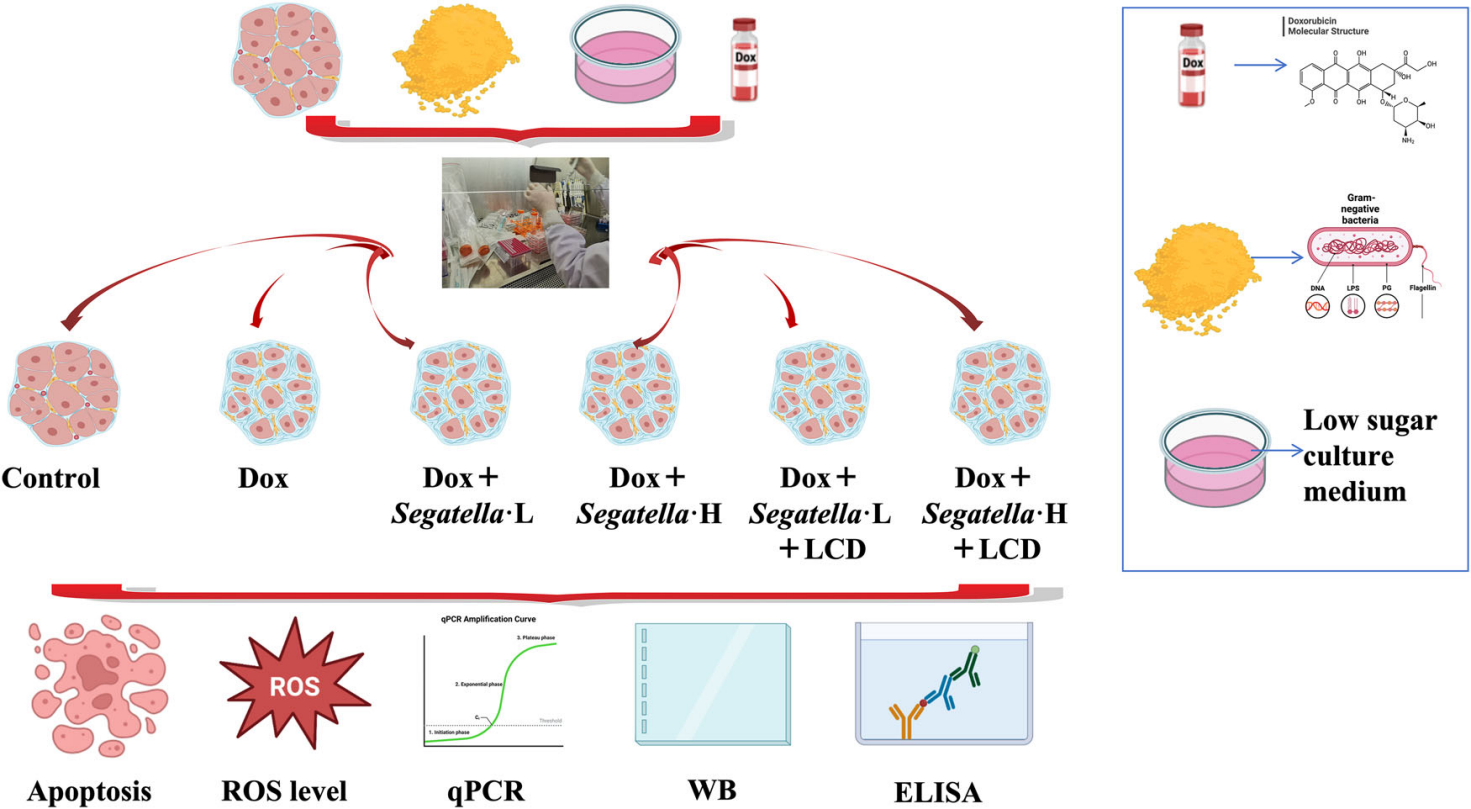
Schematic workflow of in vitro experiments. Control, untreated cardiomyocytes; Dox, doxorubicin-induced injury model; Dox + *Segatella*-L, injury plus low-dose *Segatella* (MOI = 0.1); Dox + *Segatella*-H, injury plus high-dose *Segatella* (MOI = 1); Dox + *Segatella*-L+LCD, injury plus low-dose *Segatella* and 20% low-carbohydrate medium; Dox + *Segatella*-H+LCD, injury plus high-dose *Segatella* and 20% low-carbohydrate medium.

The overall workflow of the human study is depicted in Fig. 22.

### In vivo study

**Animals and group allocation**

Specific-pathogen-free (SPF) grade Wistar rats (weighing ~300 g, half male and half female, aged 8–12 weeks) were purchased from SPF (Beijing) Biotechnology Co., Ltd (License No.: SCXK [Jing] 2024-0001). The experimental animals were randomly divided into six groups (*n* = 6 per group), with an experimental duration of 8 weeks.

**Sample size and study design**

The sample size of 6 rats per group was determined based on a formal power analysis conducted using G*Power software. We aimed to detect a medium effect size (Cohen’s *f* = 0.25) with a significance level (α) of 0.05 and a power (1-β) of 0.80. The analysis was conducted for a one-way ANOVA with 6 groups. The results indicated that a sample size of 6 rats per group (total of 36 rats) is sufficient to achieve the desired statistical power for our study. This sample size is consistent with previous studies in the field and has been shown to provide meaningful results in similar experimental designs.

The specific grouping is as follows:

- Control: healthy rats receiving intraperitoneal (i.p.) and intragastric (i.g.) saline and fed standard chow.
- *Segatella*: healthy rats receiving i.p. saline plus i.g. *Segatella* suspension (1 mL/100 g) on standard chow.
- *Segatella*+LCD: healthy rats receiving i.p. saline plus i.g. *Segatella* suspension and fed a low-carbohydrate diet.
- CHF: CHF induced by weekly i.p. doxorubicin (3 mg/kg for 8 weeks), with i.g. saline and standard chow.
- CHF + *Segatella*: CHF rats receiving i.g. *Segatella* suspension (1 mL/100 g) on standard chow.
- CHF + *Segatella*+LCD: CHF rats receiving i.g. *Segatella* suspension and fed a low-carbohydrate diet.

### Experimental diets and bacterial preparation

Standard chow contained 65% carbohydrate, 20% protein, and 8% fat (SY1001, Shuyu Biotech). The LCD was isocaloric but adjusted to 20% carbohydrate, 60% fat, and 20% protein and was custom-manufactured by the same supplier. *Segatella* (ATCC 27766) was reactivated on Columbia blood agar under anaerobic conditions, propagated in liquid medium with orbital shaking, and prepared as a 3.75 × 10^8^ CFU/mL suspension for gavage.

### CHF model induction and interventions

CHF was induced by weekly intraperitoneal doxorubicin (3.0 mg/kg for 8 weeks). From the first doxorubicin dose, animals were gavaged twice weekly with 1 mL/100 g body weight: controls received sterile saline, whereas intervention groups received the *Segatella* suspension or were switched to the LCD. A 20–22G gavage needle, lubricated prior to insertion, was advanced gently into the esophagus to deliver the inoculum.

### Safety assessment and physiological monitoring

Body weight was recorded weekly. Under isoflurane anesthesia, heart rate and blood pressure were obtained via a biological signal acquisition system. At study completion (week 8), echocardiography was performed to quantify Left Ventricular Ejection Fraction (LVEF), Left Ventricular Fractional Shortening(LVFS), Left Ventricular Internal Diameter in Diastole (LVIDd), and Left Ventricular Internal Diameter in Systole (LVIDs). Serum was collected at weeks 2, 4, and 8 for measurement of BNP and cTnI levels.

### Histological and molecular analyses

Upon study completion, hearts and fecal samples were harvested for histopathology, molecular profiling, and gut microbiota assessment as follows:

1. Cardiac histopathology

- Hematoxylin–eosin (HE) staining was used to evaluate cardiomyocyte morphology (cell swelling, disarray) and inflammatory infiltrates.
- Masson trichrome staining quantified interstitial fibrosis; blue collagen fractions (%) were determined using ImageJ to reflect myocardial remodeling.

2. Immunohistochemistry

Protein expression and localization of p53 (cellular stress marker), cleaved caspase-3 (apoptotic executioner), TLR4 (inflammatory receptor), and NF-κB p65 (transcription factor) were examined. Positive staining intensity was scored (H-score) with Image-Pro Plus software.

3. Protein validation (Western blot)

Cardiac levels of TLR4, MyD88 (TLR4 adaptor), and NF-κB p65 were assessed by Western blot, normalized to β-actin; band densities were quantified using ImageJ to gauge inflammatory pathway activation.

4. Gene and protein quantification (qPCR+ELISA)

- qPCR measured mRNA expression of inflammatory cytokines (TNF-α, IL-6) and pathway components (TLR4, MyD88, NF-κB p65).
- ELISA quantified TNF-α and IL-6 protein concentrations in cardiac homogenates, reflecting local inflammation.

5. Gut microbiota analysis

Fecal DNA was extracted, the V3–V4 region of 16S rRNA was amplified (primers 338F/806R), and sequencing was performed on the Illumina MiSeq platform. *Segatella* abundance, Shannon diversity, and the Firmicutes/Bacteroidetes (F/B) ratio were analyzed to link microbial structure with CHF status. Detailed protocols are provided in Supplementary Methods.

### Statistical analysis

All analyses were performed with SPSS version 22.0 (IBM Corp.). Continuous variables were first tested for normality. Normally distributed data are presented as mean ± SD and were compared among groups using one-way ANOVA. Non-normally distributed data are presented as median [interquartile range] and were analyzed with the Kruskal–Wallis test. For pairwise comparisons, Student’s *t*-test (normal distribution) or Wilcoxon rank-sum test (non-normal distribution) was applied. Categorical variables are expressed as *n* (%) and were compared using the χ² test or Fisher’s exact test, as appropriate.

**Fig. 23:**
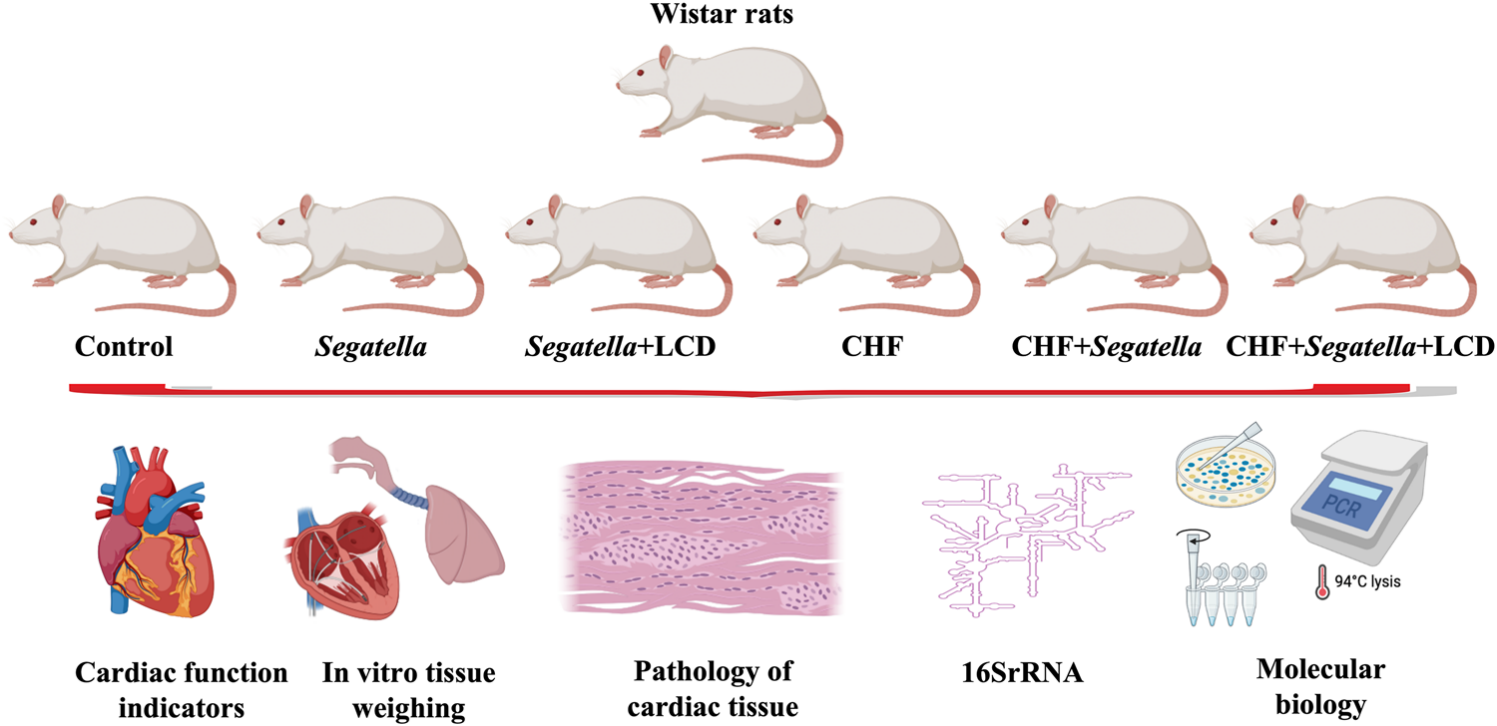
Experimental workflow of the in vivo study. Control, healthy rats receiving saline gavage and standard chow; *Segatella*, healthy rats gavaged with *Segatella* suspension and fed standard chow; *Segatella*+LCD, healthy rats gavaged with *Segatella* suspension and maintained on a low-carbohydrate diet; CHF, chronic heart failure induced by doxorubicin; CHF+*Segatella*, CHF rats gavaged with *Segatella* suspension and fed standard chow; CHF+*Segatella*+LCD, CHF rats gavaged with *Segatella* suspension and maintained on a low-carbohydrate diet.

The overall workflow of the human study is depicted in Fig. 23.

All methods were performed in accordance with the relevant guidelines and regulations as stipulated by the Declaration of Helsinki and the ARRIVE guidelines for animal research.

## Supplementary information

Supplementary File - Results

Supplementary File - Methods

Full and Uncropped Western Blots

## Data Availability

The datasets utilized in the current study are available from the corresponding author upon reasonable request.
