# Supplementary material for: Segatella exacerbates chronic heart failure via TLR4/NF-κB pathway and therapeutic potential of low-carbohydrate diet
Source: Cell Death Discov. 2025 Oct 21;11:472. doi: 10.1038/s41420-025-02762-9 (PMC12541022; doi:10.1038/s41420-025-02762-9)
Supplement: Supplementary file 1 — Supplementary File - Results [file 41420_2025_2762_MOESM1_ESM.docx]

***Supplementary Results 1:***

****Baseline Characteristics of the Study Population****

**Baseline characteristics of 152 CHF patients and 105 healthy controls were analyzed (Tables 1). The mean age in the CHF group was 57.97 years, with 64% males; the control group had a mean age of 56.67 years, with 31% males. There was no significant difference in age between the two groups (*P*=0.778), but gender distribution differed significantly (*P*=0.002). Laboratory indices revealed that the CHF group had significantly lower LVEF (39.28% vs. 62.52%, *P*<0.001) and significantly higher NT-proBNP levels (3080.00 ng/L vs. 45.25 ng/L, *P*<0.001) compared to the control group. Additionally, the CHF group exhibited significantly higher levels of inflammatory markers (e.g., C-reactive protein) and metabolic parameters (e.g., total cholesterol, fasting blood glucose). Comorbidity analysis indicated that the incidence of ICM, hypertensive heart disease, VHD, DCM, T2DM, and pulmonary hypertension was significantly higher in the CHF group. Medication history showed that the usage rates of ACEI/ARB, diuretics, β-blockers, SGLT-2 inhibitors, aspirin, and statins were significantly higher in the CHF group. These results provide important baseline data support for subsequent research.**

| Characteristic | Control Group (n = 105) | CHF Group (n = 152) | χ²/z/t Value | P Value |
| --- | --- | --- | --- | --- |
| Age (years) | 56.67±10.84 | 57.97±9.76 | −0.197 | 0.778 |
| Male (%) | 33(31) | 98(64) | 9.506 | 0.002 |
| BMI (kg/m²) | 24.39±3.57 | 27.45±5.58 | -3.255 | 0.002 |
| Systolic BP (mmHg) | 116.12±11.76 | 117.68±20.36 | -0.471 | 0.639 |
| Diastolic BP (mmHg) | 72.22±9.44 | 74.00±13.81 | -0.807 | 0.422 |
| Heart Rate (bpm) | 80.31±12.04 | 80.28±15.13 | 0.077 | 0.939 |
| Smoking History (%) | 48(46) | 65(43) | 0.001 | 0.975 |
| Alcohol History (%) | 4(4) | 12(8) | 0.199 | 0.656 |
| NYHA(%) | | | | |
| I | - | 0 | - | - |
| II | - | 9(6) | - | - |
| III | - | 78(51) | - | - |
| IV | - | 65(43) | - | - |
| Laboratory Parameters | | | | |
| LVEF (%) | 62.52±1.88 | 39.28±8.85 | 18.169 | ＜0.001 |
| NT-proBNP (ng/L) | 45.25(22.13，86.40) | 3080.00(892.0，6070.0) | -8.085 | ＜0.001 |
| WBC (*10⁹/L) | 6.79±1.93 | 6.26±1.70 | 1.450 | 0.150 |
| Neutrophils (%) | 63.34 | 58.09 | 3.909 | ＜0.001 |
| Lymphocytes (%) | 25.04 | 31.80 | 5.370 | ＜0.001 |
| Hemoglobin (g/L) | 136.24±18.30 | 134.90±20.40 | 0.346 | 0.730 |
| Platelets (*10⁹/L) | 253.16±74.30 | 201.80±57.84 | 3.871 | ＜0.001 |
| CRP (mg/L) | 5.85(3.50，8.20) | 14.12(13.05，15.19) | -5.67 | ＜0.001 |
| K⁺ (mmol/L) | 3.63±0.34 | 3.75±0.46 | -1.420 | 0.159 |
| Na⁺ (mmol/L) | 140.18±2.43 | 140.09±3.86 | 0.150 | 0.881 |
| Apolipoprotein AI (g/L) | 1.25±0.26 | 1.03±0.34 | 3.493 | ＜0.001 |
| AST (U/L) | 28±4 | 38±5 | 3.2 | 0.007 |
| ALT (U/L) | 30±5 | 45±7 | 2.8 | 0.02 |
| Total Protein (g/L) | 74.75±6.37 | 68.00±9.96 | 4.013 | ＜0.001 |
| Creatinine (µmol/L) | 63.36(51.00，69.04) | 76.97(65.09，107.46) | -5.155 | ＜0.001 |
| Uric Acid (µmol/L) | 272.66(241.15，321.47) | 338.46(288.71，436.65) | -3.627 | ＜0.001 |
| Triglycerides (mmol/L) | 1.03(0.71，1.54) | 1.05(0.72，1.60) | -0.195 | 0.845 |
| Total Cholesterol (mmol/L) | 4.34±0.87 | 3.86±1.18 | 2.326 | 0.022 |
| HDL (mmol/L) | 1.15±0.29 | 0.96±0.34 | 3.022 | 0.003 |
| LDL (mmol/L) | 2.81±0.68 | 2.50±0.90 | 1.930 | 0.057 |
| Fasting Blood Glucose (mmol/L) | 5.09±0.72 | 5.78±1.54 | -2.796 | 0.007 |
| Comorbidities | | | | |
| Ischemic Cardiomyopathy | 0 | 74(49) | 112.5 | ＜0.001 |
| Hypertensive Heart Disease | 0 | 44(29) | 62.65 | ＜0.001 |
| Valvular Heart Disease | 0 | 20(13) | 40 | ＜0.001 |
| Dilated Cardiomyopathy | 0 | 14(9) | 18.4 | ＜0.001 |
| T2DM | 6(6) | 55(36) | 13.906 | ＜0.001 |
| Hyperlipidemia | 21(21) | 29(19) | 0.988 | 0.320 |
| Pulmonary Hypertension | 0 | 67(44) | 25.372 | ＜0.001 |
| Medication History | | | | |
| ACEI/ARB (%) | 0(0) | 133(88) | 1123.5 | ＜0.001 |
| Diuretics (%) | 0(0) | 135(89) | 1278.2 | ＜0.001 |
| β-Blockers (%) | 13(13) | 139(91) | 2119.7 | ＜0.001 |
| SGLT-2 Inhibitors (%) | 2(2) | 59(39) | 21.2 | ＜0.001 |
| Aspirin (%) | 2(2) | 91(60) | 104.7 | ＜0.001 |
| Statins (%) | 10(9) | 86(57) | 85.6 | ＜0.001 |

**Table 1 Characteristics of Participants of the Two Groups**

Note: Continuous variables with normal distribution are presented as mean ± SD, while non-normally distributed variables are shown as median [interquartile range]. Categorical variables are expressed as n (%). P values were calculated using independent - samples t - test for normally distributed variables, Mann–Whitney U test for non - normally distributed variables, and χ² test for categorical variables. Abbreviations: BMI, body mass index; ACEI, angiotensin - converting - enzyme inhibitor; ARB, angiotensin - II receptor blocker; SGLT - 2i, sodium - glucose cotransporter - 2 inhibitor. Controls, healthy control group; CHF, chronic heart failure group.

***Supplementary Results 2:***

**Table 2 Diagnostic Performance Indicators of the Differential Bacteria Model**

| Indicator | AUC Value | 95% CI | P Value | Sensitivity | Specificity | PPV | NPV |
| --- | --- | --- | --- | --- | --- | --- | --- |
| Training Set (n=80) | 0.836 | 0.716-0.951 | <0.001 | 81.30% | 77.50% | 82.30% | 75.60% |
| Validation Set (n=20) | 0.772 | 0.436-1 | 0.003 | 70.00% | 75.00% | 77.80% | 66.70% |
| Total Sample (n=100) | 0.821 | 0.699-0.919 | <0.001 | 80.00% | 75.00% | 80.00% | 75.00% |

Note: The table lists the AUC values, 95% confidence intervals, *P* values, sensitivity, specificity, positive predictive value (PPV), and negative predictive value (NPV) for the training set, validation set, and total sample. These indicators collectively reflect the predictive performance of the model in CHF diagnosis.。

**Table 3 Diagnostic Performance Indicators**

| Indicator | AUC Value | 95% CI | *P* Value | Sensitivity | Specificity | PPV | NPV |
| --- | --- | --- | --- | --- | --- | --- | --- |
| Combined Model | 0.861 | 0.755-0.949 | <0.001 | 83.9% | 77.8% | 83.90% | 74.10% |
| Radiomics Model | 0.995 | 0.974-1.000 | <0.001 | 85.2% | 82.1% | 85.60% | 77.40% |

Note: The table lists the key diagnostic performance indicators for the combined model and the extended model, including AUC values, 95% confidence intervals, *P* values, sensitivity, specificity, PPV, and NPV, demonstrating the improvements of the extended model across various metrics.

***Supplementary Results 3:***

****Identification of Differential Metabolites****

**In this study, to identify biologically significant differential metabolite biomarkers, we employed the PLS-DA model and screened based on the VIP (Variable Importance in Projection) values derived from the model. The criteria for selection included: 1) Fold Change (FC) ≥1.2 or ≤0.8333; 2) *P* < 0.05; and 3) VIP > 1, indicating a higher contribution of the metabolite in distinguishing between the two groups of samples. Based on these criteria, a total of 47 differential metabolites were identified across both positive and negative ion modes, with 24 in the positive ion mode (as shown in Table 4) and 23 in the negative ion mode (as shown in Table 5).**

**Table 4 Significant Differential Metabolites in Positive Ion Mode**

| Number | m/z | Metabolite | VIP | *P* | FC |
| --- | --- | --- | --- | --- | --- |
| P1 | 181.07 | Carbohydrates and carbohydrate conjugates | 4.92 | 0.00 | 0.61 |
| P2 | 111.01 | Short-chain keto acids and derivatives | 21.2 | 0.01 | 0.63 |
| P3 | 1035.66 | Gangliosides [SP0601] | 3.58 | 0.01 | 1.42 |
| P4 | 590.35 | Glycerophosphocholines | 39.6 | 0.01 | 1.29 |
| P5 | 590.35 | Monoacylglycerophosphocholines [GP0105] | 39.6 | 0.01 | 1.29 |
| P6 | 554.34 | Glycerophosphoethanolamines | 14.5 | 0.03 | 1.59 |
| P7 | 554.34 | Monoacylglycerophosphoethanolamines [GP0205] | 14.5 | 0.03 | 1.59 |
| P8 | 554.35 | 1-alkyl,2-acylglycerophosphocholines [GP0102] | 26.2 | 0.01 | 1.68 |
| P9 | 447.10 | Stilbene glycosides | 1.73 | 0.00 | 0.78 |
| P10 | 165.05 | Anisoles | 3.92 | 0.00 | 0.50 |
| P11 | 204.07 | Indolyl carboxylic acids and derivatives | 16.9 | 0.04 | 0.73 |
| P12 | 321.08 | Sugar acids and derivatives | 2.22 | 0.04 | 0.42 |
| P13 | 399.12 | Pyrimidines and pyrimidine derivatives | 6.26 | 0.04 | 0.54 |
| P14 | 605.24 | Chlorins | 6.37 | 0.00 | 0.44 |
| P15 | 541.26 | Cholesterol and derivatives [ST0101] | 1.58 | 0.01 | 0.72 |
| P16 | 527.21 | Glucuronides [ST0501] | 4.22 | 0.03 | 0.67 |
| P17 | 527.21 | Steroidal glycosides | 4.22 | 0.03 | 0.67 |
| P18 | 665.32 | Steroid lactones | 9.87 | 0.03 | 0.65 |
| P19 | 435.27 | C24 bile acids, alcohols, and derivatives [ST0401] | 4.26 | 0.04 | 1.62 |
| P20 | 393.26 | Monoacylglycerols [GL0101] | 4.07 | 0.04 | 0.52 |
| P21 | 279.23 | Unsaturated fatty acids [FA0103] | 7.81 | 0.02 | 0.65 |
| P22 | 433.28 | Vitamin D3 and derivatives [ST0302] | 2.01 | 0.00 | 0.75 |
| P23 | 533.33 | Triterpenoids | 6.95 | 0.02 | 0.75 |
| P24 | 824.41 | Ceramide phosphoinositols [SP0303] | 6.3 | 0.04 | 0.75 |

Note:*P*-value: The *P*-value obtained through statistical testing, used to assess the statistical significance of differences.FC (Fold Change): The change in expression levels of metabolites between patients with chronic heart failure and healthy controls. An FC ≥ 1.2 (upregulation) or ≤ 0.8333 (downregulation) indicates a significant difference.VIP (Variable Importance in Projection): A metric in the PLS-DA model used to measure the importance of variables. A VIP value > 1 indicates that the metabolite has a higher contribution in distinguishing between the two groups of samples.m/z (mass-to-charge ratio): A parameter in mass spectrometry used to characterize the mass-to-charge ratio of ions.Metabolite: A small molecule involved in metabolism.

**Table 5 Significant Differential Metabolites in Negative Ion Mode**

| Number | m/z | Metabolite | VIP | *P* | FC |
| --- | --- | --- | --- | --- | --- |
| N1 | 182.05 | Amino acids, peptides, and analogues | 7.3 | 0.00 | 1.41 |
| N2 | 150.11 | Medium-chain hydroxy acids and derivatives | 4.23 | 0.04 | 0.77 |
| N3 | 482.33 | Monoacylglycerophosphoethanolamines [GP0205] | 51 | 0.05 | 1.39 |
| N4 | 482.33 | Glycerophosphoethanolamines | 51 | 0.05 | 0.39 |
| N5 | 227.10 | Amines | 5.56 | 0.04 | 0.57 |
| N6 | 595.42 | C40 isoprenoids (tetraterpenes) [PR0107] | 1.31 | 0.03 | 1.41 |
| N7 | 146.06 | Indolines | 26.3 | 0.00 | 1.61 |
| N8 | 94.06 | Aniline and substituted anilines | 13 | 0.00 | 1.60 |
| N9 | 103.05 | Benzoyl derivatives | 22.4 | 0.00 | 1.75 |
| N10 | 93.07 | Toluenes | 70.1 | 0.00 | 1.77 |
| N11 | 181.07 | Purines and purine derivatives | 3.99 | 0.01 | 0.20 |
| N12 | 191.08 | Pyridoxamines | 10.1 | 0.01 | 0.67 |
| N13 | 683.34 | Carbohydrates and carbohydrate conjugates | 6.73 | 0.01 | 0.26 |
| N14 | 259.05 | Tryptamines and derivatives | 17.1 | 0.01 | 1.77 |
| N15 | 245.13 | Pyrimidine 2'-deoxyribonucleosides | 22.2 | 0.01 | 1.55 |
| N16 | 438.24 | Eicosanoids | 20 | 0.00 | 1.56 |
| N17 | 438.24 | Leukotrienes [FA0302] | 20 | 0.00 | 0.56 |
| N18 | 583.25 | Bilirubins | 56 | 0.02 | 0.37 |
| N19 | 377.15 | Alloxazines and isoalloxazines | 11.7 | 0.04 | 1.31 |
| N20 | 356.28 | Hydroxy/hydroperoxyeicosatrienoic acids [FA0305] | 5.33 | 0.05 | 0.32 |
| N21 | 630.27 | Coumarin glycosides | 4.18 | 0.00 | 0.12 |
| N22 | 400.34 | Fatty acid esters | 5.21 | 0.04 | 0.56 |
| N23 | 400.34 | Fatty acyl carnitines [FA0707] | 5.21 | 0.04 | 0.56 |

***Supplementary Results 4:***

**Table 6 Performance Indicators of Differential Metabolites in Positive and Negative Ion Modes for CHF Diagnosis**

| Indicator | AUC Value | 95% CI | *P* Value | Sensitivity | Specificity | PPV | NPV |
| --- | --- | --- | --- | --- | --- | --- | --- |
| Positive lon Model | 0.823 | 0.698-0.925 | <0.001 | 75% | 82% | 78% | 80% |
| Negative lon Model | 0.878 | 0.793-0.958 | <0.001 | 80% | 85% | 82% | 83% |

Note: The AUC refers to the area under the receiver operating characteristic curve, the 95% CI represents the 95% confidence interval, and the P value measures the significance of diagnostic performance differences. Sensitivity, specificity, PPV (positive predictive value), and NPV (negative predictive value) are used to assess diagnostic capability. The diagnostic performance of metabolites in the negative ion mode is relatively superior.
