# Supplementary material for: Segatella exacerbates chronic heart failure via TLR4/NF-κB pathway and therapeutic potential of low-carbohydrate diet
Source: Cell Death Discov. 2025 Oct 21;11:472. doi: 10.1038/s41420-025-02762-9 (PMC12541022; doi:10.1038/s41420-025-02762-9)
Supplement: Supplementary file 2 — Supplementary File - Methods [file 41420_2025_2762_MOESM2_ESM.docx]

***Part 1***

**1 Research Content and Methods**

**1.1 Study Design and Collection of Demographic Data**

**1.1.1 Study Design**

This is a cross-sectional study, which consecutively enrolled patients with chronic heart failure (CHF) who were hospitalized and diagnosed in the Department of Heart Failure, First Affiliated Hospital of Xinjiang Medical University from February 2024 to August 2024. The study design strictly adheres to the ethical principles of the *Declaration of Helsinki* and has been approved by the Ethics Committee of the First Affiliated Hospital of Xinjiang Medical University (approval number: K202402-05). The study protocol has been registered on the Chinese Clinical Trial Registry (trial registration number: ChiCTR1900027476). All eligible patients signed informed consent forms before participating in the study. The diagnosis of CHF was based on the *2023 Chinese Guidelines for the Diagnosis and Treatment of Heart Failure* **^[1]^** , with reference to the *2021 European Society of Cardiology Guidelines for the Diagnosis and Treatment of Heart Failure* **^[2]^** and the *2022 American College of Cardiology/American Heart Association (ACC/ AHA) Guidelines for the Management of Heart Failure* **^[3]^** , to ensure the international consistency of diagnostic criteria and the comprehensiveness of evidence-based foundations.

**1.1.2 Inclusion Criteria**

**1.1.2.1 Experimental Group**

(1) Aged between 18 and 80 years;

(2) Definitively diagnosed with CHF according to the *2023 Chinese Guidelines for the Diagnosis and Treatment of Heart Failure* **^[1]^**, *2021 European Society of Cardiology Guidelines for the Diagnosis and Treatment of Heart Failure* **^[2]^** , and *2022 ACC/AHA Guidelines for the Management of Heart Failure* **^[3]^** with a clear disease history. A clear disease history is defined as the patient having continuous medical records, including at least two heart failure-related diagnostic records (such as outpatient medical records, inpatient medical records, or diagnostic reports from other formal medical institutions) with an interval of more than 6 months, and the records contain detailed descriptions of heart failure symptoms, diagnostic basis, and treatment process;

(3) Samples cover CHF patients of different ages, genders, and etiologies to ensure the generalizability of the study results;

(4) All patients underwent examinations such as echocardiography and serum NT-proBNP level measurement. The above examinations were all completed at the First Affiliated Hospital of Xinjiang Medical University;

(5) New York Heart Association (NYHA) cardiac function class is II or higher. NYHA classification was evaluated by at least two attending physicians with rich clinical experience in cardiovascular diseases based on the patient’s daily activity ability, degree of dyspnea, and other clinical manifestations to ensure the accuracy and consistency of the classification.

**1.1.2.2 Control Group**

(1) Aged between 18 and 80 years;

(2) No history of cardiovascular diseases. Cardiovascular diseases were excluded through comprehensive judgment using multiple examination methods, including detailed medical history inquiry, comprehensive physical examination, echocardiography, electrocardiography, and ambulatory electrocardiographic monitoring. Meanwhile, the absence of CHF was further confirmed by at least two attending physicians based on clinical symptoms, signs, laboratory tests (including blood routine, blood biochemical analysis, inflammatory indicators, coagulation function, thyroid function tests, etc.), and electrocardiography.

**1.1.3 Exclusion Criteria**

(1) Complicated with other malignant tumors;

(2) Severe lesions in important organs such as the liver, lungs, and kidneys;

(3) Suffering from severe infectious diseases such as active systemic lupus erythematosus and severe pulmonary fungal infection; suffering from immune diseases such as active rheumatoid arthritis and systemic sclerosis; having language communication barriers, mental abnormalities, etc.;

(4) Suffering from digestive system diseases such as Crohn’s disease, ulcerative colitis, and irritable bowel syndrome;

(5) History of antibiotic use within the past month;

(6) Having undergone major cardiac surgery (such as coronary artery bypass grafting, heart valve replacement, etc.) within the past 3 months;

(7) History of long-term use of drugs that may affect intestinal flora, such as immunosuppressants or glucocorticoids (continuous use for more than 3 months).

**1.1.4 Diagnostic Criteria for Diseases**

The clinical diagnosis of dilated cardiomyopathy (DCM) follows the criteria in the *Chinese Guidelines for the Diagnosis and Treatment of Dilated Cardiomyopathy (2018)* **^[4]^** , which requires objective evidence of ventricular enlargement and reduced myocardial systolic function: 1) Left ventricular end-diastolic diameter (LVEDd) > 50 mm (for females) and > 55 mm (for males) (or > 117% of the predicted value based on age and body surface area, i.e., 2 times the standard deviation + 5% of the predicted value); 2) Left ventricular ejection fraction (LVEF) < 45% and left ventricle fractional shortening (LVFS) < 25% **^[3]^** ; 3) Excluding hypertension, valvular heart disease (VHD), congenital heart disease, or ischemic cardiomyopathy (ICM) at the time of onset, which is identified through differential diagnosis using detailed medical history inquiry, physical examination, echocardiography, coronary angiography, and other examination methods **^[5]^** .

The clinical diagnosis of ICM is based on the 2019 European Society of Cardiology Guidelines for the Diagnosis and Management of Chronic Coronary Syndromes **^[6]^** : 1) Evidence of coronary artery disease: history of angina pectoris or myocardial infarction (≥ 6 months), with coronary angiography showing stenosis ≥ 50%; 2) Cardiac enlargement: echocardiography showing ventricular enlargement (abnormal indicators such as LVEDd); 3) Heart failure symptoms: recurrent heart failure symptoms, confirmed by electrocardiography, echocardiography, NT-proBNP, etc.

The clinical diagnosis of VHD is based on criteria such as the *Chinese Expert Consensus on Clinical Pathway for Transcatheter Aortic Valve Replacement (2021 Edition)* and *Expert Consensus on Standardized Echocardiographic Examination for Adult Valvular Heart Disease* **^[7-8]^** : 1) Abnormal valve structure: echocardiography showing leaflet thickening, calcification, adhesion, or prolapse, accompanied by reduced valve orifice area (e.g., aortic valve stenosis < 15 mm²) or increased regurgitant jet area (e.g., mitral regurgitant jet area/left atrial area ratio ≥ 20%); 2) Heart failure symptoms such as dyspnea, fatigue, and edema, combined with heart murmur on auscultation, and excluding other heart diseases.

For the inclusion criteria of patients with type 2 diabetes mellitus (T2DM) and hypertension: the diagnostic criteria for T2DM include 1) Blood glucose level: fasting blood glucose ≥ 7.0 mmol/L and/or 2-hour postprandial blood glucose ≥ 11.1 mmol/L **^[9]^** ; 2) Treatment records: having a diagnosis of T2DM and currently using hypoglycemic drugs. The diagnostic criteria for hypertension are 1) Blood pressure level: systolic blood pressure ≥ 140 mmHg and/or diastolic blood pressure ≥ 90 mmHg without the use of antihypertensive drugs (measured at least three times on the same day) **^[10]^**; 2) Treatment records: having a history of hypertension and currently taking antihypertensive drugs.

**1.1.5 Collection of Clinical Data and Samples**

Recording of baseline characteristics: including gender, age, height, weight (based on which body mass index [BMI] = weight (kg)/height² (m²) was calculated), blood pressure at admission, medical history data (time of first occurrence of heart failure symptoms, time of diagnosis and treatment intervention, discharge diagnosis results), comorbidities (including etiologies of CHF such as coronary artery disease [CAD], DCM, VHD, hypertension, etc.), lifestyle information (smoking and drinking habits), medication status, etc.

Collection and detection of blood samples: Peripheral venous blood samples were collected after the patients fasted for 12 hours to determine blood routine, blood biochemistry, inflammatory markers, coagulation function, thyroid function, and NT-proBNP levels. Meanwhile, the results of examinations such as electrocardiography, echocardiography, coronary angiography, and carotid vascular ultrasound were recorded.

Collection and quality control of fecal samples: 1) Samplers: Each participant was provided with a dedicated sampler that had undergone strict high-pressure steam sterilization to avoid exogenous microbial contamination; 2) Training: Participants were trained in detail on how to correctly collect fecal samples through on-site demonstrations and written instruction materials (such as avoiding urine contamination, collecting feces from different parts to ensure sample representativeness, and promptly sealing the samples after collection, etc.); 3) Transportation and preservation: After collection, samples were immediately placed in sterile cryopreservation tubes, transported under refrigeration with dry ice, and the temperature was monitored in real-time. Upon arrival at the laboratory, the samples were divided into five portions (200 mg each), quickly frozen in liquid nitrogen, and then transferred to a -80°C low-temperature refrigerator for preservation.

**1.1.6 Sample Size Calculation**

Based on the consensus in heart failure microbiome research **^[11-13]^**, the sample size was estimated using PASS 21.0.3 software. The significance level (α) was set at 0.05 (two-tailed test), and the statistical power (1-β) was 0.8. The effect size (δ=0.5) was referenced to the standards for gut microbiota-cardiovascular disease association studies **^[11]^**, and the overall standard deviation (σ=1.0) was derived from recent CHF cohort study data **^[12]^**. The calculation was performed using the formula for comparing means of two independent samples：


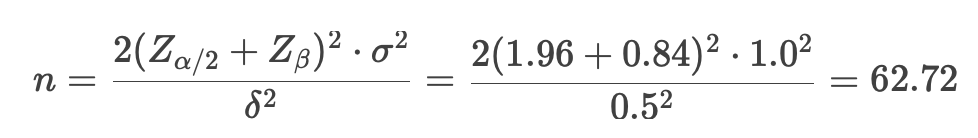


In accordance with clinical research design specifications**^[13]^**, considering a 20% loss-to-follow-up rate and the requirement for data integrity control, the final sample size was determined to be at least 76 cases per group (62.72×1.2). Through screening via the hospital's electronic medical record system, a total of 257 participants were enrolled (152 cases in the CHF group and 105 cases in the healthy control group), which meets the minimum sample size requirements for epidemiological studies **^[14-15]^** .

**1.1.7 Subgroup Sampling Process**

To further explore the association between the gut microbiome and CHF, this study performed stratified sampling based on key factors such as age, gender, and NYHA functional class from the initial cohort of 257 subjects:

(1) Stratification design: Subjects were divided into three age strata: <50 years, 50-65 years, and >65 years. Within each age stratum, they were categorized by gender (male and female). For CHF patients, further subdivision was done according to NYHA functional class (Class II, III, and IV).

(2) Random sampling: Using a computer-generated randomization list, 50 CHF patients and 50 healthy controls were randomly selected proportionally from each (sub-stratum) to form a subgroup for metagenomic and taxonomic analyses.

(3) Validation of matching effectiveness: Statistical tests were conducted on variables such as age and gender after matching, and the results showed no significant differences between the two groups (age: *P*=0.778; gender: *P*=0.95), indicating successful matching.

**1.1.8 Statistical Analysis of Clinical Data**

Statistical methods in this study were selected based on data types. Normally distributed continuous variables were described as mean±SD, and differences between groups were compared using ANOVA. Non-normally distributed variables were expressed as median (IQR) and analyzed using the Kruskal-Wallis test. For comparisons between two groups, the t-test or Wilcoxon rank-sum test was used. Categorical data were presented as n (%), and analyzed using the chi-square test or Fisher’s exact test. If significant differences were found in multi-group comparisons, pairwise comparisons were adjusted using the Bonferroni method. Subgroup analyses focused on differences in gut microbiota and metabolites between groups, as well as their correlations with clinical indicators, to reveal the intrinsic association between the gut microbiota and CHF.

**1.2 Metagenomic Sequencing**

We used metagenomic sequencing technology to analyze the characteristics of the gut microbiota and its association with CHF. Metagenomics enables direct extraction of DNA from all microorganisms in environmental samples, thereby facilitating in-depth exploration of the genetic composition and functions of microbial communities **^[16]^**. We collected fecal samples from the subjects, which contained the genetic material of all microorganisms in the human gut environment. After extracting DNA from the samples and performing sequencing, we were able to analyze and interpret the diversity, structure, functions of the microbial community, and its association with the environment.

**1.2.1 DNA Extraction**

DNA was extracted from samples using the MagPure Stool DNA KF Kit B (MAGEN, Guangzhou) according to the manufacturer’s instructions. Briefly, 100–200 mg of sample was added to a centrifuge tube containing grinding beads, followed by the addition of 1 mL Buffer ATL/PVP-10. The mixture was ground using a high-speed grinder (Jingxin, Shanghai) and then incubated at 65°C for 20 min for lysis, followed by centrifugation at 14,000 × g for 5 min. The supernatant was transferred to a new tube, and 0.6 mL Buffer PCI was added. After vortexing for 15 s, the mixture was centrifuged at 18,213 × g for 10 min. The supernatant was transferred to a deep-well plate containing magnetic bead binding solution (including multiple reagents), and the corresponding program was run on a Kingfisher instrument (Thermo Fisher, USA). After completion, the DNA solution in the deep-well plate containing Elution Buffer was transferred to a 1.5 mL centrifuge tube for storage.

**1.2.2 Library Preparation and Sequencing**

For library preparation, 2×Phanta Max Master Mix (VAZYME, China) polymerase was used, with the degenerate forward (F) and reverse (R) PCR primers listed in Table 1-1 to amplify the V3V4/V4/ITS1/ITS2 variable regions of bacterial/fungal 16S/ITS rDNA. The PCR reaction system was 50 μL, containing 30 ng of template and fusion PCR primers. The PCR program was as follows: initial denaturation at 95°C for 3 min, followed by 30 cycles of denaturation at 95°C for 15 s, annealing at 56°C for 15 s, and extension at 72°C for 45 s, with a final extension at 72°C for 5 min. PCR products were purified using DNA selection magnetic beads (BGI, LB00V60). Qualified libraries were sequenced on the Illumina HiSeq platform (BGI, Shenzhen, China) with PE300/250 read lengths.

**Table 1-1 Primers used in this work**

| Region | Primer Name | Primer Sequence |
| --- | --- | --- |
| V3V4Region Primers | 338F | ACTCCTACGGGAGGCAGCAG |
|  | 806R | GGACTACHVGGGTWTCTAAT |
| V4Region Primers | 515F | GTGCCAGCMGCCGCGGTAA |
|  | 806R | GGACTACHVGGGTWTCTAAT |
| ITS1Region Primers | its1 | CTTGGTCATTTAGAGGAAGTAA |
|  | its2 | GCTGCGTTCTTCATCGATGC |
| ITS2Region Primers | its3 | GCATCGATGAAGAACGCAGC |
|  | its4 | TCCTCCGCTTATTGATATGC |

**1.2.3 Data Quality Control**

The raw sequencing data were filtered to retain high-quality data (Clean data) for subsequent analyses. Reads were assembled into Tags based on their overlapping regions, and these Tags were then clustered into Operational Taxonomic Units (OTUs). OTUs, a taxonomic unit used for analyzing microbial community structure, are defined as clusters of sequences with a similarity threshold (typically 97% sequence similarity), each representing a potential microbial species or taxon. Clustering sequences into OTUs simplifies data analysis while enabling classification and comparison of microbial community compositions. Through alignment with reference databases and species annotation, further analyses—including sample species diversity analysis, inter-group species difference analysis, correlation analysis, and model prediction—were conducted based on OTU clustering results and annotation information.

**1.2.3.1 Data Filtering**

To obtain high-quality Clean Data, the following processing steps were applied to raw sequencing data **^[17]^** :

(1) Quality control: iTools Fqtools fqcheck (v.0.25) was used for preliminary quality assessment of raw sequencing data, with a focus on base quality distribution. The proportion of bases with a quality score ≥ Q20 (error rate ≤ 1%) was required to exceed 80%; samples failing this criterion underwent further processing or re-sequencing. Additionally, adapter sequence contamination was detected, and sequencing depth was evaluated. Based on the complexity of the target microbial community in this study, the effective sequencing depth for each sample was determined to cover ≥ 95% of species in the target community, ensuring subsequent analyses accurately reflect sample microbial information.

(2) Cutadapt v2.6 software was used to remove primer and adapter contamination from reads matching the primers, enabling precise extraction of target region fragments. This software efficiently identifies and removes relevant sequences via specific algorithms to avoid interference with subsequent analyses; parameters were strictly set according to software instructions to ensure reliable removal efficiency.

(3) A 30-bp sliding window was applied: if the average quality score within a window was < 20, the terminal sequence of the read was truncated from the window start, as low-quality bases can lead to erroneous species identification and analytical bias. After processing, reads shorter than 75% of the original read length were removed. Automated processing via script writing improved efficiency and ensured accuracy of data handling.

(4) Readfq (v1.0) software was used to exclude reads containing unknown "N" bases, as ambiguous bases ("N") compromise analytical accuracy. Appropriate parameters were set during software operation to accurately identify and remove such reads, with the number and proportion of excluded reads recorded for data quality assessment.

(5) Readfq (v1.0) software was used to remove low-complexity reads containing 10 consecutive identical bases (A/T/C/G). These reads, arising from technical errors, are analytically uninformative and increase data noise; their removal enhances data quality and analytical reliability, ultimately yielding high-quality Clean Data to support subsequent analyses.

**1.2.3.2 Tags Assembly**

FLASH software (Fast Length Adjustment of Short reads, v1.2.11) **^[18]^** was used for sequence assembly, merging paired-end reads into a single sequence via overlapping regions to generate hypervariable region Tags. Assembly parameters were set as follows: minimum match length = 15 bp, and mismatch rate in overlapping regions ≤ 0.1.

**1.2.3.3 Statistical Analysis of OTU Clustering Results**

The DADA2 (Divisive Amplicon Denoising Algorithm) method in QIIME2 was used for data denoising to obtain Amplicon Sequence Variants (ASVs) with 100% sequence similarity and generate a feature table. Key steps included:

(1) Importing filtered paired-end sequences using `qiime tools import`;

(2) Constructing the feature table based on the DADA2 method via the `qiime dada2 denoise-paired` command;

(3) Converting the feature table to a viewable format using `qiime tools export`.

**1.2.3.4 Species Annotation and Functional Prediction**

1.2.3.4 Species Annotation and Functional Prediction

Sequencing Depth:

To ensure the comprehensiveness of the microbial community analysis, the sequencing depth was meticulously controlled. Each sample was sequenced to a depth that ensured coverage of at least 95% of the species within the target microbial community. This stringent requirement was set to provide a detailed and accurate representation of the microbial composition and diversity in each sample.

Quality Control Standards:

Quality control was a critical step in ensuring the reliability of the sequencing data. The following standards were strictly adhered to: (1) The proportion of bases with a quality score ≥ Q20 (error rate ≤ 1%) was required to exceed 80%, ensuring high accuracy of the sequencing data; (2) Primer and adapter contamination were removed using Cutadapt software to eliminate potential biases; (3) A 30-bp sliding window approach was employed, and if the average quality score within the window was < 20, the terminal sequence of the read was truncated to remove low-quality regions; (4) Reads containing unknown 'N' bases were excluded using Readfq software to maintain data integrity; (5) Low-complexity reads (containing 10 consecutive identical bases) were removed to reduce noise and improve data quality.

Species annotation: After obtaining OTU representative sequences, RDP classifier (v2.2) was used to align these sequences with reference databases for species annotation, with a confidence threshold of 0.6. For 16S rRNA (bacteria and archaea), the Greengene, RDP **^[19]^** , or Silva databases were used; for 18S rRNA (fungi), the Silva database was used; and for ITS (fungi), the UNITE database was used. Annotation results were filtered as follows:

(1) Excluding OTUs with no annotation results;

(2) Excluding OTUs annotated to species inconsistent with the analysis target (e.g., OTUs annotated as archaea);

(3) Retaining remaining OTUs for subsequent analyses.

Functional prediction: PICRUSt2 (v2.3.0-b) **^[20]^** was used to predict microbial community functional abundance based on marker gene sequences. Additionally, the KEGG database (http://www.genome.jp/kegg/) was used for pathway analysis to predict metabolic pathways and biological processes involved in the microbial community.

**1.2.4 Statistical Analysis of Metagenomic Data**

In the statistical analysis of metagenomic data in this study, multiple methods were employed to comprehensively explore the association between gut microbiota characteristics and CHF. Firstly, species accumulation curves were used to analyze the relationship between sampling size and species increase, thereby evaluating the sufficiency of sampling and estimating species richness. Meanwhile, mothur software (v.1.31.2) **^[21]^** was utilized to calculate Chao1, ACE, Shannon, and Simpson indices for assessing Alpha diversity, quantifying the microbiota characteristics within individual samples from dimensions such as species richness and evenness. QIIME software (v1.80) **^[22]^** was used to compute UniFrac distances between samples, combined with partial least squares discriminant analysis (PLS-DA) for inter-group classification, thus analyzing Beta diversity to reveal differences in species composition among different samples **^[23]^**. Additionally, LEfSe analysis was applied to test differences in species abundance between groups at six taxonomic levels (phylum, class, order, family, genus, and species) **^[24]^**, with significance evaluated using the false discovery rate (FDR). The Wilcoxon rank-sum test was used for comparisons between two groups to identify biomarkers.

Based on the above analysis results, this study constructed a gut microbiome model and a combined model of microbiota and clinical indicators for the diagnosis of CHF. Receiver Operating Characteristic (ROC) curve analysis was performed to evaluate the predictive accuracy of relevant variables, with the Area Under the Curve (AUC) value used to measure predictive performance **^[25]^**. Multivariate logistic regression analysis was conducted to identify independent risk factors for CHF, and a stability selection process with up to 100 repetitions was performed within the model framework to re-evaluate AUC scores, thereby confirming the reliability of biomarker characteristics **^[26]^**. In this process, high-quality metagenomic sequencing data played a crucial role; accurate gut microbiota data facilitated the precise identification of microbial features associated with CHF, enhancing the model's ability to distinguish between patients and healthy controls. Multiple repeated evaluations of AUC scores also improved the model's stability, providing strong support for the early diagnosis and risk assessment of CHF.

For data processing and statistical analysis, SPSS version 22.0 and R version 3.2.4 software were used. Continuous variables were expressed as mean ± standard deviation (mean ± SD), and categorical variables were presented as frequencies and percentages. The Shapiro-Wilk test was first used to assess data normality. For normally distributed continuous variables, the t-test was applied to evaluate differences between CHF patients and the control group; if the data were non-normally distributed, logarithmic transformation or non-parametric tests were used, such as the Mann-Whitney U test for two-group comparisons and the Kruskal-Wallis test for multiple -group comparisons. The chi-square test was used for the analysis of categorical data, with a statistically significant threshold set at *P* < 0.05.

**1.3 Non-Targeted Metabolomic Sequencing**

1.3 Non-Targeted Metabolomic Sequencing

Sample Processing and Quality Control:

To ensure the accuracy and reproducibility of the metabolomic analysis, all sample processing steps were conducted under strict sterile conditions to prevent contamination from exogenous sources. After serum separation, protein precipitation was immediately performed using methanol to stabilize the metabolites.

"Quality control was maintained through the following measures: (1) Use of calibrated blood collection equipment to ensure accurate sample volumes; (2) Filtration of supernatants with a 0.22 µm filter membrane to remove residual particulate impurities; (3) Regular insertion of quality control (QC) samples during the experiment to monitor experimental repeatability and stability."

Instrumental Parameters:

"Liquid Chromatography Conditions: An ACQUITY UPLC column (100 mm × 2.1 mm, 1.7 µm, Waters, UK) was used with a column temperature of 50°C and a flow rate of 0.4 mL/min. Mobile phase A was an aqueous solution containing 0.1% formic acid, and mobile phase B was a methanol solution containing 0.1% formic acid. The elution gradient was as follows: 0–2 min, 100% A; 2–11 min, 0–100% B; 11–13 min, 100% B; 13–15 min, 0–100% A."

Mass Spectrometry Conditions: A Xevo G2-XS QTOF mass spectrometer (Waters, UK) was used for detection in both positive and negative ion modes. For positive ion mode: capillary voltage was 3.0 kV, and cone voltage was 40.0 V; for negative ion mode: capillary voltage was 2.0 kV, and cone voltage was 40.0 V. In MSE mode, the primary scanning range was 50–1200 Da, with a scanning time of 0.2 s and collision energies of 20–40 eV. Mass calibration was performed via LE signal every 3 s, and pooled QC samples were inserted every 10 samples to monitor instrument stability.

Non-targeted metabolomics was used to analyze serum samples from the CHF group and the control group to screen for metabolic markers associated with CHF. This method enables comprehensive detection of small-molecule metabolites in samples without being restricted to specific metabolic pathways. The experimental workflow includes sample preparation, quality control (QC) sample preparation, metabolite extraction, instrumental detection, and data analysis.

**1.3.1 Sample Preparation (Pretreatment)**

The specific procedures are as follows:

(1) Sample collection: Calibrated blood collection equipment was used to collect blood samples from the CHF group and the control group in strict accordance with sterile operations to ensure standardization and consistency.

(2) Serum separation: Blood samples were allowed to stand at room temperature for 30-60 minutes to coagulate naturally, then centrifuged at 1000-2000 × g at 4°C for 10-20 minutes to separate serum.

(3) Serum transfer: A pipette was used to transfer the serum to a sterile tube, avoiding disturbance of the blood clot or contamination with impurities.

(4) Serum storage: The serum was aliquoted and stored at -80°C to ensure constant temperature for maintaining chemical stability.

(5) Serum thawing: Before the experiment, the serum was removed from -80°C and slowly thawed at room temperature.

(6) Serum mixing: The serum was gently oscillated to ensure uniform mixing.

(7) Protein precipitation: Methanol was added to precipitate proteins, followed by oscillation for mixing and centrifugation.

(8) Supernatant collection: The supernatant was aspirated and transferred to a new centrifuge tube, avoiding aspiration of the precipitate.

(9) Sample filtration: The supernatant was filtered using a 0.22 μm filter membrane to remove residual particulate impurities.

(10) Sample storage: The filtered samples were stored in a -20°C refrigerator until LC-MS/MS analysis.

**1.3.2 Preparation and Analysis of Quality Control Samples**

The preparation and analysis of quality control (QC) samples are critical steps for quality control in metabolomic studies, with the following procedures:

(1) Sample preparation: QC samples with biological and chemical properties similar to those of the experimental samples were prepared.

(2) Sample processing: QC samples underwent the same pretreatment steps as the experimental samples, including serum separation, storage, thawing, mixing, protein precipitation, and filtration.

(3) Sample mixing: Multiple QC samples were mixed to generate a representative pooled sample.

(4) QC sample analysis: QC samples were analyzed periodically during the experiment to monitor experimental variability and repeatability.

(5) QC sample storage: When necessary, QC samples were properly stored for subsequent analysis.

(6) QC sample monitoring: The stability and repeatability of the experiment were evaluated by comparing QC sample results at different time points or batches.

(7) QC sample data: QC sample data were collected and analyzed to assess the overall quality of the experiment.

The overlapping total ion current (TIC) plot of QC samples (Figure 1-1) showed a high degree of coincidence, indicating stable instrument status and good experimental repeatability. The plot was drawn with time as the abscissa and the sum of all ion intensities in the mass spectrum at each time point as the ordinate.

**
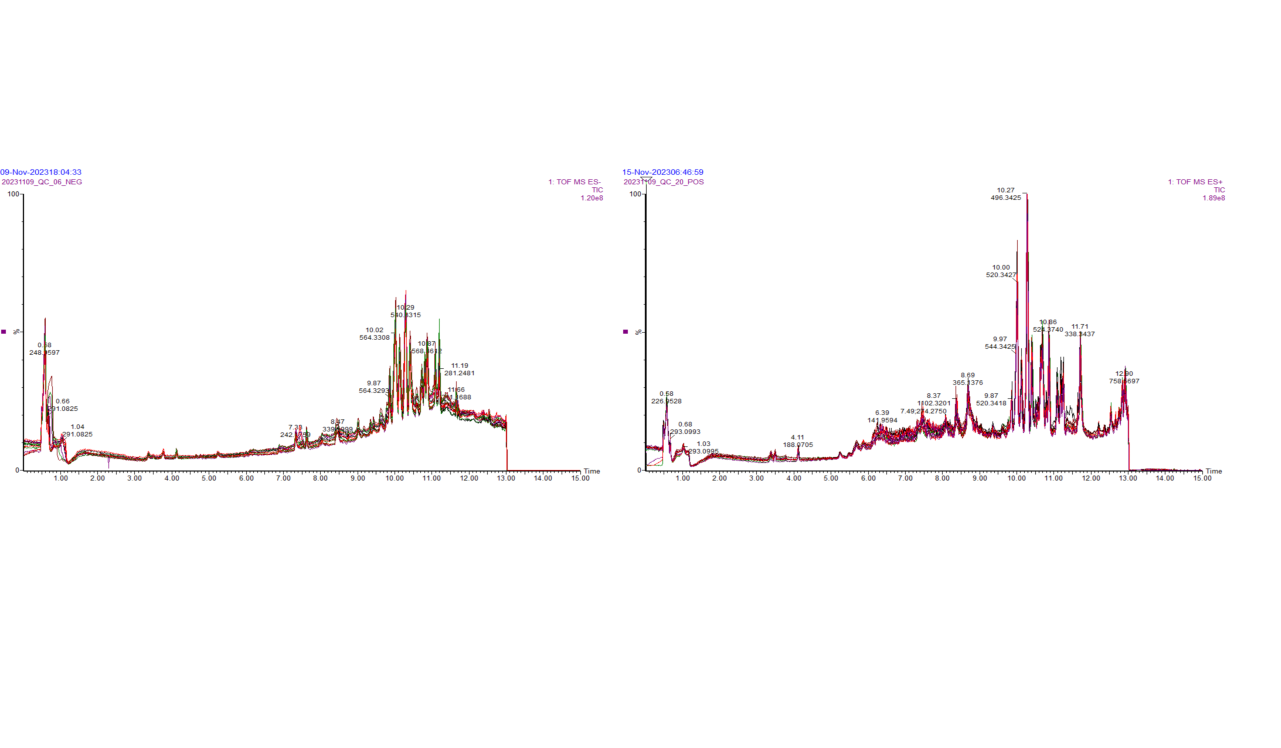
**

**Figure 1-1 QC sample positive and negative ion mode TIC overlapping pattern**

**1.3.3 Extraction of Metabolites**

The experiment utilized liquid chromatography (2777C UPLC system, Waters, UK) and mass spectrometry (Xevo G2-XS QTOF, Waters, UK). The procedures were as follows:

(1) 40 µL of sample was added to a 96-well plate;

(2) If the number of samples exceeded 50, one fetal bovine serum sample was inserted every 10 samples; if fewer than 50, no insertion was performed;

(3) 120 µL of pre-cooled methanol was added, the plate was sealed with a membrane and vortexed for 1 min, followed by precipitation at -20°C for 2 hours or overnight;

(4) Centrifugation was conducted at 4000 × g and 4°C for 30 min;

(5) 25 µL of each sample was transferred to a new 96-well plate, and 225 µL of 50% methanol was added for dilution;

(6) 50 µL of each sample was mixed to prepare quality control (QC) samples;

(7) 60 µL of supernatant was transferred to a 96-well microplate, sealed with a membrane, labeled, and then subjected to instrumental detection.

**1.3.4 Liquid Chromatography Conditions**

Chromatographic separation was performed using an ACQUITY UPLC column (100 mm × 2.1 mm, 1.7 μm, Waters, UK) with a column temperature of 50°C and a flow rate of 0.4 mL/min. Mobile phase A was an aqueous solution containing 0.1% formic acid, and mobile phase B was a methanol solution containing 0.1% formic acid. The elution gradient was as follows: 0–2 min, 100% A; 2–11 min, 0–100% B; 11–13 min, 100% B; 13–15 min, 0–100% A. The injection volume was 5 µL.

**1.3.5 Mass Spectrometry Conditions**

A Xevo G2-XS QTOF mass spectrometer (Waters, UK) was used for detection in both positive and negative ion modes. For positive ion mode: capillary voltage was 3.0 kV, and cone voltage was 40.0 V; for negative ion mode: capillary voltage was 2.0 kV, and cone voltage was 40.0 V. In MSE mode, the primary scanning range was 50–1200 Da, with a scanning time of 0.2 s and collision energies of 20–40 eV. Mass calibration was performed via LE signal every 3 s, and pooled QC samples were inserted every 10 samples to monitor instrument stability.

**1.3.6 Peak Extraction and Identification**

Progenesis QI software (v2.2) was used for peak extraction, alignment, normalization, and compound identification.

**1.3.7 QC-RLSC**

Quality control–based robust LOESS signal correction (QC-RLSC), which performs local polynomial regression fitting for signal correction of real sample signals based on QC sample information, is an effective data correction method in omics data analysis within the field of metabolomics **^[27]^** .

**1.3.8 PLS-DA Analysis**

PLS-DA analysis is a widely used multivariate statistical analysis method in metabolomics. In the PLS-DA model, the parameter R² (R²Y) represents the explanatory rate of the model, and Q² (Q²Y) represents the predictive rate. Theoretically, the closer R² and Q² are to 1, the better the model. Generally, a Q² value higher than 0.5 indicates good predictive performance of the model. In this study, prior to constructing the PLS-DA model, log2 transformation and Pareto scaling were applied for data scaling **^[28]^**.

**1.3.9 Statistical Analysis of Metabolomic Data**

The statistical analysis of metabolomic data in this study aimed to explore the association between metabolites and CHF. Raw data were converted to .mzXML format using ProteoWizard, and XCMS was used for peak alignment, retention time correction, and peak area extraction. Metabolites were identified based on accurate mass, secondary spectrum matching, and database retrieval. Subsequently, the data were preprocessed with Pareto scaling, and functional annotation was performed using the KEGG database.

For screening differential metabolites and constructing diagnostic models, the OPLS-DA model was used to calculate Variable Importance for the Projection (VIP) values to evaluate the impact of metabolites on sample classification. Significantly differential metabolites were screened using the criteria of VIP > 1 and *P* < 0.05 **^[29-30]^**. A metabolite diagnostic model was constructed, and ROC curve analysis was used to evaluate predictive accuracy, with AUC values measuring performance. Multivariate logistic regression was used to identify independent risk metabolites for CHF, and AUC scores were evaluated through multiple stability selection processes to confirm the reliability of biomarkers.

For data processing and statistics, SPSS version 22.0 and R version 3.2.4 software were used. The Shapiro-Wilk test was first used to assess data normality. For normally distributed continuous variables, the *t*-test was used to compare differences between groups; for non-normal data, logarithmic transformation or non-parametric tests were applied (e.g., Mann-Whitney U test for two groups, Kruskal-Wallis test for multiple groups). Categorical variables were analyzed using the chi-square test, with a significance threshold set at *P* < 0.05 to ensure the scientific reliability of the study results.

***Part 2***

**1 Research Content and Methods**

**1.1 Research Materials**

**1.1.1 Experimental Cells**

The rat cardiomyocyte line H9C2 used in this study was provided by Procell (Wuhan, China), with the cell catalog number CL-0089.

**1.1.2 Experimental Bacteria and Culture Media**

*Segatella* bacteria used in this study were purchased from Beina Biotechnology Co., Ltd. (Beijing, China), with the strain number BNCC354512; Doxorubicin (Dox) (Macklin, catalog number: D807083-500mg), regular culture medium, and low-glucose medium (used for LCD intervention) were also employed.

Low-glucose DMEM medium (Procell, PM150220) with 20%, 50%, and 100% concentrations refers to the proportion of low-glucose medium in the cell culture medium:

(1) For 6-well plates containing 2 mL of medium: 1) 20% refers to 0.4 mL low-glucose medium + 1.6 mL high-glucose medium; 2) 50% refers to 1 mL low-glucose medium + 1 mL high-glucose medium; 3) 100% refers to 2 mL low-glucose medium.

(2) For 96-well plates containing 100 µL of medium: 1) 20% refers to 20 µL low-glucose medium + 80 µL high-glucose medium; 2) 50% refers to 50 µL low-glucose medium + 50 µL high-glucose medium; 3) 100% refers to 100 µL low-glucose medium.

**1.2 Reagents, Consumables, and Instruments**

**1.2.1 Main Instruments**

(1) Grinder (Beijing Hede, N.9548)

(2) CO₂ incubator (YILANG, YCP-1000)

(3) Liquid nitrogen tank (Dongya, YDS-35L-80)

(4) Electric constant-temperature water bath (Beijing 328 Science, HH.S11-Ni2)

(5) Multifunctional microplate reader (Molecular Devices, SpectraMax-M5)

(6) Flow cytometer (BENM DIAG, BeamCyte-1026)

(7) Refrigerated centrifuge (LABGIC, China, CF1524R)

(8) Micro-spectrophotometer (Youmi, Unano-1000)

(9) Quantitative real-time PCR instrument (GE nesd, e-1000)

(10) Gel imaging system (Biorad, 2500)

(11) Desktop high-speed refrigerated centrifuge (LABGIC, CF1524R)

(12) Spectrophotometer (Youmi Instruments, Unano-1000)

(13) Electric constant-temperature water bath (Shanghai Yiheng, Constant Temperature Equipment Factory, HWS-24)

(14) NC membrane (0.22 µm) (GVS, USA, 1215458)

(15) 3MM filter paper (Whatman, UK, 3030861)

(16) Decolorizing shaker (Haimen Qilinbeier Instrument Manufacturing Co., Ltd., TS-100)

(17) Electrophoresis apparatus (Beijing Baijing Biotechnology Co., Ltd., BG-subMIDI)

(18) Gel imaging system (UVP, USA, GelDoc-It310)

(19) Chemiluminescence imaging system (CLINX, China, ChemiScope6100)

(20) Transfer tank (Junyi, Trans-blot)

**1.2.2 Main Reagents and Consumables**

(1) 6-well plates (Thermo, 140675)

(2) Cell apoptosis detection kit (Sibcbio, FXP018)

(3) Reactive oxygen species detection kit (Beyotime, S0033S)

(4) Protease inhibitor (MDL, MD912893)

(5) BCA protein concentration assay kit (MDL, MD913053)

(6) SDS-PAGE precast gel kit (MDL, MD911919)

(7) Protein molecular weight marker (Zhong Sheng Ao Bang, 02.15005)

(8) Secondary antibody (MDL, MD912565)

(9) β-actin (Affinity, AF7018)

(10) TRIZOL (Aidlab, RN0102)

(11) Anhydrous ethanol (Macklin, E809056)

(12) Isopropanol (Macklin, I811925)

(13) UltraPure Agarose (Bomad, SH441-01)

(14) SuperScript III RT reverse transcription kit (EXONGEN, A502)

(15) Dox (Macklin, D807083-500mg)

(16) Complete medium (Procell, PM150210)

(17) Fetal Bovine Serum (FBS) (Procell, 164210-50)

(18) Penicillin-streptomycin solution (Procell, PB180120)

(19) Columbia blood agar plates (Beina Biology)

(20) Goat anti-rabbit secondary antibody (Luoyang BioTone Experimental Materials Center, C030212)

**1.3 Experimental Methods**

**1.3.1 Cell Culture**

**1.3.1.1 Resuscitation of Rat Cardiomyocytes H9C2 (2-1)**

(1) Preheat a constant-temperature water bath to 37°C, and add 5 mL of complete medium containing 10% fetal bovine serum (FBS) to a 15 mL centrifuge tube, then place it in the water bath for preheating.

(2) Wear goggles and thick gloves, quickly take out the cell cryopreservation tube from the liquid nitrogen tank, immediately transfer it to the 37°C water bath for rewarming, and gently shake to accelerate thawing.

(3) After the cells are completely thawed, transfer the cell suspension in the cryopreservation tube to the preheated centrifuge tube, mix well, and centrifuge at 1000 rpm for 5 min.

(4) Prepare a T-25 culture flask, label it with the cell name and date, and add 4 mL of complete medium.

(5) After centrifugation, discard the supernatant, resuspend the cell pellet with 1 mL of complete medium, transfer the suspension to the T-25 culture flask, mix well, and incubate in a 37°C, 5% CO₂ incubator.

**1.3.1.2 Passage of Rat Cardiomyocytes H9C2 (2-1)**

(1) When the cell confluency reaches over 85%, collect the medium in the culture flask in a biological safety cabinet.

(2) Add 3 mL of sterile 1× PBS to the culture flask, place it horizontally to allow PBS to evenly the bottom surface, then aspirate and discard the PBS.

(3) Add 1 mL of digestive solution to the bottom surface, and incubate the culture flask in a 37°C, 5% CO₂ incubator for 1-2 min.

(4) After incubation, observe under an inverted microscope; if the cells become rounded and float, add 2 mL of complete medium containing 10% FBS to terminate digestion, and transfer the cell suspension to a 15 mL centrifuge tube.

(5) Centrifuge at 1000 rpm for 5 min, and prepare two T-25 culture flasks with 4 mL of complete medium added to each.

(6) After centrifugation, discard the supernatant, resuspend the cells with 2 mL of complete medium, and evenly distribute the suspension into the two T-25 culture flasks, adding 1 mL of cell suspension to each flask.

(7) Place the culture flasks horizontally, gently shake to mix well, and incubate in a 37°C, 5% CO₂ incubator.

**1.3.2 Culture of *Segatella* Bacteria**

**1.3.2.1 Bacterial Resuscitation**

Bacterial morphology: Colonies are 1-2 mm in diameter, round with neat edges, opaque, grayish-white on the front, convex in the middle, smooth, bright, and moist in texture; Gram-negative (red), bacilli, and pure in purity.

(1) Prepare 1-2 Columbia blood agar plates, and deoxygenate them anaerobically for 24 h to create an anaerobic environment.

(2) Disinfect the surface of the plates, and open the plates in a biological safety cabinet.

(3) Pipette 2 mL of sterile water into the plate, scrape the bacterial lawn with a sterile spreader, and stir to prepare a uniform bacterial suspension.

(4) Pipette an appropriate amount of bacterial suspension and drop it onto a fresh plate, then spread evenly.

(5) Incubate the plate in a 37°C strict anaerobic incubator for 48-72 h to ensure stable anaerobic conditions.

**1.3.2.2 Bacterial Passage**

(1) Prepare Columbia liquid medium under strict aseptic conditions to ensure sterility.

(2) Prepare well-preserved Columbia blood agar plates of *Segatella* bacteria, ensuring no dryness or contamination.

(3) Pick typical, well-grown single colonies with a sterile pipette tip or disinfected toothpick.

(4) Transfer the colonies to the liquid medium, and gently vortex to disperse the colonies.

(5) Cover the culture flask with sterile aluminum foil or a non-airtight lid to ensure ventilation and prevent contamination.

(6) Incubate the culture flask in a 37°C anaerobic incubator with appropriate shaking for 12-18 hours.

(7) After incubation, evaluate bacterial growth by the turbidity of the medium, and identify contamination by microscopy or other methods if necessary.

(8) The concentration of the bacterial solution used is 3.75×10⁸ CFU/mL, which is accurately determined by the plate colony counting method.

**1.3.3 Preliminary Experimental Methods**

A CCK-8 reagent (brand: Fluorescence, cat: F25-10ml, Lot: DCM2223) was used in preliminary experiments to determine key parameters for subsequent formal experiments.

**1.3.3.1 Screening of *Segatella* Bacterial Concentration and Stimulation Time**

Multiplicity of Infection (MOI) was set as 0, 0.1, 1, and 10, where MOI=0 served as the negative control group (normal cell culture without bacterial solution). Cells were stimulated for 24 hours respectively. After stimulation, cell viability was detected using the CCK-8 reagent, and the cell culture supernatant was collected to measure lactate dehydrogenase (LDH) release. A comprehensive evaluation was performed to determine the appropriate concentration range and action time of *Segatella* bacteria affecting cardiomyocyte viability, providing a basis for setting bacterial concentrations in formal experiments. During the operation, precise volumes of *Segatella* bacterial solution corresponding to different MOIs were added to the cell culture system.

**1.3.3.2 Optimization of Conditions for Constructing Cardiomyocyte Injury Model**

The damaging effects of doxorubicin (Dox) at different concentrations (0, 1, 2, 4 μM) and action times (24 h, 48 h, 72 h) on cardiomyocytes were investigated. Dox was accurately weighed and prepared into solutions of corresponding concentrations, which were then added to the cell culture system. After treatment at each time point and concentration, cell viability was detected using the CCK-8 reagent, and cells were collected to measure cardiac troponin I (cTnI) content. This was to determine the optimal combination of Dox concentration and action duration for inducing a stable cardiomyocyte injury model, ensuring the effectiveness and reliability of model construction in formal experiments.

**1.3.3.3 Exploration of Synergistic Effects between LCD Intervention and *Segatella* Bacteria**

Based on the cardiomyocyte injury model induced by 1 μM Dox for 24 hours, LCD intervention ratios were set as 20%, 50%, and 100% (representing the reduction ratio of glucose content in the medium, i.e., replacement of high-glucose medium with low-glucose medium). Meanwhile, *Segatella* bacterial treatment groups with different MOI values (0.1, 1, 10) were set. Through multi-factor combination analysis, the interactive effects of LCD and *Segatella* bacteria on cardiomyocyte injury were preliminarily evaluated. This provided key references for precisely designing LCD intervention intensity and reasonably setting bacterial treatment groups in formal experiments, ensuring that formal experiments could efficiently and accurately explore the improvement effect of LCD intervention on *Segatella*-induced cardiomyocyte injury and its underlying mechanisms.

**1.3.4 Formal Experimental Methods**

**1.3.4.1 Experimental Grouping**

Each 6-well plate was seeded with 1×10⁶ cells.

(1) Normal control group (Control Group): Cardiomyocytes in this group were cultured normally without any additional special treatments. As the baseline control for the entire experiment, it served to provide a reference standard for subsequent evaluations of the extent and direction of effects of different treatment factors on various cardiomyocyte indices (e.g., cell viability, apoptosis rate, related protein expression).

(2) Dox group: Cardiomyocytes were treated with 1 µM Dox for 24 h to construct a stable myocardial injury model, simulating the pathophysiological changes of cardiomyocytes in heart failure. This model provided a basic pathological basis for studying the effects of *Segatella* bacteria and LCD intervention.

(3) Dox + low-concentration *Segatella* group (Dox + *Segatella*·L): After constructing the myocardial injury model, *Segatella* culture with an MOI of 0.1 (1 µL of bacterial solution at a concentration of 3.75×10⁸ CFU/mL, determined based on preliminary experimental results) was added, and culture was continued for 24 h. This group aimed to explore the direct impact of low-concentration *Segatella* on injured cardiomyocytes, potential involvement in injury mechanisms, and clarify the characteristics and patterns of the bacteria’s role in the process of cardiomyocyte injury.

(4) Dox + high-concentration *Segatella* group (Dox + *Segatella*·H): After constructing the myocardial injury model, *Segatella* culture with an MOI of 1 (10 µL of bacterial solution at a concentration of 3.75×10⁸ CFU/mL, determined based on preliminary experimental results) was added, and culture was continued for 24 h. Compared with the low-concentration group, this group further analyzed the dose-effect relationship of different *Segatella* concentrations on the degree of cardiomyocyte injury and related cellular biological processes.

(5) Dox + low-concentration *Segatella* + 20% LCD group (Dox + *Segatella*·L + LCD): After constructing the myocardial injury model, the medium was replaced with 20% low-glucose medium, and *Segatella* culture with an MOI of 0.1 was added, followed by 24 h of culture. This group aimed to investigate the ability of LCD to improve cardiomyocyte function in the presence of low-concentration *Segatella* and potential synergistic mechanisms.

(6) Dox + high-concentration *Segatella* + 20% LCD group (Dox + *Segatella*·H + LCD): After constructing the myocardial injury model, the medium was replaced with 20% low-glucose medium, and *Segatella* culture with an MOI of 1 was added, followed by 24 h of culture. This group evaluated the repair of cardiomyocyte injury under the combined action of high-concentration *Segatella* and LCD.

**1.3.4.2 Detection of Cell Apoptosis (Annexin V-FITC/PI Double Staining)**

(1) Preparation of cell samples:

① Cell digestion and collection: Discard the medium, digest adherent cells with 0.25% trypsin (without EDTA), add complete medium to terminate digestion, gently pipette to form a single-cell suspension, and transfer to a centrifuge tube.

② Centrifugation and washing: Centrifuge at 300×g for 5 min at 4°C, discard the supernatant, and resuspend and wash once with pre-cooled PBS.

③ Buffer preparation: Dilute 4×Annexin V binding buffer with PBS to 1× (final concentration: 10 mM HEPES/NaOH, 140 mM NaCl, 2.5 mM CaCl₂, pH 7.4).

④ Cell resuspension: Adjust the cell density to 1×10⁶ cells/mL with 1× binding buffer.

(2) Staining and flow cytometry detection:

① Annexin V-FITC staining: Take 100 μL of cell suspension, add 5 μL of Annexin V-FITC, and incubate at room temperature in the dark for 10 min.

② PI staining: Add 10 μL of PI staining solution (20 μg/mL) and 400 μL of PBS, mix well, and immediately load onto the machine for detection.

③ Flow cytometry parameters: FITC (Ex/Em = 488/530 nm), PI (Ex/Em = 488/617 nm).

**1.3.4.3 Detection of ROS Levels (DCFH-DA Fluorescent Probe Method)**

(1) Probe loading: Dilute DCFH-DA with serum-free medium to a final concentration of 10 μM. Discard the medium, add the probe working solution to cover the cells, incubate at 37°C in the dark for 20 min, and wash 3 times with serum-free medium.

(2) Setting of positive control: Add ROS inducer to the positive control group and co-incubate for 4 h.

(3) Flow cytometry detection: After trypsin digestion, centrifuge at 300×g for 5 min, resuspend in PBS to a density of 1×10⁶ cells/mL. Detection parameters: FITC channel (Ex/Em = 488/525 nm), 10,000 cells detected, and mean fluorescence intensity (MFI) analyzed using FlowJo software.

**1.3.4.4 qPCR Detection**

In this study, qPCR was used to detect the expression levels of apoptosis-related genes (Bax, Bcl-2), oxidative stress-related genes (SOD1, CAT), and genes in the TLR4/MyD88/NF-κB signaling pathway (TLR4, MyD88, NF-κB p65). This aimed to systematically analyze the regulatory mechanisms of *Segatella* and LCD intervention on the gene expression network in cardiomyocytes. The experiment strictly followed standard procedures, including key steps such as RNA extraction, primer design and validation, reverse transcription, and qPCR amplification.

(1) Extraction of total RNA from cell samples:

① Sample pretreatment: Discard the medium, wash H9C2 cells twice with pre-cooled PBS, add 1 mL of Trizol to lyse the cells, and let stand on ice for 5 min.

② Chloroform extraction: Add 200 µL of chloroform, vortex for 15 s, and centrifuge at 12,000 rpm for 15 min at 4°C.

③ RNA precipitation: Transfer the upper aqueous phase to a new tube, add an equal volume of isopropanol, precipitate at -20°C for 30 min, and centrifuge at 12,000 rpm for 10 min at 4°C.

④ Washing and dissolution: Wash the precipitate twice with 75% ethanol, air-dry, and dissolve in 30 μL of DEPC-treated water stored at -80°C.

(2) Quality detection of total RNA:

① Concentration and purity: Nanodrop 2000c was used to detect the A260/A280 ratio (1.8–2.1 was considered qualified) and A260/A230 ratio (>2.0).

② Integrity verification: 1% agarose gel electrophoresis (80 V, 30 min) showed clear 28S/18S rRNA bands (the brightness of 28S was approximately twice that of 18S).

(3) Primer design and validation:

① Primer design: Primer Premier 6.0 software was used to design primers for target genes (e.g., Actin, Bax, Bcl-2, SOD1, Cat, TLR4, MyD88, NF-κB p65) (see Table 2-1). The software was used to simulate annealing temperatures to screen for primer pairs with high specificity and efficient amplification.

② Primer validation: Specificity was confirmed by NCBI Primer-BLAST to ensure no off-target amplification; amplification efficiency was verified by the standard curve method (5-fold serial dilution of cDNA templates) with an efficiency of 90%–110%; melting curves showed a single peak indicating specific amplification (double peaks suggested primer dimers requiring optimization).

**Table 2-1 Primers used in this work**

| Target Name | Primer | Primer Sequence（5'→3'） | Product length | Annealing temperature |
| --- | --- | --- | --- | --- |
| Actin | F | ACCCAGATCATGTTTGAGACCT | 150 bp | 58°C |
|  | R | GACCAGAGGCATACAGGGACAAC |  |  |
| Bax | F | GATCGAGCAGAGAGGATGGC | 121 bp | 58°C |
|  | R | TGTTGTCCAGTTCATCGCCA |  |  |
| BCL-2 | F | ACTCTTCAGGGATGGGGGTGA | 117 bp | 58°C |
|  | R | AGAGCGATGTTGTCCACCAG |  |  |
| SODI | F | AGGGCGTCATTCACTTCGAG | 89 bp | 58°C |
|  | R | CCCATGCTCGCCTTCAGTTA |  |  |
| Cat | F | TGAAGCAGTGGCAAGGAGCAG | 152 bp | 58°C |
|  | R | TGCCATCTCGTCGGTGAAAA |  |  |
| TLR4 | F | GTAGAAATGCCATGAGCTT | 152 bp | 58°C |
|  | R | TATTCCAGCTCTTCTAGACC |  |  |
| MyD88 | F | GTTGCTAGCCTTGTTAGACCGTG | 198 bp | 58°C |
|  | R | CTCCTGTTTCTGCTGGTTGCG |  |  |
| NF-κB p65 | F | GGGATGGCTTCTATGAGGCT | 185 bp | 60°C |
|  | R | GGTCTCGCTTCTTCACACAC |  |  |

1. Reverse Transcription for cDNA Synthesis

① The reaction system was constructed using the ExonScript RT SuperMix with dsDNase reverse transcription kit (EXONGEN) as shown in Table 2-2 below:

**Table 2-2 Reverse Transcription Reaction System**

| Component | Volume | Final Concentration |
| --- | --- | --- |
| RNA（1-2µg） | 4µL | - |
| 5× Reaction Buffer | 4µL | 1× |
| dNTP Mix（10mM each） | 2µL | 0.5mM each |
| Random Hexamer Primer（50 µM） | 1µL | 2.5µM |
| Reverse Transcriptase（200U/µL） | 1µL | 200U |
| RNase Inhibitor（40U/µL） | 0.5µL | 20U |
| DEPC-treated Water | 7.5µL | - |
| Total Volume | 20µL | - |

② Reverse transcription reaction program settings: The reaction system was mixed thoroughly, briefly centrifuged, and then placed in a PCR instrument to run according to the program in Table 2-3. After the reaction, the cDNA products were immediately stored at -20°C to avoid repeated freeze-thaw cycles, ensuring their stability and integrity for subsequent qPCR detection.

(5) Real-time fluorescent quantitative detection

① Establishment of reaction system: The qPCR reaction system was prepared in nuclease-free PCR tubes according to Table 2-4, mixed thoroughly, and briefly centrifuged to ensure uniform distribution of components without air bubbles.

**Table 2-3 Reverse transcription program**

| Step | Temperature | Time |
| --- | --- | --- |
| Primer Annealing | 25°C | 10min |
| Reverse Transcription | 55°C | 30min |
| Enzyme Inactivation | 85°C | 5min |

**Table 2-4 qPCR reaction system**

| Component | Volume |
| --- | --- |
| cDNA Template | 1µL |
| 2×qPCR Master Mix | 10µL |
| Forward Primer（10µM） | 0.4µL |
| Reverse Primer（10µM） | 0.4µL |
| ddH_2_O | 8.2µL |
| Total Volume | 20µL |

② Reaction condition settings: The reaction tubes were placed in a qPCR instrument, and the reaction conditions were set according to Table 2-5. Fluorescent signals were monitored in real-time, and Ct values were collected for gene expression analysis. Melting curve analysis was performed to verify product specificity: a single sharp peak indicated high product specificity, while multiple peaks or broad peaks suggested primer dimers or non-specific amplification, which required optimization of primers or reaction conditions to ensure the accuracy and reliability of the results.

**Table 2-5 qPCR reaction conditions**

| Step | Temperature | Time | Cycles |
| --- | --- | --- | --- |
| Initial Denaturation | 95°C | 5min | 1 |
| Denaturation | 95°C | 10sec | 40 |
| Annealing | 58°C | 20sec | 40 |
| Extension | 72°C | 20sec | 40 |
| Melting Curve Analysis | 95°C | 15sec | 1 |
|  | 60°C | 60°C | 1 |
|  | 95°C | 15sec | 1 |

**1.3.4.5 Western Blot Analysis**

In this study, Western Blot (WB) was used to detect the expression of key proteins (cleaved caspase-3, p53, TLR4, MyD88, nuclear NF-κB p65, etc.), aiming to clarify the regulatory mechanisms of *Segatella* and LCD intervention on the protein expression profile and signaling pathways in injured cardiomyocytes. The experiment strictly followed standard WB procedures, including cell lysis, protein quantification, SDS-PAGE electrophoresis, membrane transfer, blocking, antibody incubation (primary and secondary antibodies), and chemiluminescent visualization.

(1) Extraction of Cellular Proteins

① Total protein extraction (for detection of cleaved caspase-3, p53, TLR4, MyD88): The medium was discarded, and cells were washed twice with pre-cooled PBS. Cells were scraped into 1.5 mL centrifuge tubes. RIPA lysis buffer containing 1% PMSF was added, and cells were lysed on ice for 30 min. Ultrasonication was performed on ice (20% power, 10 seconds per cycle × 3 cycles, 10-second intervals). After centrifugation at 12,000 rpm for 15 min at 4°C, the supernatant (total protein) was collected and stored at -80°C.

② Nuclear protein extraction (for detection of nuclear NF-κB p65): Cell collection was performed as described for total protein extraction. Cytoplasmic/nuclear separation was conducted according to the kit instructions: cytoplasmic lysis buffer was added, and cells were lysed on ice for 15 min, followed by centrifugation at 500×g for 5 min. The supernatant was cytoplasmic protein; the pellet was lysed with nuclear lysis buffer on ice for 30 min, then centrifuged at 12,000 rpm for 10 min, and the supernatant (nuclear protein) was collected.

(2) Protein Concentration Determination (BCA Method)

① BSA standard solutions (0–1.6 mg/mL) were diluted.

② Sample dilution: Total protein was diluted 10-fold, and nuclear protein was diluted 5-fold.

③ 20 µL of sample/standard + 200 µL of BCA working solution was added to each well, followed by incubation at 37°C for 30 min.

④ Absorbance at 562 nm was measured using a microplate reader, and protein concentrations were calculated.

(3) SDS-PAGE Electrophoresis

① Separation gel preparation: The formulation was prepared according to Table 2-6, overlaid with deionized water, and allowed to stand at room temperature for 40 min until solidified.

② Stacking gel preparation: Deionized water was poured off, and residual liquid was aspirated. The gel plate was placed vertically, and 4 mL of 5% stacking gel was added (avoiding bubbles). A sample comb was inserted, and the gel was allowed to stand at room temperature for 40 min until solidified.

③ Electrophoresis tank installation: The sample comb was removed, and the glass (with the "concave" side inward) was fixed in the electrophoresis tank.

④ Electrophoresis conditions: Constant voltage of 80 V for the stacking gel (approximately 20 min); constant voltage of 120 V for the separation gel (time adjusted according to protein molecular weight to ensure sufficient protein separation).

(4) Wet Transfer and Conditions

① Cleaved Caspase-3: Constant voltage of 100 V for 1 h at 4°C.

② p53: Constant voltage of 100 V for 1 h at 4°C.

③ TLR4: Constant voltage of 100 V for 1.5 h at 4°C.

④ MyD88: Constant voltage of 100 V for 1 h at 4°C.

⑤ Nuclear NF-κB p65: Constant voltage of 100 V for 1 h; PVDF membrane activated with methanol.

⑥ Transfer buffer: 25 mM Tris-base, 192 mM glycine, 20% methanol (pH 8.3).

**Table 2-6 SDS-PAGE gel preparation**

| Target Protein | Concentration of Separating Gel | Loading Amount |
| --- | --- | --- |
| Cleaved Caspase-3（17/19kDa） | 15% | 30μg |
| p53（53kDa） | 10% | 30μg |
| TLR4（95kDa） | 10% | 30μg |
| MyD88（33kDa） | 12% | 30μg |
| NF-κB p65（65kDa） | 12% | 15μg |

(5) Antibody Incubation and Color Development

① Blocking: 5% non - fat milk powder in TBST, at room temperature for 1 h.

② Primary antibody incubation: Dilute the primary antibodies according to the instructions, as detailed in Table 2 - 7.

③ Secondary antibody incubation: HRP - goat anti - rabbit/mouse IgG (1:5000), at room temperature for 1 h.

④ ECL development: Mix ECL A and B solutions at a ratio of 1:1 and evenly cover the membrane surface. Acquire signals using a chemiluminescence imaging system (exposure time: 10 s - 5 min).

⑤ Internal reference normalization: For total protein: Gray value of target protein / Gray value of β - actin; For nuclear protein: Gray value of nuclear NF - κB p65 / Gray value of Lamin B1.

**Table 2-7 Table of Primary Antibody Incubation Conditions**

| Antibody | Dilution Ratio | Buffer | Incubation Condition |
| --- | --- | --- | --- |
| Cleaved Caspase-3 | 1:1000 | 5% BSA/TBST | 4°C overnight |
| p53 | 1:500 | 5% Skim Milk/TBST | 4°C overnight |
| TLR4 | 1:1000 | 5% BSA/TBST | 4°C overnight |
| MyD88 | 1:800 | 5% Skim Milk/TBST | 4°C overnight |
| Nuclear NF-κB p65 | 1:800 | 5% Skim Milk/TBST | 4°C overnight |
| β-actin | 1:5000 | 5% Skim Milk/TBST | Room temperature for 2 h |

**1.3.4.6 ELISA Detection**

ELISA kits were used to detect the levels of inflammatory factors (TNF-α, IL-6, etc.) in cell culture supernatants, aiming to evaluate the regulatory effects of *Segatella* and LCD intervention on the inflammatory response and microenvironment of cardiomyocytes. The operation was strictly performed according to the kit instructions, with the following steps:

(1) Take out the required strip, and store the remaining strips at 4°C after sealing.

(2) Set standard wells and sample wells; add 50 µL of standard solutions with different concentrations to the standard wells.

(3) Add 10 µL of the sample to be tested and 40 µL of diluent to the sample wells; add nothing to the blank well.

(4) Except for the blank well, add 100 µL of HRP-labeled detection antibody to each well, seal the plate, and incubate at 37°C for 60 min.

(5) Discard the liquid, pat dry, fill each well with washing solution, let stand for 1 min, pat dry, and repeat this step 5 times.

(6) Add 50 µL of substrate A and 50 µL of substrate B to each well, and incubate at 37°C in the dark for 15 min.

(7) Add 50 µL of stop solution to each well, and measure the OD value at 450 nm within 15 min.

**1.3.5 Statistical Analysis**

SPSS 22.0 software was used for statistical analysis in this study. Measurement data were expressed as mean ± standard error (x ± SE). Normality and homogeneity of variance tests were performed before analysis. If the conditions were met, one-way ANOVA with LSD test was used; otherwise, rank sum test was applied, with the significance level set at α = 0.05. Meanwhile, GraphPad Prism 10.0 software was used to draw charts, which intuitively displayed data characteristics and differences between groups, ensuring the scientificity and reliability of the research results. One-way ANOVA was used for comparisons among multiple groups. "ns" indicates no significant difference, "*" indicates significance, with * representing *P* < 0.05, ** representing *P* < 0.01, *** representing *P* < 0.001, and so on.

***Part 3***

**1 Research Content and Methods**

**1.1 Experimental Materials**

**1.1.1 Experimental Animals**

Healthy SPF-grade Wistar rats were used as experimental subjects in this study, with a body weight of approximately 300 g, half male and half female, and aged 8-12 weeks. The experimental animals were provided by Sibeifu (Beijing) Biotechnology Co., Ltd., with the license number SCXK (Jing) 2024-0001. The experimental protocol has been approved by the Animal Ethics Committee of the First Affiliated Hospital of Xinjiang Medical University (approval number: K202402-05). Strict adherence to animal ethics standards was maintained to fully ensure animal welfare.

Considering the possible death of animals during the experiment, to ensure that 6 rats in each group were ultimately available for valid analysis, a certain number of backup rats were reserved in the initial allocation of each group. Specifically, at the start of the experiment, 8 rats were actually included in each group for rearing and observation. During the rearing process, the breeding environment was strictly controlled. The experimental rats were housed in an individually ventilated cage (IVC) system, which provided a stable environment with temperature (22±2°C), humidity (50±5%), and maintained air cleanliness, effectively reducing contamination from external microorganisms. Each cage housed 3-4 rats to avoid overcrowding. Meanwhile, to prevent cagemate rats from eating each other's feces and affecting the experimental results, a specially designed cage with a bottom grid was used, allowing feces to fall off in a timely manner and reducing the chance of rats coming into contact with other individuals' feces. After gavaging the rats, their behavior was closely observed to ensure that they did not ingest feces from other rats. If any abnormalities were found, the data of the relevant rats were promptly marked or excluded. In addition, bedding was changed regularly every day to maintain a clean breeding environment, further reducing experimental errors caused by fecal contamination.

**1.1.2 Experimental Bacteria**

Same as in Part 2

**1.1.3 Experimental Diets**

Maintenance diet was purchased from Shuyu Biology, catalog number SY1001; LCD diet was customized by Shuyu Biology. The nutritional composition of the normal diet meets the nutritional requirements of Wistar rats, with 65% carbohydrates, 20% protein, 8% fat, and is rich in various vitamins and minerals. The LCD diet ratio is 20% carbohydrates, 60% fat, and 20% protein, with the specific formula shown in Table 3-1 below:

**Table 3-1 Low-Carbohydrate Feed Formulation**

| Raw Materials | Gram Weight | Energy Supply Value |
| --- | --- | --- |
| Casein (Casein) | 200 | 800 |
| L-Cystine (L-Cystine) | 3 | 12 |
| Corn Starch (Corn Starch) | 0 | 0 |
| Maltodextrin (Maltodextrin) | 125 | 500 |
| Sucrose (Sucrose) | 72.8 | 275 |
| Cellulose (Cellulose) | 50 | 0 |
| Soybean Oil (Soybean Oil) | 25 | 225 |
| Lard (Lard) | 245 | 2205 |
| Mineral Mix S10026B (Mixed Minerals S10026B) | 50 | 0 |
| Vitamin Mix V10001C (Mixed Vitamins V10001C) | 1 | 4 |
| Choline Bitartrate (Choline Bitartrate) | 2 | 0 |
| FD&C Yellow Dye #5 | 0 | 0 |
| FD&C Red Dye #40 | 0 | 0 |
| FD&C Blue Dye #1 | 0.05 | 0 |
| Total | 773.85 | 4021 |

**1.2 Experimental Reagents, Consumables, and Main Instruments**

**1.2.1 Main Instruments**

(1) Electronic balance (Yingheng Electronic Technology Co., Ltd., Huizhou, YH-3)

(2) Biological signal acquisition and processing system (Zhongshi Dichuang Technology Development Co., Ltd., Beijing, MadLab-4C/501H)

(3) Animal anesthesia machine (Sendi Hengsheng Technology Development Co., Ltd., Tianjin, SD-M2000A)

(4) Portable digital color ultrasound diagnostic instrument (LAB version) (VINNO Technology Co., Ltd., VINNO 6 LAB)

(5) Fully automatic dehydrator (Leica, ASP200S)

(6) Paraffin microtome (Leica, RM2235)

(7) Slide baking table (Leica, HI1220)

(8) Water bath (Leica, HI1220)

(9) Heating paraffin embedding system (Leica, G1150 H)

(10) Microscope (Leica, DM3000)

(11) Upright fluorescence microscope (Leica, DM3000)

**1.2.2 Main Reagents and Consumables**

(1) Xylene (Sinopharm)

(2) Anhydrous ethanol (Sinopharm)

(3) Eosin (MDL, MD911467)

(4) Hematoxylin staining solution (MDL, MD911477)

(5) Neutral balsam (MDL, MD911683)

(6) VG staining solution (LEAGENE, DC0047)

(7) Citrate buffer (pH 6.0) (Zhongshan Golden Bridge, ZLI-9064)

(8) PBS buffer (pH 7.2-7.4) (Zhongshan Golden Bridge, ZLI-9061)

(9) Masson staining kit (MDL, MD7056)

**1.3 Experimental Methods**

**1.3.1 Experimental Grouping**

(1) Healthy control group (Control Group) (n=6): Six rats were selected from the initial 8 healthy rats, which were intraperitoneally injected with an equal volume of normal saline and gavaged with an equal volume of normal saline, and fed with a normal diet. During the entire experimental period (8 weeks), the general status of the animals was closely observed, including mental state, activity ability, and hair color, and body weight changes were recorded once a week. The normal diet met the nutritional requirements of Wistar rats, containing appropriate amounts of carbohydrates (approximately 60%-70%), protein (approximately 18%-22%), fat (approximately 5%-10%), as well as abundant vitamins and minerals to ensure that the animals maintained a healthy growth state. If any rat died accidentally or had poor health status affecting the experimental results, a suitable individual would be selected from the remaining 2 backup rats in this group for supplementation, ensuring that the final sample size for analysis was 6.

(2) Healthy gavage bacteria group (*Segatella* group) (n=6): Similarly, 6 rats were selected from the initial 8 rats, which were intraperitoneally injected with an equal volume of normal saline and gavaged with an equal volume of bacterial solution (gavaged twice a week, dose of 1 mL/100 g), and fed with a normal diet (same as the control group). In addition to the established gavage and feeding protocols, the daily status of the animals was closely monitored and body weight changes were recorded. The source, concentration, and activity of the bacterial solution used were recorded in detail to ensure the reproducibility and accuracy of the experiment. If any rat in this group had problems, a qualified individual from the backup rats would be selected for supplementation to maintain a sample size of 6.

(3) Healthy rats + gavage bacteria + LCD group (*Segatella* + LCD group) (n=6): Six rats were selected from 8 rats, which were intraperitoneally injected with an equal volume of normal saline, gavaged with an equal volume of bacterial solution, and fed with an LCD diet (gavaged twice a week, dose of 1 mL/100 g). During the experiment, in addition to observing the general status and body weight changes of the animals, attention was paid to their adaptability to LCD, such as whether there were adverse reactions such as loss of appetite and diarrhea, which were recorded and handled in a timely manner. If any rat could not continue the experiment for various reasons, backup rats were used for supplementation to ensure a stable sample size of 6.

(4) Chronic heart failure group (CHF group) (n=6): The CHF model was constructed by intraperitoneal injection of Dox (3.0 mg/kg, once a week for 8 weeks). Six rats were selected from the initial 8 rats, which were gavaged with an equal volume of normal saline and fed with a normal diet. During the modeling process, cardiac function indicators (regularly detected by echocardiography, such as LVEF and LVFS), body weight changes, and general status of the animals were closely monitored to ensure the success and stability of model construction. If any rat died or had health status that did not meet the experimental requirements during modeling, a suitable rat from the backup rats would be selected for supplementation to ensure that 6 rats were finally available for subsequent analysis. Throughout the experimental period, various physiological indicators and behavioral changes of the rats were closely monitored, and experimental data were recorded in detail.

(5) CHF + gavage bacteria group (CHF + *Segatella* group) (n=6): The CHF model was constructed (Dox injection method same as the CHF group). Six rats were selected from 8 rats, which were gavaged with an equal volume of bacterial solution (gavaged twice a week, dose of 1 mL/100 g) and fed with a normal diet. During the experiment, the cardiac function, body weight, general status, and possible adverse reactions related to the bacterial solution of the animals were continuously monitored, and various data were recorded in detail. If any rat died or could not continue the experiment due to health problems, a matched individual from the backup rats would be immediately selected for supplementation to ensure a sample size of 6.

(6) CHF + gavage bacteria + low-carbohydrate diet group (CHF + *Segatella* + LCD group) (n=6): The CHF model was constructed (Dox injection same as above). Six rats were selected from 8 rats, which were gavaged with an equal volume of bacterial solution and fed with an LCD diet (gavaged twice a week, dose of 1 mL/100 g). Throughout the experiment, the cardiac function, body weight, food intake, general status, and possible adverse reactions of the animals were comprehensively monitored to provide sufficient data support for subsequent result analysis. During the experiment, if any rat did not meet the experimental requirements, it was timely supplemented from the backup rats to ensure that 6 rats finally completed all experimental procedures and were used for data analysis.

**1.3.2 Experimental Modeling**

Doxorubicin (Dox) was administered via intraperitoneal injection at a dose of 3.0 mg/kg, once weekly for 8 consecutive weeks, with an injection volume of 10 mL/kg per administration. The control group received an equal volume of normal saline via the same route. Before and after modeling, the vital signs and recovery status of rats were closely monitored, including the following specific indicators:

(1) Daily observation of rats' activity ability, recording the frequency of spontaneous activities, activity range, and motor coordination. If rats exhibited symptoms such as slow movement or limb weakness, detailed records were made and the causes were analyzed.

(2) Monitoring of heart rate and respiratory rate: Professional physiological monitoring equipment was used to measure the heart rate and respiratory rate of rats at fixed time points (9:00-10:00 AM daily). If there was a significant fluctuation in heart rate (e.g., an increase or decrease of more than 20% compared with the baseline heart rate) or abnormal acceleration or slowing of respiratory rate, further evaluation of the rats' health status was required.

(3) Recording of daily food intake and water consumption: If food intake decreased by more than 30% or water consumption changed abnormally (increase or decrease by more than 50%), the causes were promptly identified and corresponding measures were taken.

**1.3.3 Bacterial Activation and Culture**

The methods for bacterial activation and culture were consistent with those described in Part 2. Briefly, Columbia blood agar plates were first deoxygenated. Bacterial lawns were scraped to prepare bacterial suspensions, which were then spread on plates for cultivation to complete activation. For subculture, single colonies were picked and inoculated into liquid medium for shaking culture. The concentration of the bacterial solution was determined, and a final concentration of 3.75×10⁸ CFU/mL was obtained for subsequent experiments.

**1.3.4 Bacterial Centrifugation and Rat Gavage**

Bacterial solution with a concentration of 3.75×10⁸ CFU/mL was transferred to an appropriate centrifuge tube and centrifuged in a high-speed refrigerated centrifuge (set at 4°C to maintain bacterial activity) at 3000 r/min for 10 min. After centrifugation, the supernatant was carefully discarded, and the pellet was resuspended in 1 mL of PBS. During resuspension, the centrifuge tube was placed on a vortex oscillator and vortexed at medium intensity for 30 s-1 min to ensure thorough mixing of bacteria.

The prepared bacterial solution for rat gavage was administered at a dose of 1 mL/100 g. A 20-22G gavage needle was used for gavage, and the needle was lubricated (with a small amount of sterile paraffin oil) before operation. Rats were fixed in a holder with their heads slightly tilted upward. The gavage needle was slowly inserted into the esophagus through the oral cavity to a depth of approximately 3-5 cm, followed by slow injection of the bacterial solution to avoid reflux or damage to the rat digestive tract.

**1.3.5 Safety Evaluation**

**1.3.5.1 Weekly Detection of Rat Body Weight, Heart Rate, and Blood Pressure**

(1) Rat body weight measurement: In the experiment, rats were individually identified and marked with ear tags to ensure clear identification of each rat. After random grouping, the body weight of rats was measured weekly to monitor their health status and experimental progress. During weighing, the "Operating Procedures for Handling and Restraining Laboratory Animals" were followed. First, the electronic balance (with a precision of 0.01 g) was tared. Then, rats were gently picked up from the breeding box and quickly placed on the balance. The weight was recorded after the reading stabilized. This process was performed in a quiet environment to avoid rat stress, ensuring data accuracy and animal welfare.

(2) Rat heart rate monitoring: Rats were anesthetized with isoflurane, fixed in a supine position on a rat platform, and connected to a biological signal acquisition and processing system with a sampling frequency of 1500 Hz and a gain of 20 dB. Recording started 2 min after anesthesia when the rats were stable, with each recording lasting at least 30 s. Measurements were repeated 3 times, and the average value was calculated to improve data reliability.

(3) Rat blood pressure monitoring: Rats were anesthetized with isoflurane, fixed in a supine position on a rat platform, and connected to the same biological signal acquisition and processing system. The cuff method was used, with a cuff pressure rise rate of 30 mmHg/s, a sampling frequency of 800 Hz, and a gain of 10 dB. Recording started 2 min after anesthesia when the rats were stable, and measurements were also repeated 3 times to calculate the average value.

**1.3.5.2 Ultrasonographic Examination**

Eight weeks after modeling, rats were anesthetized with isoflurane, and the fur on the left chest was removed using an electric shaver. Rats were fixed in a supine position, and an appropriate amount of coupling agent was applied. A color ultrasound diagnostic instrument equipped with a 10 MHz ultrasound probe was used, with parameters adjusted as follows: gain of 40 dB, depth of 4 cm, and frame rate of 30 frames/second, ensuring clear and highly reproducible images. The following cardiac indicators were observed: left ventricular ejection fraction (LVEF), left ventricular fractional shortening (LVFS), heart rate (HR), left ventricular internal diameter at end-diastole (LVIDd), left ventricular internal diameter at end-systole (LVIDs), left ventricular anterior wall thickness at end-diastole (LVAWd), left ventricular anterior wall thickness at end-systole (LVAWs), left ventricular posterior wall thickness at end-diastole (LVPWd), and left ventricular posterior wall thickness at end-systole (LVPWs). Each indicator was measured 3 times, and the average value was used as the final data.

**1.3.5.3 Serum Detection of BNP and cTnI**

In the rat experiment, to detect serum levels of brain natriuretic peptide (BNP) and cardiac troponin I (cTnI), blood samples were collected from rats at 2, 4, and 8 weeks after modeling. Before collection, rats were properly handled in accordance with the *Operating Procedures for Handling and Restraining Laboratory Animals* to reduce their stress response. Blood was collected via the orbital venous plexus under isoflurane anesthesia to ensure animal welfare. The collected blood samples were immediately placed in test tubes containing 10 µL/mL heparin sodium, and centrifuged at 1000-3000 rpm for 10-15 min at 4°C to separate serum. Serum samples were stored at -20°C until detection. For detection, ELISA kits were used to quantitatively analyze BNP and cTnI according to the manufacturer’s instructions. All test results were recorded in detail.

**1.3.6 Weighing of Isolated Tissues and Measurement of Tibial Length**

At the end of the experiment, rats were first weighed using an electronic balance with a precision of 0.001 g, and the data were recorded. Subsequently, the heart, lungs, and tibias of the rats were carefully dissected. During dissection, excessive pulling and damage to surrounding tissues were avoided to maintain tissue integrity. After dissection, the heart and lungs were quickly placed in a Petri dish containing an appropriate amount of normal saline, and the dish was gently shaken to rinse off blood and contaminants on the tissue surface. The surface moisture of the tissues was then gently blotted with clean filter paper to ensure the tissues were relatively dry without excessive squeezing. The processed heart and lungs were weighed separately using an electronic balance with a precision of 0.001 g, with each tissue weighed 3 times, and the average value was taken as the final wet weight data, which were recorded in detail. After dissection, the tibias were measured using an electronic caliper with a precision of 0.01 mm. During measurement, the tibia was placed horizontally on a platform, with the measurement starting from the proximal articular surface and ending at the distal medial malleolus tip, ensuring the caliper was perpendicular to the long axis of the tibia. The tibial length of each rat was measured 3 times, and the average value was taken as the final tibial length data, which were recorded in detail.

**1.3.7 Methods for Detecting Pathological Morphology and Protein Expression in Cardiac Tissues**

After the rats were euthanized, cardiac tissues were collected for pathological analysis. Hematoxylin-eosin (HE) staining, Masson staining, and immunohistochemistry were used to evaluate cardiomyocyte morphology and structure, myocardial fibrosis degree, and specific protein expression levels, respectively.

**1.3.7.1 HE Staining**

HE staining is a widely used technique in pathological research, which can clearly display the cell morphology, nuclear structure, and intercellular relationships of myocardial tissues. It provides morphological evidence for judging abnormalities such as cardiomyocyte lesions, necrosis, or hypertrophy, and is of great significance for studying the pathological mechanisms of cardiac diseases such as CHF. The specific experimental steps are as follows:

(1) Section preparation: 4-5 µm thick tissue sections were cut from paraffin blocks;

(2) Deparaffinization: Sections were sequentially placed in xylene for gradual deparaffinization;

(3) Hydration: Sections were hydrated through 100%, 95%, and 70% ethanol solutions in sequence, and finally transferred to water;

(4) Hematoxylin staining: Sections were immersed in hematoxylin staining solution, with the staining time determined according to the laboratory standard procedures;

(5) Differentiation: 1% hydrochloric acid-ethanol differentiation solution was used to remove excess hematoxylin, retaining nuclear staining;

(6) Bluing: Sections were briefly immersed in an alkaline solution (e.g., hematoxylin bluing solution) to enhance staining contrast;

(7) Dehydration: Sections were dehydrated through 70%, 95%, and 100% ethanol solutions in sequence;

(8) Clearing: Xylene was used to remove residual ethanol;

(9) Mounting: Canada balsam mounting medium was added dropwise, and a coverslip was placed and gently pressed to remove air bubbles;

(10) Drying: Mounted sections were dried at room temperature or using a mounting machine to accelerate drying;

(11) Observation: Stained sections were observed, and tissue structure and cell morphology characteristics were recorded.

**1.3.7.2 Masson Staining**

Masson staining is a technique used to display the distribution of collagen fibers in tissues, which can accurately evaluate the degree and spatial distribution of myocardial fibrosis and is of great significance for studying myocardial structural remodeling. The experimental steps are as follows:

(1) Tissue embedding

① Ethanol dehydration: Tissues were sequentially immersed in 70%, 80%, 90%, 95%, 100%, and 100% ethanol solutions, with 40 min of dehydration at each grade;

② Clearing: Tissues were sequentially placed in three xylene cylinders, with 1 h of immersion in each cylinder.

③ Wax infiltration: Tissues were sequentially immersed in three paraffin cylinders, with 1 h of infiltration in each cylinder.

④ Embedding: Liquid paraffin was poured into a mold, and the tissue block was placed flat at the bottom with the cut surface facing down. After the paraffin solidified, the embedding frame was removed. After complete cooling, the wax block was trimmed, retaining an appropriate amount of paraffin for sectioning.

(2) Section preparation and deparaffinization

① Sectioning: The pre-cooled wax block was fixed on a microtome, the cut surface was adjusted to be parallel to the knife edge, the knife angle was set to 15°, the section thickness was adjusted to 4 μm, and uniform sections were cut;

② Section spreading: Sections were gently lifted with a brush and placed face down in a section spreading box at approximately 45°C.

③ Section fishing: After the sections were flattened, a glass slide was vertically inserted into the water to attach the sections, and tweezers were used to assist in pushing the sections to the two-thirds position of the slide.

④ Slide baking: The slides with attached sections were air-dried, then baked in a 65°C slide baking machine for 1 h, and then transferred to an oven for baking for 2 h.

⑤ Deparaffinization: Sections were sequentially immersed in xylene I, xylene II, and xylene III for 10 min each, anhydrous ethanol I and anhydrous ethanol II for 5 min each, 90% ethanol, and 80% ethanol for 5 min each.

(3) Staining:

① Nuclei were stained with Weigert’s iron hematoxylin solution;

② Staining with Biebrich scarlet-acid fuchsin solution;

③ Treatment with phosphomolybdic-phosphotungstic acid solution;

④ Counterstaining of collagen fibers with aniline blue solution;

⑤ After dehydration, clearing, and mounting, the distribution of collagen fibers and the degree of myocardial fibrosis were observed under a microscope;

**1.3.7.3 Immunohistochemistry**

Immunohistochemistry is a technique based on the specific binding of antibodies to antigens. It enables the localization, qualitative, and relative quantitative analysis of antigens (such as polypeptides and proteins) in tissue cells by the chromogen labeled on antibodies through chemical reactions. In this experiment, this method was used to detect the expression of P53, Cleaved-caspase-3, TLR4, and NF-κB p65 proteins in cardiac tissues. The specific steps are as follows:

(1) Section preparation: Sections with a thickness of 3-5 µm were cut from paraffin blocks of cardiac tissues, floated in warm water at 40-45°C to flatten, fished out with glass slides, and then baked in a 60°C oven for 30-60 min to ensure firm adhesion of the sections.

(2) Deparaffinization and hydration: The sections were sequentially immersed in xylene I and xylene II for 10-15 min each for deparaffinization, then hydrated through 100% ethanol I, 100% ethanol II, 95% ethanol, and 70% ethanol, with each step lasting 3-5 min. Finally, they were washed with distilled water 2-3 times, 3-5 min each time.

(3) Antigen retrieval: The sections were placed in citrate antigen retrieval solution (pH 6.0), heated to boiling with high fire in a microwave oven, then switched to medium fire to maintain slight boiling for 10-15 min, and naturally cooled to room temperature. After cooling, they were washed with PBS buffer (pH 7.2-7.4) 3 times, 3-5 min each time.

(4) Blocking of endogenous peroxidase: The sections were immersed in 3% hydrogen peroxide solution and incubated at room temperature for 10-15 min to block endogenous peroxidase activity. After incubation, they were washed with PBS buffer 3 times, 3-5 min each time.

(5) Blocking: Normal goat serum blocking solution was added dropwise, and incubated at room temperature for 30-60 min to reduce non-specific binding. After blocking, the blocking solution was not washed away, and the next step was performed directly.

(6) Primary antibody incubation: P53 (1:200), Cleaved-caspase-3 (1:100), TLR4 (1:300), and NF-κB p65 (1:500) antibodies were diluted according to the instructions. The diluted antibodies were added dropwise to the sections and incubated at 4°C overnight. The next day, the sections were washed with PBS buffer 3 times, 5-10 min each time.

(7) Secondary antibody incubation: Diluted secondary antibody was added dropwise, incubated at room temperature for 30-60 min, and after incubation, the sections were washed with PBS buffer 3 times, 5-10 min each time.

(8) Color development (only applicable to enzyme-labeled secondary antibodies): The chromogenic working solution was prepared according to the instructions of the DAB chromogenic kit, added dropwise to the sections, incubated at room temperature for 3-10 min, and the color development was observed under a microscope. After clear color development, the reaction was terminated immediately by rinsing with distilled water.

(9) Hematoxylin counterstaining: The sections were immersed in hematoxylin staining solution for 3-5 min, rinsed with tap water for 10-15 min for bluing, then differentiated with 1% hydrochloric acid ethanol for a few seconds, and rinsed with tap water to enhance contrast.

(10) Dehydration, clearing, and mounting: The sections were dehydrated through 70% ethanol, 95% ethanol, 100% ethanol I, and 100% ethanol II in sequence, 3-5 min each step; then immersed in xylene I and xylene II for clearing, 5-10 min each step; finally, neutral balsam was added dropwise for mounting, and after drying, observed under a microscope.

(11) Result analysis: The localization (nucleus, cytoplasm, or cell membrane) and staining intensity of proteins were observed using an optical microscope. ImageJ software was used for quantitative analysis of the average optical density or integrated optical density of positive regions to compare protein expression differences among different experimental groups.

**1.3.7.4 WB Detection**

To further verify the expression levels of key proteins (TLR4, MyD88, NF-κB p65) in cardiac tissues, WB technology was used for quantitative analysis of cardiac tissue samples in this study. The specific methods are as follows:

(1) Extraction of cardiac tissue proteins

① Total protein extraction (for detection of TLR4 and MyD88): Approximately 50 mg of cardiac tissue was taken, added with RIPA lysis buffer containing 1% PMSF and phosphatase inhibitors, and homogenized on ice. Lysed on ice for 30 min, with vortex oscillation every 10 min during this period. Centrifuged at 12,000 rpm for 15 min at 4°C, and the supernatant was collected as total protein and stored at -80°C.

② Nuclear protein extraction (for detection of nuclear NF-κB p65): Cell collection was performed as per the total protein extraction steps. Cytoplasmic/nuclear separation was carried out according to the kit instructions: cytoplasmic lysis buffer was added, lysed on ice for 15 min, and centrifuged at 500 g for 5 min. The supernatant was cytoplasmic protein, and the pellet was lysed with nuclear lysis buffer on ice for 30 min, then centrifuged at 12,000 rpm for 10 min, and the supernatant was collected as nuclear protein.

(2) Protein concentration determination (BCA method)

① BSA standard solutions (0-1.6 mg/mL) were diluted.

② Sample dilution: Total protein was diluted 10-fold, and nuclear protein was diluted 5-fold.

③ 20 µL of sample/standard + 200 µL of BCA working solution was added to each well, and incubated at 37°C for 30 min.

④ The absorbance at 562 nm was measured with a microplate reader, and the protein concentration was calculated.

(3) SDS-PAGE electrophoresis

① Separation gel preparation: Separation gels with different concentrations were prepared according to the molecular weight of the target protein (see Table 3-2), overlaid with deionized water, and allowed to stand at room temperature for 40 min until solidified.

② Stacking gel preparation: Deionized water was poured off, and residual liquid was aspirated. The gel plate was placed vertically, 4 mL of 5% stacking gel was added (avoiding bubbles), a sample comb was inserted, and allowed to stand at room temperature for 40 min until solidified.

③ Electrophoresis tank installation: The sample comb was removed, and the glass (with the "concave" side inward) was fixed in the electrophoresis tank.

④ Electrophoresis conditions: Constant voltage of 80 V for the stacking gel, approximately 20 min; constant voltage of 120 V for the separation gel, and electrophoresis was performed until the bromophenol blue indicator reached the bottom of the gel.

**Table 3-2 SDS-PAGE gel preparation**

| Target Protein | Concentration of Separating Gel | Loading Amount |
| --- | --- | --- |
| TLR4（95kDa） | 10% | 30μg |
| MyD88（33kDa） | 12% | 30μg |
| NF-κB p65（65kDa） | 12% | 15μg |

(4) Wet Transfer for Membrane Blotting

① Transfer conditions for high-molecular-weight proteins (TLR4): Constant voltage of 100 V for 1.5 hours at 4°C.

② Transfer conditions for low-molecular-weight proteins (MyD88): Constant voltage of 100 V for 1 hour at 4°C.

③ Nuclear NF-κB p65: Constant voltage of 100 V for 1 hour; PVDF membrane activated with methanol.

④ Transfer buffer: 25 mM Tris-base, 192 mM glycine, 20% methanol (pH 8.3).

(5) Antibody Incubation and Visualization

① Blocking: 5% skim milk in TBST, incubated at room temperature for 1 hour.

② Primary antibody incubation: Primary antibodies were diluted according to the manufacturer’s instructions (details shown in Table 3-3) and incubated at 4°C overnight.

③ Secondary antibody incubation: HRP-conjugated goat anti-rabbit/mouse IgG (1:5000), incubated at room temperature for 1 hour.

④ ECL visualization: ECL solutions A and B were mixed at a 1:1 ratio and evenly applied to the membrane surface. Signals were captured using a chemiluminescence imaging system (exposure time: 10 s – 5 min).

⑤ Internal reference normalization: For total proteins: Gray value of target protein / Gray value of β-actin; For nuclear proteins: Gray value of nuclear NF-κB p65 / Gray value of Lamin B1.

**Table 3-3 Table of Primary Antibody Incubation Conditions**

| Antibody | Dilution Ratio | Buffer | Incubation Condition |
| --- | --- | --- | --- |
| TLR4 | 1:1000 | 5% BSA/TBST | 4°C overnight |
| MyD88 | 1:800 | 5% Skim Milk/TBST | 4°C overnight |
| Nuclear NF-κB p65 | 1:800 | 5% Skim Milk/TBST | 4°C overnight |
| β-actin | 1:5000 | 5% Skim Milk/TBST | Room temperature for 2 h |

**1.3.8 16S rRNA Detection**

The detection of the 16S rRNA gene is a commonly used method in microbial diversity research. Through sequencing and analysis of the 16S rRNA gene, the composition and structure of the microbial community can be revealed. The experimental process mainly includes the following steps:

(1) DNA Extraction: First, genomic DNA is extracted from rat feces. During the extraction process, the Y-axis linkage extraction method is used or the extracted genomic DNA is detected to ensure the purity and integrity of the DNA.

(2) PCR Amplification: Specific primers with barcodes are used to perform PCR amplification on the variable regions of the 16S rRNA gene. To ensure the accuracy and reliability of subsequent data analysis, the following conditions need to be met:

① Amplification with as low a cycle number as possible;

② Ensure that the number of amplification cycles is consistent for each sample.

③ The PCR reaction uses TransGen AP221-02: TransStart Fastpfu DNA Polymerase. The PCR products are detected by 1% agarose gel electrophoresis to ensure that the size of the amplified products meets the expectations **^[31]^** .

(3) Library Construction: The PCR products are used for library construction. The specific steps include:

① Ligation of "Y"-shaped adapters;

② Use of magnetic beads for screening to remove adapter self-ligated fragments;

③ Enrichment of the library by PCR amplification;

④ Perform normalization treatment to generate single-stranded DNA fragments **^[32]^**.

(4) Sequencing on the Machine: The constructed library is sequenced on a second-generation high-throughput sequencing platform. The process includes:

① One end of the DNA fragment is complementary to the primer end group and fixed on the chip;

② The other end is complementary to another primer to form a "bridge";

③ DNA clusters are generated by PCR amplification;

④ The DNA amplicon strands are converted into single strands;

⑤ Add modified DNA polymerase and all 4 types of fluorescently labeled dNTPs;

⑥ Use a laser to scan the reaction plate and read the type of nucleotide polymerized in the first round of reaction for each horizontal marker sequence;

⑦ Chemically cleave the "fluorescent gene" and "terminator gene" to restore the 3' end activity and continue to polymerize the second nucleotide;

⑧ Count the fluorescent signal results collected in each round to obtain the sequence of the DNA fragment **^[33]^**.

(5) Data Analysis

① Data Quality Control: The raw data obtained from sequencing is in Fastq format, including the name of each sequencing sequence, the base sequence, and the corresponding sequencing quality information. Trimmomatic and Pearl are used to perform quality control on the Fastq data, removing low-quality sequences and sequences containing N. The specific parameters are as follows:

② Trimmomatic uses a sliding window screening method, with the window size set to 50 bp, the average quality value set to 20, and the minimum retained sequence length set to 120 bp;

③ Pearl is used to remove sequences containing N **^[34]^**.

④ Sequence Assembly: Flash and Pearl are used to perform assembly processing on the two-end sequences according to the overlap relationship of PE (Pair-End) sequences. The minimum overlap is set to 10 bp, and the mismatch rate is 0.1, to obtain Fasta sequences **^[35]^**.

⑤ OTU Clustering: UCLUST and UPARSE are used to perform OTU (Operational Taxonomic Units) clustering on the sequences, usually with a 97% similarity for OTU division. After OTU clustering, algorithms such as RDP Classifier are used to perform species annotation on the OTU representative sequences to obtain species information at each taxonomic level **^[36]^**.

⑥ Diversity Analysis: Based on the OTU clustering results, Alpha diversity and Beta diversity analyses are performed. Alpha diversity analysis includes rarefaction curves, Rank- abundance curves, species accumulation curves, etc.; Beta diversity analysis reveals the differences in community structure among samples through methods such as PCA, NMDS, and PLS-DA **^[37]^**.

**1.3.9 qPCR Detection**

qPCR technology was used to detect the gene expression levels of inflammatory factors (TNF-α, IL-6, etc.) as well as TLR4, MyD88, and NF-κB p65 in cardiac tissues, through quantitative analysis of the expression changes of these genes.

**1.3.9.1 Primer Sequence Information**

Primers were designed based on the target gene sequences, following the principles below: appropriate primer length (18–25 bp); GC content controlled between 40%–60%; avoidance of secondary structure and primer dimer formation to ensure specificity and amplification efficiency. Professional primer design software (e.g., Primer Premier) was used for design, and primer specificity was verified by tools such as BLAST. Detailed primer sequence information is shown in Table 3-4.

**Table 3-4 Primers used in this work**

| Target Name | Primer | Primer Sequence（5'→3'） | Product length | Annealing temperature |
| --- | --- | --- | --- | --- |
| IL-6 | F | CTGGTCTTCTGGAGTTCCGT | 120 bp | 58°C |
|  | R | AGAGCATTGGAAGTTGGGGT |  |  |
| TNF-α | F | TCTTCAAGGGACAAGGCTGC | 150 bp | 58°C |
|  | R | CTTGATGGCAGAGAGGAGGC |  |  |
| TLR4 | F | AGACCTGTCCCTGAACCTATGG | 152 bp | 58°C |
|  | R | CAGAGCATCACCTCAGGTCTTC |  |  |
| MyD88 | F | ATGGCGAACTACATCGCCAA | 198 bp | 58°C |
|  | R | TCAGGAAGCCGAGTTGTAGC |  |  |
| NF-κB p65 | F | GAGACCTTGGCTGTTGCCAT | 185 bp | 60°C |
|  | R | CGGGAATGTCAGCTGCTTCT |  |  |

**1.3.9.2 qPCR Experimental Procedure**

(1) Extraction of Total RNA from Samples (Trizol Method)

① Based on the properties of Trizol reagent, its main component guanidinium isothiocyanate can lyse cells, separate RNA from proteins, and simultaneously inhibit RNase activity to prevent RNA degradation. An appropriate amount of samples preserved in liquid nitrogen or frozen was taken and rapidly ground into powder to ensure sufficient cell disruption. 1 mL of Trizol was added, and the mixture was transferred to a 1.5 mL EP tube and mixed well.

② 200 µL of chloroform was added to the EP tube, followed by vigorous oscillation for 15-30 seconds to form an emulsion. After standing for 5 minutes, the solution, with RNA mainly present in the upper aqueous phase. The mixture was centrifuged at 12,000 rpm for 10 minutes at 4°C, and the supernatant was carefully transferred to a new EP tube, avoiding aspiration of the protein layer and organic phase.

③ Isopropanol was added to the supernatant at a 1:1 ratio, and the mixture was gently mixed to promote RNA precipitation. After centrifugation at 12,000 rpm for 10 minutes at 4°C, the supernatant was discarded, and the RNA precipitate was attached to the bottom of the tube. 750 µL of anhydrous ethanol was added to suspend the RNA precipitate, followed by centrifugation at 12,000 rpm for 5 minutes at 4°C, and the supernatant was discarded. The RNA precipitate was air-dried at room temperature, avoiding excessive drying. Finally, the RNA precipitate was dissolved in 50 µL of DEPC-treated water, and gently pipetted to ensure complete dissolution.

④ A nucleic acid concentration analyzer was used to detect RNA concentration and purity. The A260/A280 ratio should be between 1.8 and 2.1, indicating minimal protein contamination. 1-2 µL of RNA was subjected to 1% agarose gel electrophoresis to observe the 28S and 18S rRNA bands. Intact RNA should exhibit clear 28S and 18S bands, with the brightness of the 28S band approximately twice that of the 18S band, and no obvious tailing or smearing. Only RNA of qualified quality was used for subsequent experiments.

(2) Reverse Transcription for cDNA Synthesis

① Using the ExonScript RT SuperMix with dsDNase reverse transcription kit (EXONGEN), cDNA was synthesized with RNA as the template under the action of reverse transcriptase, providing a template for subsequent PCR amplification. The specific reaction system is shown in Table 3-5:

**Table 3-5 Reverse transcription construction system**

| Component | Volume |
| --- | --- |
| RNA | 1-2µg |
| 5× Reaction | 4µL |
| Supreme Enzyme Mix | 3µL |
| DEPC H2O | Up to 20 μL |

② The reaction system was gently mixed, followed by brief centrifugation to collect the liquid at the bottom of the tube, ensuring uniform mixing of all components without bubble formation. The reaction was performed on a PCR instrument with the program set as shown in Table 3-6.

**Table 3-6 Reverse transcription program**

| Temperature | Time |
| --- | --- |
| 25℃ | 10min |
| 55℃ | 15min |
| 85℃ | 5min |

③ After the reaction, the cDNA products were stored at -20°C for later use. During the reverse transcription process, negative controls (no - template controls, i.e., without adding RNA templates) and positive controls (such as standard samples with known concentrations or verified positive samples) were set to evaluate the reaction efficiency and detect potential contamination.

(3) Real - Time Fluorescent Quantitative Detection

① Real - time fluorescent quantitative PCR monitors the PCR amplification process in real - time based on the accumulation of fluorescent signals. In this experiment, the SYBR Green method was adopted. SYBR Green can bind to double - stranded DNA to generate fluorescent signals. As the PCR amplification proceeds, the intensity of the fluorescent signal continuously increases. The expression level of the target gene is quantitatively analyzed by detecting the changes in the fluorescent signal. The reaction system was established as shown in Table 3 - 7.

**Table 3-7 Real time PCR reaction system**

| Component | Volume |
| --- | --- |
| cDNA | 1µL |
| 2×qPCR mix | 10µL |
| Primer F | 0.4µL |
| Primer R | 0.4µL |
| ddH2O | Up to 20µL |

② After mixing the reaction system thoroughly, a brief centrifugation was performed, and the mixture was placed in a real-time fluorescent quantitative PCR instrument. The reaction was carried out under the conditions specified in Table 3-8.

**Table 3-8 qPCR reaction conditions**

| Step | Temperature | Time | Cycle Number |
| --- | --- | --- | --- |
| Initial Denaturation | 95°C | 5min | 1 |
| Denaturation | 95°C | 10sec | 40 |
| Annealing | 58°C | 20sec | 40 |
| Extension | 72°C | 20sec | 40 |
| Melting Curve Analysis | 95°C | 15sec | 1 |
|  | 60°C | 60°C | 1 |
|  | 95°C | 15sec | 1 |

③ During the qPCR process, negative controls (no - template control and no - primer control) and positive controls were set to ensure the accuracy of the results. After the experiment was completed, the relative gene expression levels were calculated using the 2^(-ΔΔCt) method based on the Ct values, and statistical analyses were performed using software such as SPSS 22.0 or GraphPad Prism 10.0. One - way analysis of variance or other appropriate statistical methods were used to compare differences between groups, with P < 0.05 set as the significance threshold.

**1.3.10 ELISA Detection**

ELISA technology was used to detect inflammatory factors (TNF-α, IL-6, etc.) in cardiac tissues and LPS content in serum, aiming to explore the role and changes of inflammatory responses and gut microbiota-related metabolites in cardiac diseases. The specific steps are as follows:

1.3.10.1 Detection of Inflammatory Factors in Myocardial Tissues and LPS in Serum

(1) Steps for detecting inflammatory factors in myocardial tissues

① Strip preparation: Take out the required strips from the aluminum foil bag equilibrated at room temperature for 20 min. The remaining strips are sealed and stored at 4°C. Avoid touching the surface of the strips during operation to prevent contamination.

② Sample addition: Set standard wells and sample wells. Add 50 µL of standard solutions with different concentrations (gradient set according to the kit instructions) to the standard wells; add 10 µL of the myocardial tissue sample to be tested and 40 µL of diluent to the sample wells, and mix well to avoid bubbles. No substances are added to the blank wells.

③ Incubation: Except for the blank wells, add 100 µL of HRP-labeled detection antibody to each well. After sealing the plate, incubate at 37°C for 60 min, with gentle shaking every 15 min during the period.

④ Plate washing: Discard the liquid, invert the plate and pat dry. Fill each well with washing solution, let stand for 1 min, then discard the washing solution, and repeat plate washing 5 times.

⑤ Color development: Add 50 µL of substrate A and 50 µL of substrate B to each well, and incubate at 37°C in the dark for 15 min, avoiding bubbles.

⑥ Termination of reaction and detection: Add 50 µL of stop solution to each well, shake gently to mix, and measure the OD value at 450 nm with a microplate reader within 15 min.

(2) Steps for detecting serum LPS

① Sample preparation: Take out the collected serum samples from the refrigerator, thaw at room temperature and mix gently to avoid vigorous shaking which may produce bubbles. If there is precipitation in the sample, centrifuge at low speed (3000-4000 rpm for 5-10 min) and take the supernatant for detection.

② Strip preparation: Take out the required strips from the aluminum foil bag equilibrated at room temperature for 20 min. The remaining strips are sealed and stored at 4°C. Avoid touching the surface of the strips during operation.

③ Sample addition: Set standard wells, sample wells and blank wells. Add 50 µL of standard solutions with different concentrations (gradient set according to the kit instructions) to the standard wells; add 10 µL of the serum sample to be tested and 40 µL of diluent to the sample wells, and mix gently, taking care to avoid producing bubbles. No substances are added to the blank wells.

④ Incubation: Except for the blank wells, add 100 µL of the corresponding HRP-labeled detection antibody (antibody for LPS detection) to each well. After sealing the plate, place the microplate in a 37°C constant temperature incubator and incubate for 60 min, with gentle shaking every 15 min during incubation to ensure sufficient reaction.

⑤ Plate washing: After incubation, discard the liquid in the wells, invert the microplate on absorbent paper and pat dry. Then fill each well with washing solution (the washing solution should be prepared according to the kit requirements), let stand for 1 min, then discard the washing solution, and repeat plate washing 5 times to completely remove unbound substances and reduce background interference.

⑥ Color development: Add 50 µL of substrate A and 50 µL of substrate B to each well, mix gently, and incubate the microplate in a 37°C dark environment for 15 min, avoiding bubble formation. During incubation, pay attention to observing the color change in the wells to avoid over-coloring or under-coloring.

⑦ Termination of reaction and detection: After incubation, add 50 µL of stop solution to each well, shake gently to mix, and measure the OD value of each well at 450 nm with a microplate reader within 15 min.

**1.3.10.2 Standard Curve Drawing**

(1) Inflammatory factors in myocardial tissues: Draw a standard curve with the standard concentration as the abscissa and the corresponding OD value as the ordinate. Calculate the concentration of inflammatory factors in myocardial tissue samples through the standard curve.

(2) Serum LPS: Similar to the detection of inflammatory factors in myocardial tissues, draw a standard curve with the standard concentration as the abscissa and the OD value as the ordinate, which is used to calculate the concentration of LPS in serum samples.

**1.3.10.3 Quality Control**

(1) Inflammatory factors in myocardial tissues: Set at least 3 replicate wells, calculate the CV value (coefficient of variation) between replicate wells, which is required to be less than 10% to evaluate the repeatability and accuracy of the experiment; set positive controls (samples known to contain target inflammatory factors) and negative controls (samples without target inflammatory factors). The positive control should show obvious positive reaction, and the OD value of the negative control should be lower than a certain threshold (less than 0.1) to monitor the effectiveness of the experiment.

(2) Serum LPS: Set at least 3 replicate wells for each serum sample, calculate the CV value between replicate wells to ensure that it is less than 10%; set positive controls (serum samples known to contain LPS) and negative controls (serum samples without LPS). The positive control should show obvious positive reaction, and the OD value of the negative control should be lower than the set threshold (less than 0.1) to monitor the effectiveness of the serum LPS detection experiment. If the positive control shows no obvious positive reaction or the OD value of the negative control is higher than the threshold, a comprehensive inspection of the experimental process, including reagent quality and operation steps, is required to ensure the reliability of the experimental results.

**1.3.11 TMAO Determination (LC-MS/MS Method)**

(1) Sample pretreatment: For rat serum samples, after blood collection in the 8th week, centrifuge at 3000-4000 rpm for 10-15 min at 4°C to separate serum. Take 100 μL of serum and place it in a 1.5 mL centrifuge tube, add 400 μL of acetonitrile solution containing internal standard (such as d9-TMAO, concentration 10 μmol/L), vortex and oscillate for 3 min to fully precipitate proteins. Subsequently, centrifuge at 12000-14000 rpm for 15 min at 4°C, transfer the supernatant to a new centrifuge tube, and dry it under a nitrogen blower at 40°C. The residue is re-dissolved with 100 μL of initial mobile phase (0.1% formic acid aqueous solution: 0.1% formic acid acetonitrile solution = 95:5, v/v), vortex and mix for 1 min, centrifuge at 12000 rpm for 5 min, and take the supernatant and transfer it to an injection vial for testing.

(2) Liquid chromatography conditions: A C18 reversed-phase chromatographic column (2.1 mm × 100 mm, 1.7 μm) is used. The column temperature is set to 40°C to maintain stable separation efficiency. Mobile phase A is 0.1% formic acid aqueous solution, and mobile phase B is 0.1% formic acid acetonitrile solution. The elution gradient is as follows: 0-2 min, 95% A; 2-10 min, linear change to 5% A; 10-15 min, maintain 5% A; 15-17 min, linear change back to 95% A; 17-20 min, maintain 95% A. The flow rate is 0.3 mL/min, and the injection volume is 5 μL.

(3) Mass spectrometry conditions: An electrospray ion source (ESI) is used with positive ion mode scanning. The capillary voltage is set to 3.5 kV, and the cone voltage is 45 V. The scanning range is m/z 100-500, and the scanning time is 0.25 s. The parent ion of TMAO is m/z 76.1, the daughter ion is m/z 58.1, and the collision energy is 25 eV. For every 5 samples analyzed, insert 1 mixed quality control sample (serum sample with known TMAO concentration) to monitor instrument stability, and perform quality calibration regularly to ensure the accuracy of detection results.

(4) Quantitative analysis: Prepare a series of TMAO standard solutions with different concentrations (0.1, 0.5, 1, 5, 10, 50, 100 μmol/L), analyze according to the above sample pretreatment and detection conditions, and draw a standard curve with the standard concentration as the abscissa and the ratio of TMAO to internal standard peak area as the ordinate. Calculate the concentration of TMAO in the sample according to the regression equation of the standard curve. Each sample is determined in parallel 3 times, and the coefficient of variation (CV) is calculated, which is required to be <10% to ensure the repeatability and reliability of the results.

**1.3.12 Data Analysis**

Statistical software (SPSS 22.0, GraphPad Prism 10.0, etc.) was used to analyze the ELISA detection results. According to the experimental design, corresponding statistical tests (t-test, analysis of variance, etc.) were performed to compare the differences in the expression levels of inflammatory factors between different groups, with P < 0.05 set as statistically significant. "ns" indicates no difference, "*" indicates significance, where * represents P < 0.05, ** represents P < 0.01, *** represents P < 0.001, and so on. Meanwhile, descriptive statistical analysis was performed on the data, such as calculating the mean and standard deviation, to comprehensively display the experimental results.

# ***References***

1. National Center for Cardiovascular Diseases, National Committee of Cardiovascular Experts, Heart Failure Professional Committee, Chinese Medical Doctor Association, Heart Failure Professional Committee, et al. National Heart Failure Guidelines 2023[J]. Chinese Journal of Heart Failure and Cardiomyopathy, 2023, 07(4):215-311. DOI:10.3760/cma.j. issn.101460-20231209-00052.
2. Adamo, M, Gardner, RS, McDonagh, TA, et al. 2021 ESC Guidelines for the diagnosis and treatment of acute and chronic heart failure. EUR HEART J. 2022; 43 (6): 440-441. doi: 10.1093/eurheartj/ehab853.
3. Heidenreich, PA, Bozkurt, B, Aguilar, D, et al. 2022 AHA/ACC/HFSA Guideline for the Management of Heart Failure: A Report of the American College of Cardiology/American Heart Association Joint Committee on Clinical Practice Guidelines. CIRCULATION. 2022; 145 (18): e895-e1032. doi: 10.1161/CIR. 0000000000001063.
4. Chinese Society of Cardiology, Chinese Collaborative Group on Myocarditis and Cardiomyopathy. Chinese Guidelines for the Diagnosis and Treatment of Dilated Cardiomyopathy[J]. Journal of Clinical Cardiology, 2018, 34(5):421-434. DOI:10.13201/j. Issn.1001-1439.2018.05.001.
5. Richardson, P, McKenna, W, Bristow, M, et al. Report of the 1995 World Health Organization/International Society and Federation of Cardiology Task Force on the Definition and Classification of cardiomyopathies.CIRCULATION.1996; 93(5): 841-2. doi:10.1161/01.cir.93.5.841
6. Knuuti J, Wijns W, ESC Scientific Document Group, et al. 2019 ESC Guidelines for the diagnosis and management of chronic coronary syndromes. Eur Heart J. 2020 Jan 14; 41(3):407-477. doi:10. 1093/eurheartj/ehz425. Erratum in:Eur Heart J. 2020 Nov 21; 41(44):4242. doi:10. 1093/eurheartj/ehz825. PMID:31504439.
7. Structural Heart Disease Professional Committee, Society of Cardiovascular Physicians, Chinese Medical Doctor Association. Expert Consensus on Clinical Pathway for Transcatheter Aortic Valve Replacement in China (2021 Version)[J]. Chinese Circulation Journal, 2022, 37(1):12-23. DOI:10.3969/j.issn.1000-3614.2022.01.003.
8. Cardiovascular Imaging Group, Chinese Society of Cardiology; Cardiovascular Imaging Group, Beijing Medical Association. Expert Consensus on Standardized Echocardiographic Examination for Adult Heart Valve Disease in China[J]. Chinese Circulation Journal, 2021, 36(2):109-125. DOI:10.3969/j.issn.1000-3614.2021.02.002.
9. Chamberlain, JJ, Peterson, L, et al. Diabetes Technology: Review of the 2019 American Diabetes Association Standards of Medical Care in Diabetes. ANN INTERN MED. 2019; 171(6):415-420. doi:10. 7326/M19-1638
10. 2018 ESC/ESH Guidelines for the management of arterial hypertension. Rev Esp Cardiol(Engl Ed). 2019; 72(2):160. doi:10. 1016/j. rec. 2018. 12. 004
11. Tang WH, Hazen SL,et al.Intestinal microbial metabolism of phosphatidylcholine and cardiovascular risk. N Engl J Med. 2013 Apr 25;368(17):1575-84. doi: 10.1056/NEJMoa 1109400. PMID: 23614584; PMCID: PMC3701945.
12. Luedde, M, Winkler, T, Heinsen, FA, et al. Heart failure is associated with depletion of core intestinal microbiota. ESC Heart Fail. 2017; 4 (3): 282-290. doi: 10.1002/ehf2.12155
13. White, I. Sample Size Calculations in Clinical Research.J ROY STAT SOC A STA. 2008; 171 (3): 756-756. doi: 10.1111/j.1467-985x.2008.00538_2.x
14. Spoletini, I, Lainscak, M. Epidemiology and Prognosis of Heart Failure ICFJ. 2017; 10 doi:10. 17987/icfj. v10i0. 420
15. Chinese Medical Association, Editorial Office of Chinese Medical Association, Chinese Society of General Practice, et al. Chinese Guidelines for Primary - care Diagnosis, Treatment and Management of Heart Failure (2024) [J]. Chinese Journal of General Practitioners, 2024, 23(6): 549 - 577. DOI:10.3760/cma.j.cn114798 - 20231227 - 00476.
16. Wang Hui, Ju Feng. Applications and Prospects of Metagenomics in Environmental Microbiome Research [J]. Microbiology China,2024,51(6):1814-1833.DOI:10.13344/j.mic -robiol.china.231088.
17. He W, Zhao S, Liu X, et al. ReSeqTools:an integrated toolkit for large large-scale next next-generation sequencing based resequencing analysis. Genet Mol Res. 2013; 12(4):627 6275-6283.
18. T. Magoc and S. Salzberg. 2011. FLASH:Fast length adjustment of short reads to improve genome assemblies. Bioinformatics 27(21):2957-63.
19. Wang Q, Garrity GM, Tiedje JM, Cole JR. 2007. Naive Bayesian classifier for rapid assignment of rRNA sequences into the new bacterial taxonomy. Appl Environ Microbiol. 73:5261-5267.
20. Douglas G M, Maffei V J, Zaneveld J, et al. PICRUSt2:An improved and extensible approach for metagenome infer ence. bioRxiv, 2019.
21. Schloss,PD,Westcott,SL,Ryabin,T,et al.Introducing mothur:open-source, platform -independent,community-supported software for describing and comparing microbial communities.APPL ENVIRON MICROB.2009; 75(23): 7537-41. doi:10. 1128/AEM. 01541-09
22. Caporaso J G, Kuczynski J, Stombaugh J, et al. QIIME allows analysis of high-throughput community sequencing data[J]. Nature methods, 2010, 7(5):335.
23. Lozupone C, Lladser ME, Knights D, Stombaugh J, Knight R. UniFrac:an effective distance metric for microbial community comparison. ISME J. 2011; 5(2):16 9 172
24. Chang F, He S, Dang C. Assisted Selection of Biomarkers by Linear Discriminant Analysis Effect Size(LEfSe)in Microbiome Data[J]. J Vis Exp. 2022; (183):10. 3791/61715.
25. Deng T, Li J, He B, et al. Gut microbiome alteration as a diagnostic tool and associated with inflammatory response marker in primary liver cancer[J]. Hepatol Int. 2022; 16(1):99-111.
26. Zheng YY, Xie X, et al. Gut Microbiome-Based Diagnostic Model to Predict Coronary Artery Disease. J Agric Food Chem. 2020 Mar 18; 68(11):3548-3557. doi:10. 1021/acs. jafc. 0c00225. Epub 2020 Mar 6. PMID:32100534.
27. Dunn, W. B.; Broadhurst, D, et al. Procedures for large-scale metabolic profiling of serum and plasma using gas chromatography and liquid chromatography coupled to mass spectrometry. Nat Protoc 2011, 6(7), 1060-1083.
28. Barker, M.; Rayens, W., Partial least squares for discrimination. J Chemometr 2003, 17(3), 166-173.
29. McKay MJ, Castaneda M, Catania S, et al. Quantification of short-chain fatty acids in human stool samples by LC-MS/MS following derivatization with aniline analogues[J].J Chromatogr B Analyt Technol Biomed Life Sci.2023; 1217:123618.
30. Wu Y, Zheng Y, Wang S, et al. Genetic divergence and functional convergence of gut bacteria between the Eastern honey bee Apis cerana and the Western honey bee Apis mellifera[J]. J Adv Res. 2021; 37:19-31.
31. Fouts, D. E., Szpakowski, S., Purushe, J., Torralba, M., Waterman, R. C., et al. (2012). Next Generation Sequencing to Define Prokaryotic and Fungal Diversity in the Bovine Rumen. PLoS ONE, 7(11), e48289. doi:10.1371/journal.pone.0048289
32. Schloss, P. D., Gevers, D., & Westcott, S. L. (2011). Reducing the Effects of PCR Amplification and Sequencing Artifacts on 16S rRNA-Based Studies. PLoS ONE, 6(12), e27310. doi:10.1371/journal.pone.0027310
33. Quast, C., Pruesse, E., Yilmaz, P., Gerken, J., Schweer, T., Yarza, P., Peplies, J., & Glöckner,F.O.(2013).The SILVA ribosomal RNA gene database project: improved data processing and web-based tools.Nucleic Acids Research, 41(D1), D590-D596. doi:10.1093/nar/gks1219
34. Cole, J. R.,Tiedje, J. M, et al.The Ribosomal Database Project:improved alignm -ents and new tools for rRNA analysis. Nucleic Acids Research, 37(Database issue), D141-D145. doi:10.1093/nar/gkn879
35. DeSantis, T. Z., Hugenholtz, P, et al. (2006). Greengenes, a Chimera-Checked 16S rRNA Gene Database and Workbench Compatible with ARB. Applied and Environmental Microbiology, 72(7), 5069-5072. doi:10.1128/AEM.03006-05
36. Edgar, R. C. (2010). Search and clustering orders of magnitude faster than BLAST. Bioinformatics, 26(19), 2460-2461. doi:10.1093/bioinformatics/btq461
37. Wang, Q., Garrity, , et al. (2007). Naive Bayesian classifier for rapid assignment of rRNA sequences into the new bacterial taxonomy. Applied and Environmental Microbiology, 73(16), 5261-5267. doi:10.1128/AEM.00062-07
