# Supplementary material for: Segatella exacerbates chronic heart failure via TLR4/NF-κB pathway and therapeutic potential of low-carbohydrate diet
Source: Cell Death Discov. 2025 Oct 21;11:472. doi: 10.1038/s41420-025-02762-9 (PMC12541022; doi:10.1038/s41420-025-02762-9)

***Full and Uncropped Western Blots***

*****Ex vivo results*****

β-actin


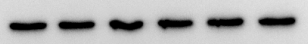


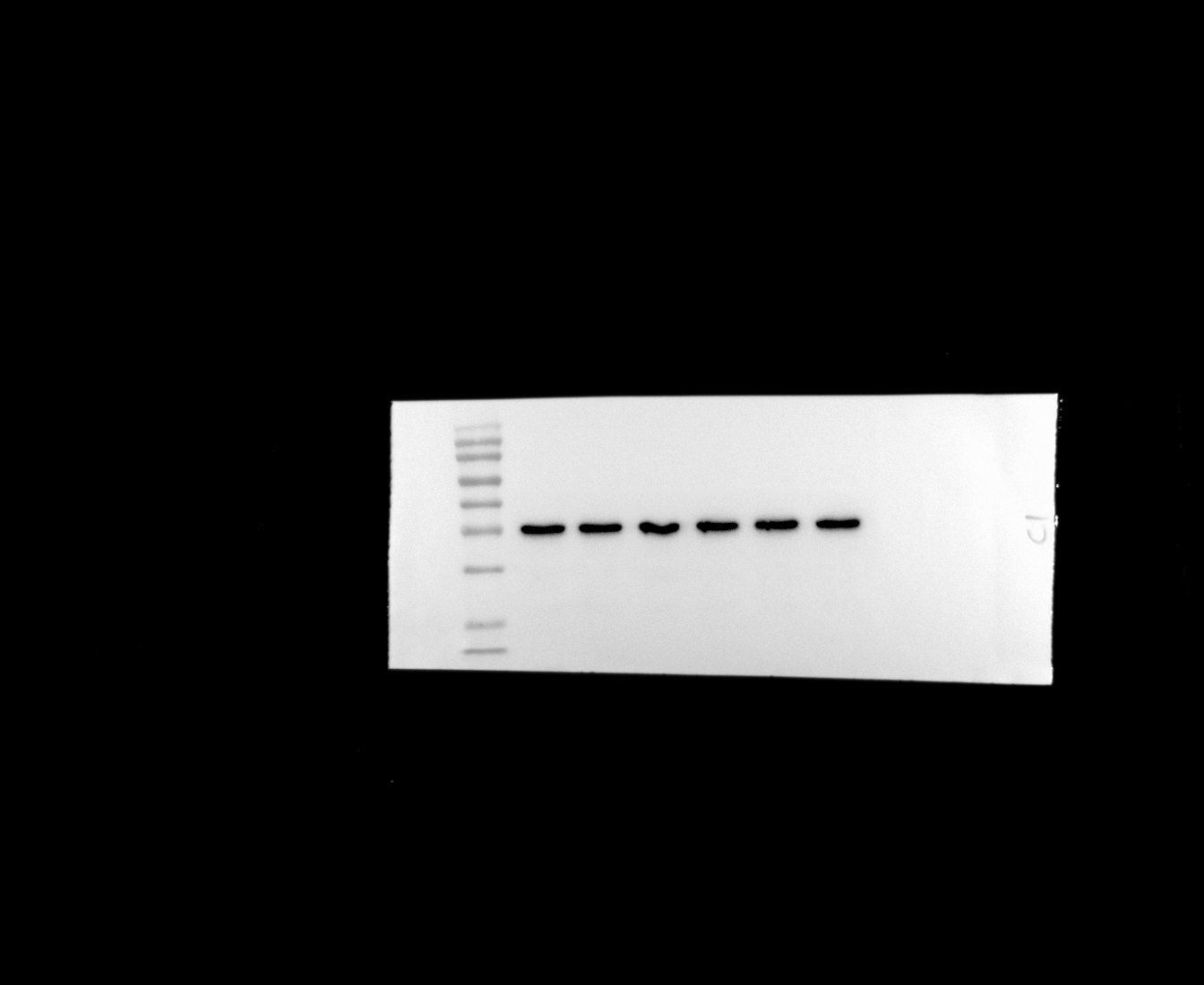


Cleaved-caspase-3


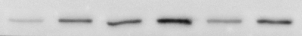


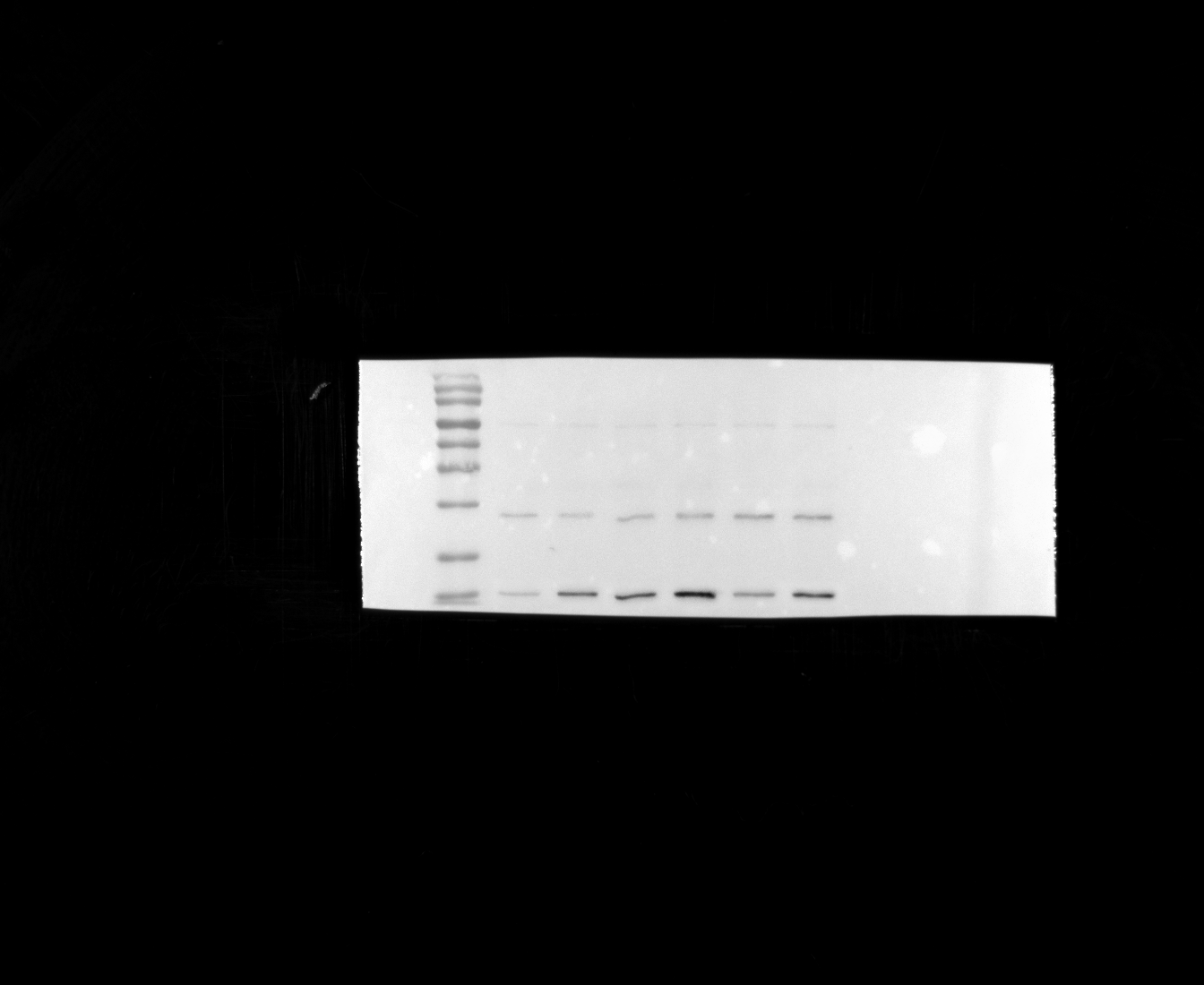


P53


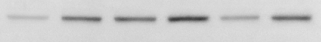


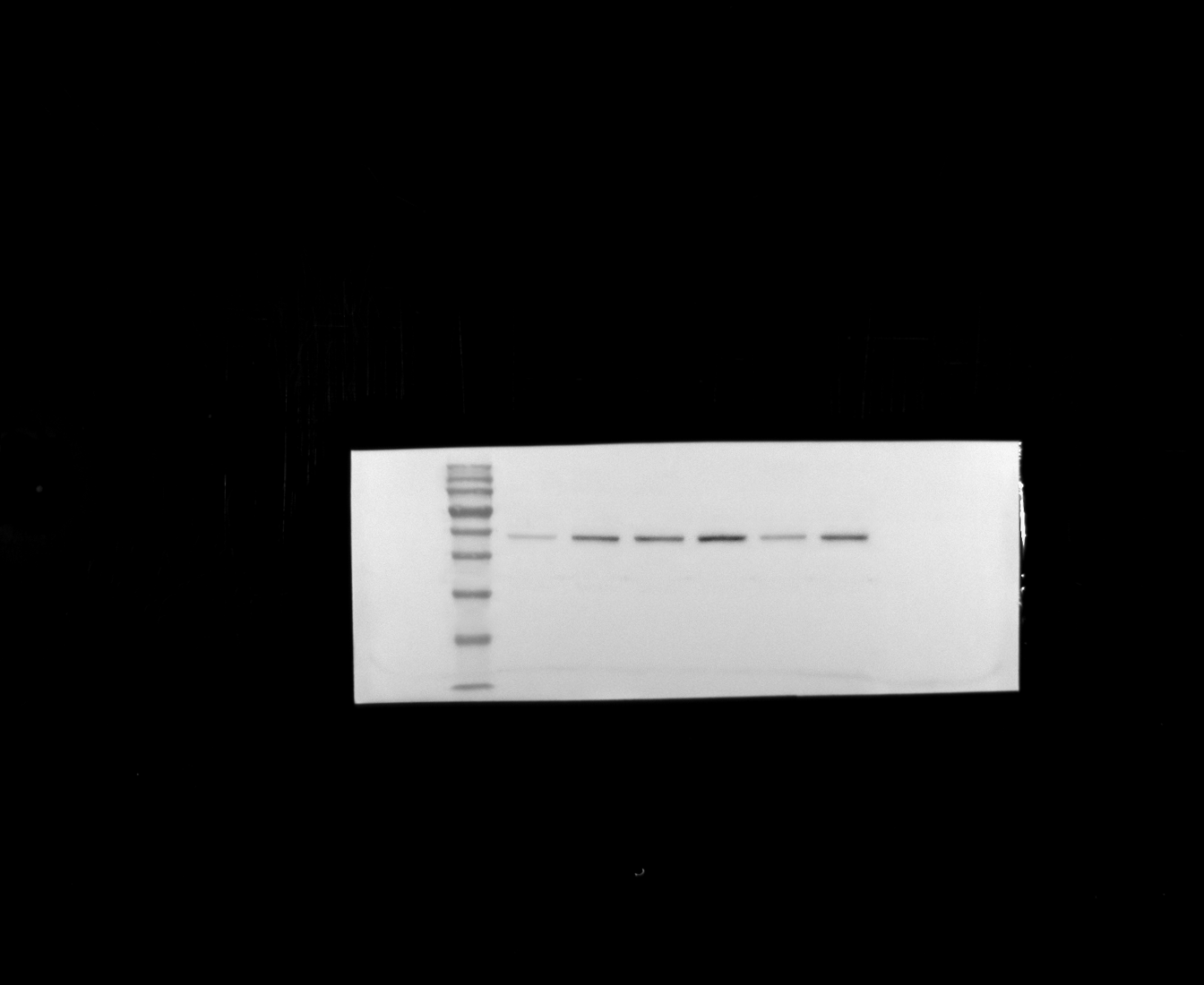


TLR4


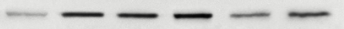


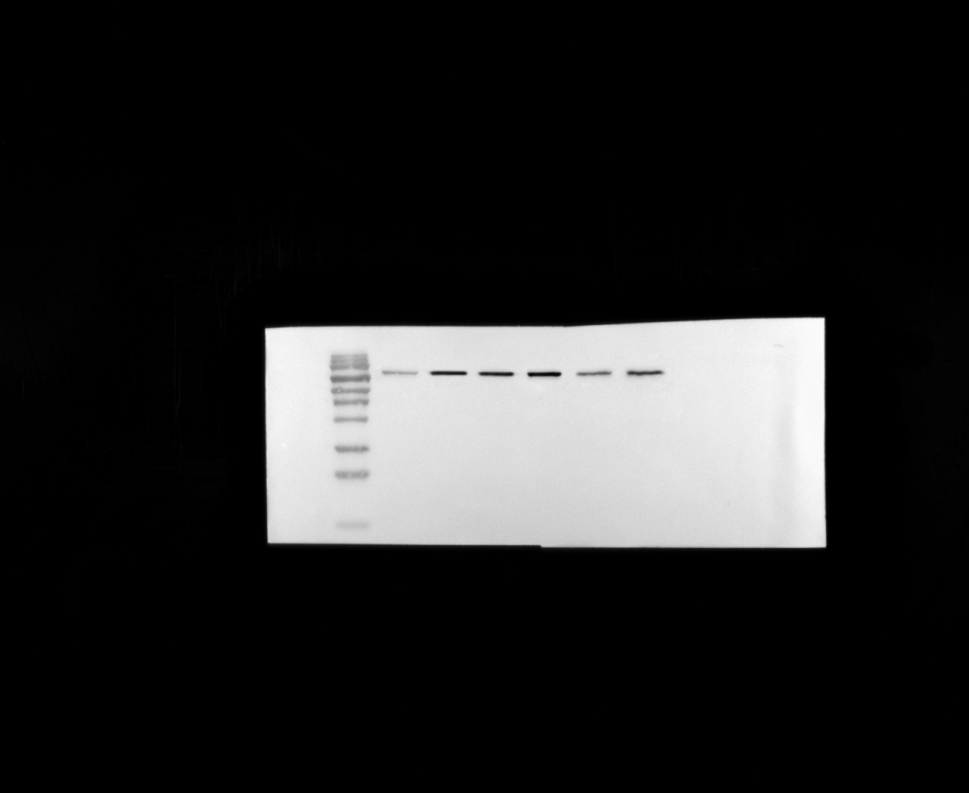


MyD88


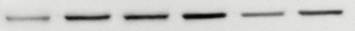


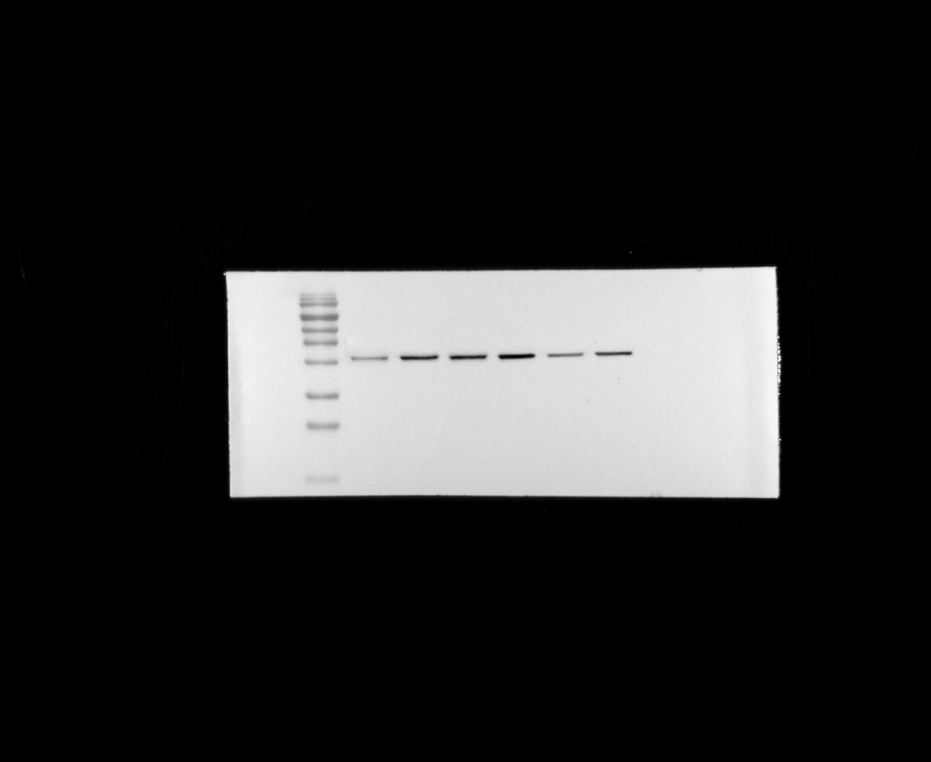


p65


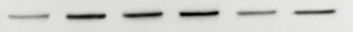


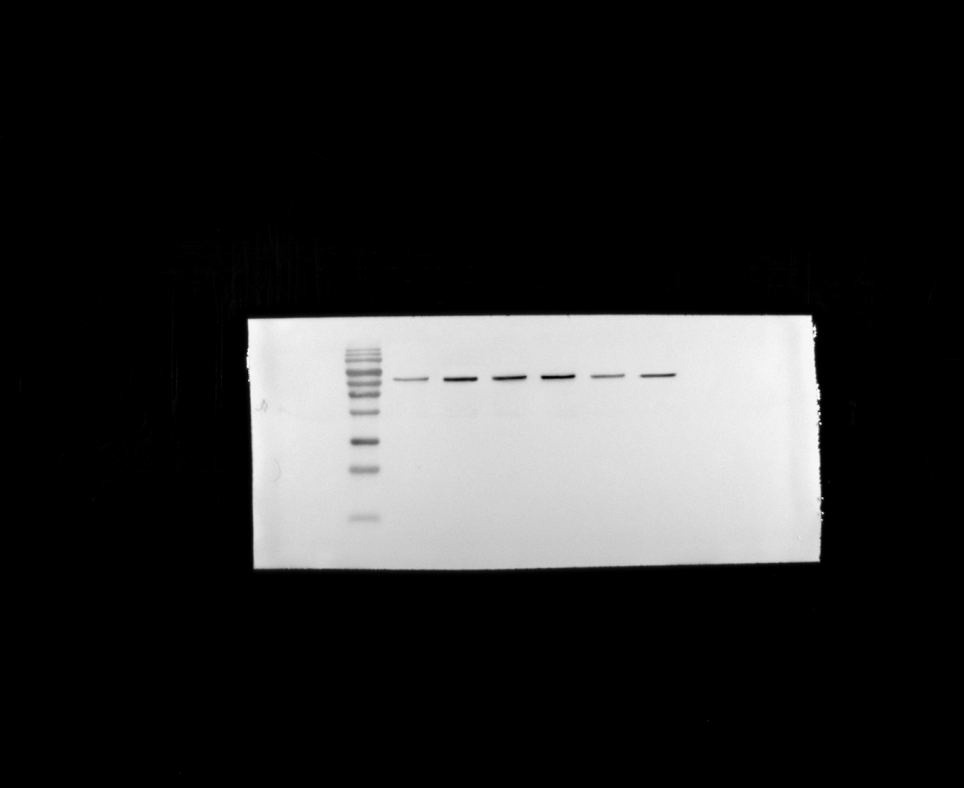


*****In Vivo Validation in Rats*****

β-actin


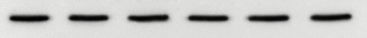


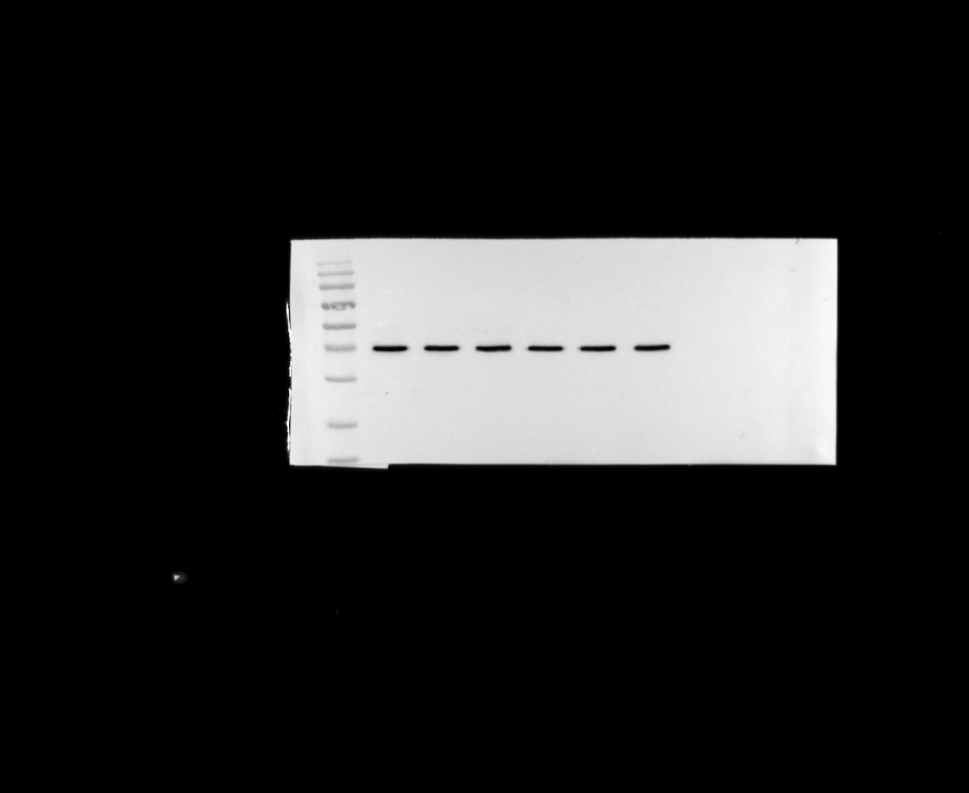


TLR4


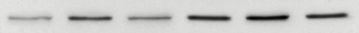


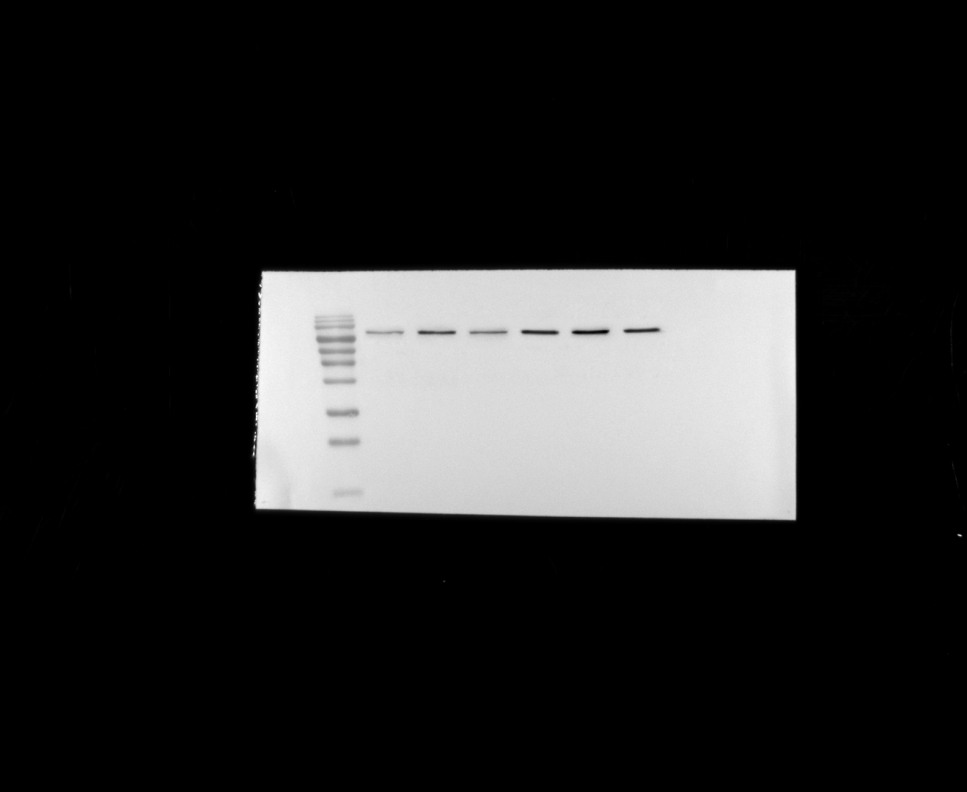


MyD88


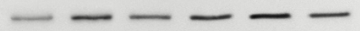


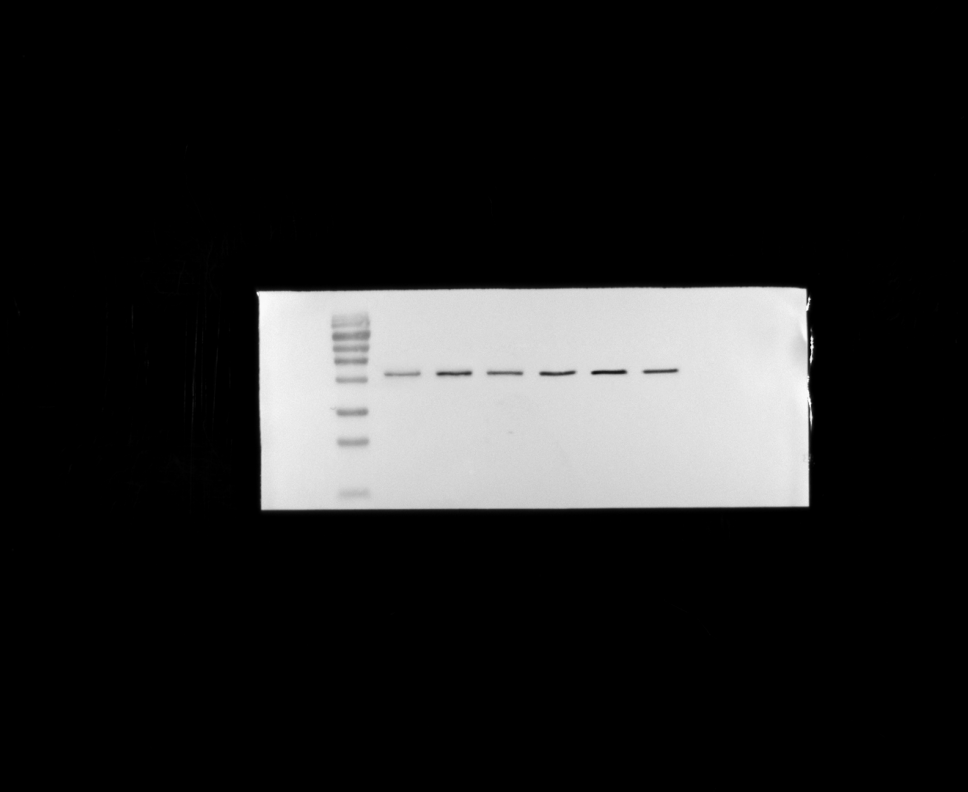


p65


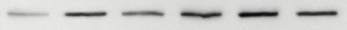


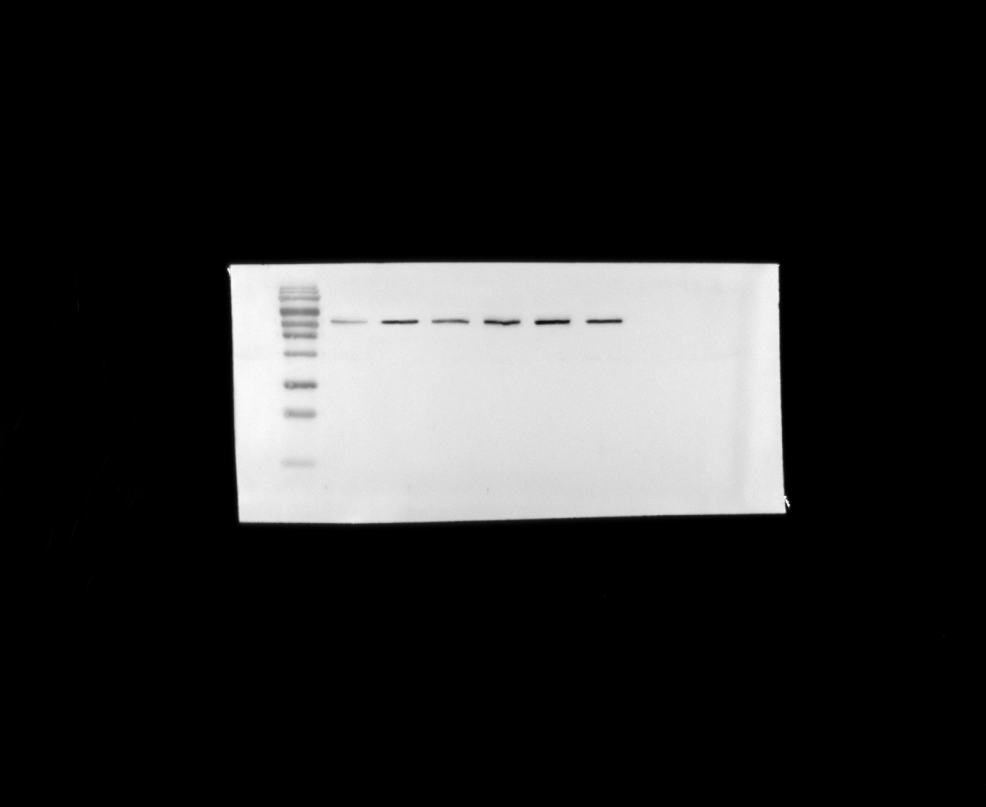

Supplement: Supplementary file 3 — Full and Uncropped Western Blots [file 41420_2025_2762_MOESM3_ESM.docx]
